# Comparison of *Pf*HRP-2/*p*LDH ELISA, qPCR and Microscopy for the Detection of Plasmodium Events and Prediction of Sick Visits during a Malaria Vaccine Study

**DOI:** 10.1371/journal.pone.0056828

**Published:** 2013-03-15

**Authors:** Ismail Mahat Bashir, Nekoye Otsyula, George Awinda, Michele Spring, Petra Schneider, John Njenga Waitumbi

**Affiliations:** 1 Walter Reed Project, Kenya Medical Research Institute, Kisumu, Kenya; 2 Division of Malaria Vaccine Development, Walter Reed Army Institute of Research, Silver Spring, Maryland, United States of America; 3 Institutes of Evolution, Immunology and Infection Research, University of Edinburgh, Edinburgh, United Kingdom; Instituto de Ciências Biomédicas/Universidade de São Paulo - USP, Brazil

## Abstract

**Background:**

Compared to expert malaria microscopy, malaria biomarkers such as *Plasmodium falciparum* histidine rich protein-2 (*Pf*HRP-2), and PCR provide superior analytical sensitivity and specificity for quantifying malaria parasites infections. This study reports on parasite prevalence, sick visits parasite density and species composition by different diagnostic methods during a phase-I malaria vaccine trial.

**Methods:**

Blood samples for microscopy, *Pf*HRP-2 and *Plasmodium* lactate dehydrogenase (*p*LDH) ELISAs and real time quantitative PCR (qPCR) were collected during scheduled (n = 298) or sick visits (n = 38) from 30 adults participating in a 112-day vaccine trial. The four methods were used to assess parasite prevalence, as well as parasite density over a 42-day period for patients with clinical episodes.

**Results:**

During scheduled visits, qPCR (39.9%, N = 119) and *Pf*HRP-2 ELISA (36.9%, N = 110) detected higher parasite prevalence than *p*LDH ELISA (16.8%, N = 50) and all methods were more sensitive than microscopy (13.4%, N = 40). All microscopically detected infections contained *P. falciparum*, as mono-infections (95%) or with *P. malariae* (5%). By qPCR, 102/119 infections were speciated. *P. falciparum* predominated either as monoinfections (71.6%), with *P. malariae* (8.8%), *P. ovale* (4.9%) or both (3.9%). *P. malariae* (6.9%) and *P. ovale* (1.0%) also occurred as co-infections (2.9%). As expected, higher prevalences were detected during sick visits, with prevalences of 65.8% (qPCR), 60.5% (*Pf*HRP*-2* ELISA), 21.1% (*p*LDH ELISA) and 31.6% (microscopy). *Pf*HRP-2 showed biomass build-up that climaxed (1813±3410 ng/mL SD) at clinical episodes.

**Conclusion:**

*Pf*HRP-2 ELISA and qPCR may be needed for accurately quantifying the malaria parasite burden. In addition, qPCR improves parasite speciation, whilst *Pf*HRP-2 ELISA is a potential predictor for clinical disease caused by *P. falciparum*.

**Trial Registration:**

ClinicalTrials.gov NCT00666380

## Introduction

Current malaria control goals are ambitious and include elimination and ultimately eradication [1]. This daunting task will require, among other things, the use of better diagnostic tools for monitoring elimination/eradication successes through detecting all malaria cases, including the huge proportions of submicroscopic parasitemias [2].

Microscopy, the gold standard for malaria diagnosis, has detection limit of 10–50 parasite/µL [3], [4]. Recent developments in molecular diagnosis have pushed the detection limits for malaria parasites to as low as 0.0004 parasite/µL using real time quantitative PCR (qPCR) [5]. Other improvements include quantitative detection of malaria antigens such as *Plasmodium falciparum* histidine rich protein-2 (*Pf*HRP-2) and *Plasmodium* lactate dehydrogenase (*p*LDH) [6], [7]. However, the increased sensitivity of newer assays continue to generate discussions [8] as to whether detected submicroscopic infections represent live or dead parasites. While such contentions are difficult to prove, there is evidence that submicroscopic parasitemia maintains chronic infections during the non-transmission season in Sudan [9] and submicroscopic infections have been shown to considerably contribute to mosquito transmission [10]. Clearly, the diagnostic sensitivity of malaria microscopy is suited to clinical cases when parasitemia is not limiting. However, elimination and eradication efforts will require superior diagnostic platforms that can accurately reveal the true extent of infections that are outside the diagnostic limits of malaria microscopy.

Like microscopy, methods such as *p*LDH ELISA and qPCR detect circulating parasites, and can therefore only account for parasitemia at the time of drawing blood and not for the sequestered parasite population. In contrast, *Pf*HRP-2 is released into circulation from all stages of *P. falciparum* parasites, and therefore is able to account for trophozoites and schizonts that are sequestered away from peripheral circulation [11], [12]. Because it persists in circulation, it can serve as an indicator of the magnitude of current or recent infection.

This paper reports on the performance of microscopy, *Pf*HRP-2 and *p*LDH ELISAs and qPCR during a malaria vaccine trial to 1) detect *Plasmodium* events; 2) determine species composition; and 3) show infection dynamics prior to sick visits and following antimalarial treatment.

## Methods

The protocol for this trial and supporting CONSORT checklist are available as supporting information; see Checklist S1 and Protocol S1.

### Study, Participants and Site

The FMP-10 phase I malaria vaccine trial was conducted between December 2008 and June 2009 at the KEMRI/Walter Reed Project Clinical Trial Centre in the Kombewa Division of Kisumu West District, western Kenya (Clinicaltrials.gov: NCT00666380). This area is holo-endemic for malaria with peak transmission occurring during the long rains (March–June) and short rains (November–December). Cumulative malaria attack rates for *P. falciparum* are about 95% during long rains and 75% during the short rains [13]. For the study, 30 clinically healthy malaria-experienced adults (18–50 years) were enrolled. These individuals were randomized into two arms, 20 in the vaccine arm and 10 controls. Blood samples (500 µL) were collected by venipuncture, weekly for the first two weeks (days 0, 7 and 14), on days 28, 35 and 42, then on days 56, 63, 70 and lastly on day 112. Each sample was used to detect malaria parasites by microscopy, qPCR, *Pf*HRP-2 and *p*LDH ELISAs. In addition, samples were taken during unscheduled sick visits, and clinical malaria episodes were treated with oral tablets of artemether/lumefantrine (20 mg/120 mg) given as follows: 4 tablets under direct observation at the time of initial diagnosis, 4 tablets after 8 hours and then 4 tablets twice daily (morning and evening) on each of the following two days. The phase I clinical trial is designed to evaluate safety and not designed or powered to evaluate vaccine efficacy. No difference was shown in the ability of serum to inhibit parasite growth in the vaccinated and control groups and therefore no attempt was made to dichotomize the study samples into vaccinated and controls.

### Ethical Statement

Written informed consent was obtained from all participants involved in the study. Scientific and ethical approvals for the study were obtained from the Kenya Medical Research Institute (KEMRI) Ethical Review Committee (KEMRI SSC # 1337) and the Walter Reed Institute of Army Research (WRAIR) Human Subject Protection Committee (WRAIR # 1417b).

### Microscopy

Microscopy examination involved thick and thin blood smears that were made as described previously [14] and stained with 3% Giemsa for 1 hour. The smears were examined by an expert microscopist who was blinded to the outcome of other assays and to the clinical condition of the participants. The microscopist counted malaria parasites against 200 white blood cells (WBCs) from the thick film if the parasite:WBC ratio was less than ≤2. Slides with parasite:WBC ratio >2 were counted against 2000 red blood cells (RBC) on the thin smear. The parasite density was obtained by assuming a total WBC count of 8000/µL and 4.5 million RBC/µL and at least 200 fields were examined before a score of negative result was entered [15]. Parasite speciation was based on morphology.

### P*f*HRP-2 and *p*LDH ELISAs

*Pf*HRP-2 and *p*LDH ELISAs were carried out according to methods described previously [6], [7]. Standard curves for *Pf*HRP-2 ELISA were generated from the *Pf*HRP-2 recombinant antigen (kind donation from Dr Sullivan, Johns Hopkins University). *p*LDH standards curves were generated from recombinant antigen supplied in the *p*LDH kit (Standard Diagnostics INC, South Korea) as a positive control. The concentration range of the *p*LDH standard curve was 0.0423 to 132 ng/mL and 1.9 to 500 ng/mL for *Pf*HRP-2.

### qPCR

For the qPCR analysis, 200 µL of EDTA blood was used to extract nucleic acids (combined DNA and RNA) using QIAamp® MinElute® Virus Spin kit (Qiagen Inc USA). The samples were eluted in 100 µL elution buffer. *P. falciparum* 3D7 ring stage parasites, obtained by D-sorbitol synchronization of a 3% parasitemia laboratory culture [6], were serially diluted in uninfected whole blood from 1500 to 0.012 parasites/µL. Dilution series were processed in the same way as samples to generate standards for parasite quantification by qPCR.

Quantitative PCR was carried out first for the genus *Plasmodium* using primers and probes targeting the 18S ribosomal RNA gene (18S rRNA) as described previously [5]. To obtain maximum sensitivity, we have followed the qRT-PCR approach as in [5], where samples with both DNA and RNA are used in reverse transcription and subsequent real-time PCR. This genus-specific qPCR was more sensitive (lower detection limit of 0.02 parasites/µL) than the species-specific qPCR (lower detection limit 0.125 parasite/uL). Therefore, speciation of malaria parasites was carried out only on samples that were positive in the genus-specific qPCR. Both genus and species-specific qPCRs were performed in a final volume of 10 µL that contained: 1 µL of template nucleic acids, 5 µL of 2× Qiagen Quantitect probe RT-PCR master mix (Qiagen Inc, USA), 0.4 µM of each primer, 0.2 µM probe, 0.1 µL of Qiagen reverse transcriptase enzyme mix, 4 mM magnesium chloride and sterile PCR grade water to make a final volume of 10 µL. Primers and probes sequences are listed in Table 1. Reactions were carried out on a 7300 thermocycler (Applied Biosystems). The amplification process started with a 30 minutes reverse transcription step at 50°C to convert RNA to cDNA. This was followed by 94°C for 10 min and 45 cycles of 95°C for 15 s, and 60°C for 1 minute to amplify the target cDNA and genomic DNA.

**Table 1:** Species-specific primers and probes for detecting *Plasmodium* parasites.

Species Type	Specific Primers and Probe Sequences
*P. falciparum*	
Forward	FAL3F AGT ACA CTA TAT TCT TAT TTG AAA TTG AA
Reverse	FAL3R TG CCT TAA ACT TCC TTG TGT TAG
Probe (5′ FAM - 3′ TAMRA)	FAL3P CTC TTC TTT TAA GAA TGT ACT TGC TTG ATT
*P. vivax*	
Forward	VIV3F GCAACGCTTCTAGCTTAATCC
Reverse	VIV3R CAAGCCGAAGCAAAGAAAGT
Probe (5′ VIC- 3′ TAMRA)	VIV3P ACTTTGTGCGCATTTTGCTA
*P. ovale*	
Forward	OVA3F TAT AGC TGA ATT TGC TTA TTT TGA AG
Reverse	OVA5R G CTT TAC AAT CAA ACG AAT ACA TTC
Probe (5′ VIC - 3′ TAMRA)	OVA3P ATA CAA TTA ATG TGT CCT TTT CCC TA
*P. malariae*	
Forward	MAL4F TT TGT ATA ATT TTT TAT GCA TGG GAA TTT TG
Reverse	MAL5R ATGCTGTAGTATTCAAACACAGAAAC
Probe (5′ FAM- 3′ TAMRA)	MAL3P TGTTCAAAGCAAACAGTTAAAACA

### Statistical analysis

All statistical analyses were performed using SAS Version 9.1 (SAS Institute Inc Cary, N. Carolina, USA). Generalized Estimating Equations for dichotomous outcome was used to test for differences between the four diagnostic methods in detecting parasite prevalence, because multiple samples of each patient were tested. *p*LDH and *Pf*HRP-2 ELISA and qPCR were compared to microscopy and between each of the methods using odds ratios. As performance of the various techniques depends on parasite density (e.g. submicroscopic densities can be detected by PCR, not microscopy, whilst higher parasite densities can be accurately detected by both methods), we have adjusted our analysis for visit day (time since start of the study), and for the interaction between visit day and method. General linear models were used to test whether parasite density estimated by the 4 methods differs with time before onset of disease, and if any time point could serve as a predictor of clinical disease. Robust standard error was used to adjust for repeated measurements.

## Results

### Prevalence of non-clinical malaria infections, measured by the four diagnostic methods

**Figure 1:**
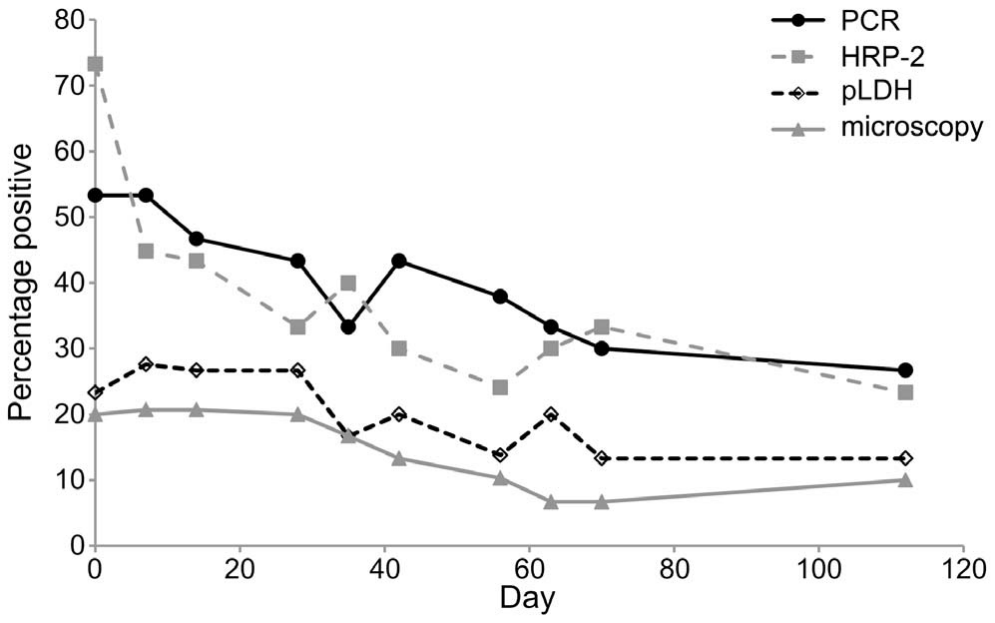
Trends in malaria prevalence by diagnostic method among the study participants that did not develop clinical malaria during the 112-day study. At every visit, malaria prevalences are highest when detected by *Pf*HRP-2 ELISA and qRT-PCR methods and lowest when measured with microscopy and *p*LDH ELISA.

We have compared the diagnostic value of 4 methods (microscopy, qPCR, *p*LDH and *Pf*HRP-2 ELISA) for the detection of malaria parasites during a vaccine trial. Samples were not dichotomized into vaccinated and control groups as the study showed no differences between the groups. Blood samples of 30 individuals were collected during scheduled visits (n = 298) and were evaluated for parasitemia events by the 4 different diagnostic methods. There were significant differences between the 4 methods in detecting malaria parasite prevalence. Using data from adjusted odds ratio, qPCR (prevalence 39.9%, confidence interval 34.4–45.5%) and *Pf*HRP-2 ELISA (36.9%; CI 31.4–42.4%) performed similarly (*P* = 0.80) and both detected higher prevalences compared to *p*LDH ELISA (16.8%; CI 12.5–21.0%) (*P*<0.0001). Microscopy (13.4%; confidence interval (CI) 9.6–17.3%) detected lower prevalences than the three other methods (*P*<0.05) (Table 2; Figure 1).

**Table 2:** Pair-wise Comparison of malaria diagnostic methods using Generalized Estimating Equations (GEE).

Comparison	Adjusted for visit day and interaction between method and visit	
	OR (95% CI)	P-value
qPCR vc Microscopy	1.35 (1.20–1.52)	0.0001
qPCR vs pLDH ELISA	1.28 (1.13–1.45)	0.0001
qPCR vs *Pf*HRP-2 ELISA	0.99 (0.90–1.09)	0.8005
*Pf*HRP-2 ELISA vs Microscopy	1.37 (1.24–1.51)	0.0001
*Pf*HRP-2 ELISA vs pLDH ELISA	1.30 (1.16–1.45)	0.0001
pLDH ELISA vs Microscopy	1.05 (1.00–1.11)	0.0309

### Prevalence of malaria parasite species by microscopy and qPCR

Table 3 shows the species of malaria parasites that were identified during scheduled visits by microscopy and species-specific qPCR during the 112-days of study. *P. falciparum* was the most common malaria parasite species and was present in all samples diagnosed positive by microscopy (N = 40), either as mono-infections (95%, N = 38) or in combination with *P. malariae* (5%, N = 2). Of the 119 samples positive by genus qPCR, 102 were successfully amplified by the species-specific qPCRs. *P. falciparum* was present in 89.2% of these infections, 71.6% (N = 73) as mono infection, 8.8% (N = 9) with *P. malariae*, 4.9% (N = 5) with *P. ovale* and 3.9% (N = 4) with *P. malariae* and *P. ovale*. *P. malariae* and *P. ovale* mono-infections accounted for 6.9% (N = 7) and 1.0% (N = 1) respectively and 2.9% (N = 3) for co-infections. *P. vivax* was not detected in any of the samples.

**Table 3:** The number and percentage of *Plasmodium* parasite species detected by microscopy and qPCR.

Species	Microscopy (%)	qPCR (%)
*Pf*	38 (95)	73 (71.6)
*Pf+Pm*	2 (5)	9 (8.8)
*Pf+Po*	0	5(4.9)
*Pf+Po+Pm*	0	4 (3.9)
*Pm*	0	7 (6.9)
*Po*	0	1 (1.0)
*Po+Pm*	0	3 (2.9)
Total	40	102

Key: Pf = *P. falciparum*, Pm = *P. malariae*, Po = *P. ovale*.

### Utility of microscopy, qPCR, P*f*HRP-2 and *p*LDH ELISAs in predicting clinical episodes

We have evaluated the utility of the 4 diagnostic methods in quantifying malaria parasite load in patients with clinical signs suggestive of malaria. A total of 38 acute care malaria blood smears were requested by the clinical team over the 112 days follow-up period for study volunteers presenting with any of the following indicators of malaria: fever (ancillary temperature >37.5°C), headache, backache, malaise and generalized body pain. Blood samples from these patients were analyzed by microscopy, *Pf*HRP-2/*p*LDH ELISAs, and qPCR. Only twelve samples (31.6%) had microscopically confirmed malaria parasites and these patients were treated with artemether/lumefantrine as described in the methods section. Following treatment, patients continued to be evaluated for malaria during the scheduled visits.

**Figure 2:**
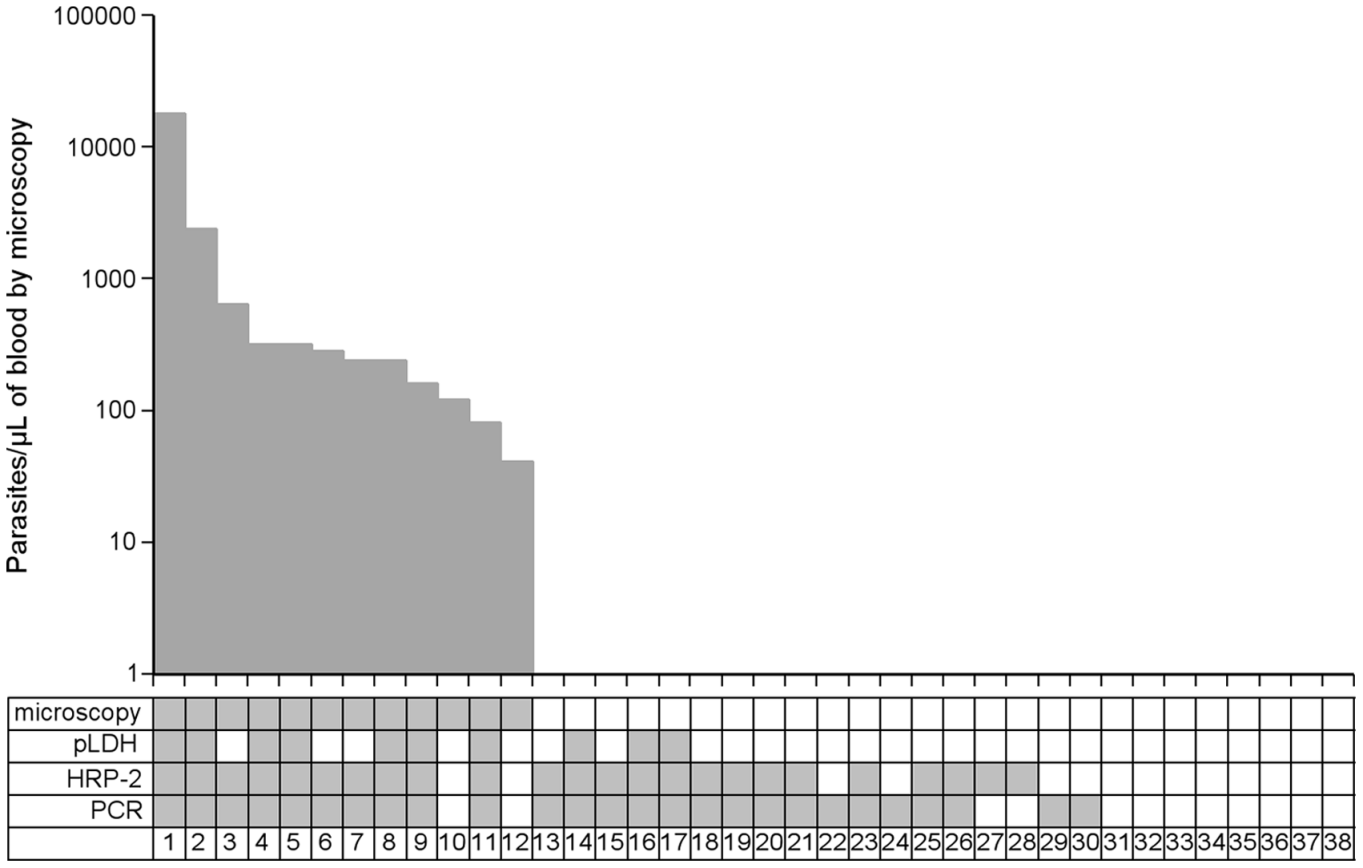
Comparison of routine microscopy, *p*LDH/*Pf*HRP-2 ELISA and qPCR for a group of study participants who had acute blood smears prepared at sick visits. Each column (1–38) represents one blood sample with the corresponding microscopy, *p*LDH/*Pf*HRP-2 ELISA and qRT-PCR results, ordered by parasite density as determined by microscopy (top graph) and antigen levels (*p*LDH/*Pf*HRP-2) or Ct values (qPCR). As the levels of parasitemia decreases, the concordance between the different methods also decreases. *Pf*HRP-2 and qPCR detect parasites densities way beyond the detection limit of microscopy.

As participants were clinically unwell, the probability of malaria parasites in samples taken during these visits is higher, with prevalences of 65.8% (qPCR), 60.5% (*Pf*HRP*-2* ELISA), 21.1% (*p*LDH ELISA) and 31.6% (microscopy). Figure 2 shows the concordance of malaria parasitemias by the four methods in the 38 individuals who reported being sick. As the levels of parasitemia determined by micropscopy decreases, the concordance between the different methods decreases as expected. For the 12 participants who had microscopic confirmed malaria, sensitivity (the number of positive samples detected correctly) was 10/12 (83.3%) for both the genus-specific qPCR and *Pf*HRP-2 ELISA and 7/12 (58.3%) for the *p*LDH ELISA. Two microscopy confirmed cases were negative for all three other diagnostic methods, suggesting that the parasites identified by microscopy may have been misdiagnosis, which would suggest sensitivities to be 70% (*p*LDH) and 100% (qPCR and *Pf*HRP-2 ELISA). In addition, *Pf*HRP-2 ELISA and qPCR detected over double the amount of malaria cases detected by microscopy and *p*LDH ELISA, most likely as these methods detect parasites densities way beyond the detection limit of microscopy and *P*LDH ELISA; also *Pf*HRP-2 ELISA detects the antigen secreted by parasites in previous multiplication cycles within the infection, allowing an estimate of cumulative parasite biomass.

**Figure 3:**
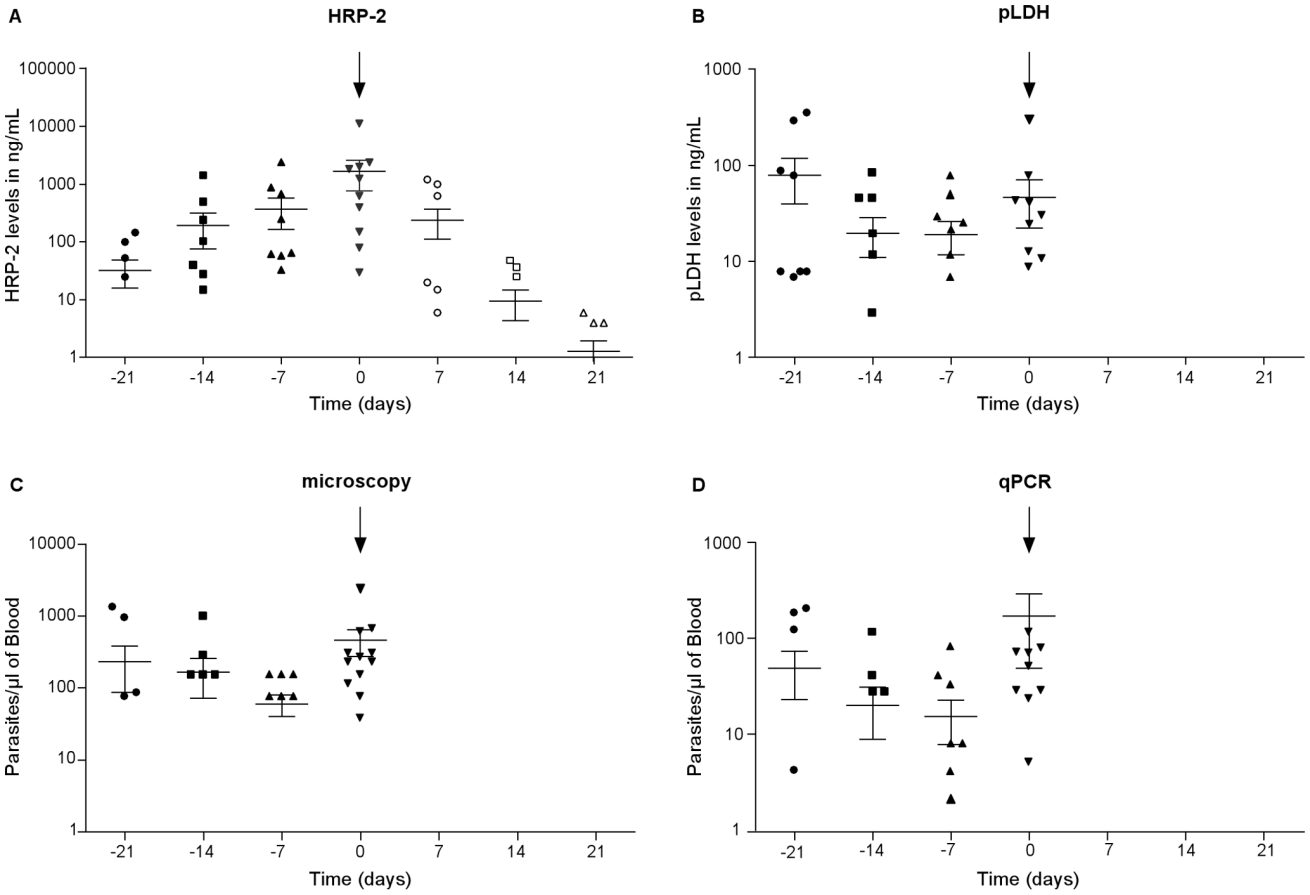
Utility of microscopy, qPCR, PfHRP-2 and pLDH ELISAs in predicting clinical episodes. Parasite dynamics before clinical malaria attack (**day 0**) as measured by (A) *Pf*HRP-2, (B) *p*LDH (C) Microscopy and (D) qPCR, for the 12 participants with microscopically confirmed clinical malaria. Parasite dynamics after clinical attack are also presented for *Pf*HRP-2 (A). Error bars represent standard error of mean of the parasitemia values at each time point. The arrows indicate the day of treatment. Microscopy, *p*LDH and qPCR did not detect malaria parasites after the treatment.

Figure 3 show the parasite dynamics estimated by the four malaria diagnostic methods at days 21, 14, 7 and 0 before and after anti-malarial treatment for the 12 individuals who had microscopically confirmed clinical malaria. General linear model was used to test for differences in calculated parasite densities before onset of disease. This analysis determined if any of the days could serve as a reliable predictor of clinical disease in a prospective study. *Pf*HRP-2 ELISA, but not the other methods, shows a cumulative build-up of parasite antigen that reached a maximum concentration (mean = 1813±3410 ng/mL SD) on the day the patients reported being sick. Following anti-malarial treatment (day 0), *Pf*HRP-2 levels declines slowly and remains detectable up to 21 days post treatment, whilst the other methods cannot detect parasites or parasite products on days 7, 14 and 21 post treatment. As shown in Figure 2, there were 26 individuals who reported sick without having malaria parasites detectable by microscopy. However, 16 of these individuals had submicroscopic parasitemia by *Pf*HRP-2 and/or qPCR. Their mean *Pf*HRP-2 levels at the day of presentation (day 0) was 260 ng/mL and respectively 134 ng/mL, 253 ng/mL and 12 ng/mL on days 7, 14 and 21 before disease onset.

## Discussion

This study provides a dataset for judging the performance of microscopy, *Pf*HRP-2 and *p*LDH ELISAs and qPCR for detecting *Plasmodium* events and evaluates parasite density build-up that culminates in malaria attributable sick visits in a cohort participating in a 112-day malaria vaccine trial. Each of these methods has particular attributes that appeal to different end users. Microscopy is the gold standard for malaria diagnosis, it is cost effective and simple to use but has a low sensitivity. When not done well, its main drawback includes poor reproducibility, variable sensitivity, and unacceptably high false-positive rates [6], [16]–[19]. The glycolytic *p*LDH is a secreted antigen that is found in all *Plasmodium* species, and because it is short lived in blood, it is a good indicator of current infection. However, *p*LDH is produced in small quantities and this limits its sensitivity, especially for the non *P. falciparum* species whose parasitemias are usually low. In contrast, *Pf*HRP-2 (produced by only *P. falciparum*) [4] has a higher sensitivity because it is produced in large quantities and by all stages of *P. falciparum* parasites [20]. For these reasons, *Pf*HRP-2 is the mainstay for *P. falciparum* rapid diagnostic tests. *Pf*HRP-2 is water soluble and its blood level originates from parasites in the peripheral circulation as well as from those sequestered in endothelial vessels and in organs such as liver, kidneys, brain and placenta [11], [12]. In addition, *Pf*HRP-2 has a long half-life and therefore can account for historical parasitemia. Because of these attributes, circulating levels of *Pf*HRP-2 reflect the total cumulative parasite biomass [11] and could serve as an indicator of the magnitude of recent (and potentially already cured) infection. Molecular methods are considerably more sensitive than most diagnostic methods, and we have used an approach that has superior sensitivity as a result of detecting the combined RNA and DNA of 18S rRNA gene, allowing us to detect submicroscopic parasitemia as low as 0.02 parasite/µL [5], [21]. Like microscopy and *p*LDH, but unlike *Pf*HRP-2, qPCR only detect current parasitemias and therefore cannot account for diurnal fluctuation or for mature trophozoite and schizont stages that are unavailable in the peripheral circulation.

While the number of subjects taking part in the study is small (N = 30), the numbers of samples available for analysis for the 112 days follow-up period is large (N = 298), and by using statistical methods that account for repeated measurements, we were able to fully benefit from this sample size. Our results show that microscopy detects the lowest proportion of individuals with non-clinical malaria (13.4%), followed by pLDH ELISA (16.8%) and *Pf*HRP-2 ELISA (36.9%) and qPCR (39.9%) (Figure 1, Table 2). Although prevalences were higher during sick visits by all methods tested, the sensitivity ranking of the methods did not change. The similarity in sensitivity between microscopy and *p*LDH has been shown before and is attributed to the fact that the enzyme activity parallels the levels of peripheral parasitemia [22], [23]. The qPCR approach used in this study utilized combined RNA and DNA to amplify the *Plasmodium* multicopy 18S rRNA genes that allows detection of submicroscopic parasitemia [5]. As shown previously in an area with predominantly *P. falciparum* malaria infections [24], our study reveals comparable sensitivity for measuring *P. falciparum* prevalence by *Pf*HRP-2 detection and prevalence of all *Plasmodium* species as detected by qPCR (36.9% vs 39.9% respectively). However, some studies have reported lower sensitivity of *Pf*HRP-2 assays compared to PCR in pregnant women at delivery [25], [26]. As lower concordance between PCR and *Pf*HRP-2 assays is regularly observed [24]–[26], lower performance of HRP-2 may partly be due to low parasite densities resulting from IPTp treatment. One potential setback in the use of *Pf*HRP-2 as a diagnostic tool is that reported *Pf*HRP-2 gene deletions in parasites from South America [27] and, more recently, from Sub-Saharan Africa [28] result in false negative results for this test.

At the baseline of our study, *p*LDH ELISA and microscopy indicated that approximately 20% of the participants had malaria parasites (Figure 1), whilst 53% were parasitamic by qPCR and over 70% by *Pf*HRP-2 ELISA. This enrollment parasitemia reflects the timing at the end of the short transmission season with 33% of infections being submicroscopic (*Pf*HRP-2 positive and qPCR positive) and a considerable proportion of individuals having signs of recent but not current infection (*Pf*HRP-2 positive but qPCR negative). The parasite prevalence declines over time, with more substantial declines measured by *Pf*HRP-2 ELISA (45%) and qPCR (15%), compared to almost no decline for microscopy and *p*LDH ELISA (Figure 1). Whereas we would expect such a decline in a prospective study that proactively treats clinical malaria every time it is reported, the disproportional decline between the methods suggests that this decline does not result from a reduction of clinical malaria, but from a reduction of evidence of recent infection and a decrease of the prevalence of submicroscopic infections over time.

Clearly, microscopy detects only the proverbial ears of the hippo while the massive body of parasites remains sub-microscopic. These data are corroborated by other studies that show that microscopy considerably underestimates parasite rates by up to 50% [29]–[32]. When accounting for the potential occurrence of mutations of the *Pf*HRP-2 gene which may decrease sensitivity of HRP-2 assays [27], [28] and its potential lower sensitivity in some [25], [26] but not all studies [this study, 24], the highly sensitive detection methods of *Pf*HRP-2 ELISA and qPCR will especially be useful for detecting malaria infections in the context of malaria elimination and eradication, when every infected, and thus potentially infectious, individual will need to be targeted by control measures. One potential cause of discrepant sensitivity in *Pf*HRP-2 is the choice of samples used in different studies (whole blood, plasma or serum). Our unreported data show that, malaria infected red blood cells have up to 10 times more *Pf*HRP-2 than in the plasma. In the future, and as part of standardizing of these assays, sample choice and genetic polymorphisms will need to be evaluated.

In order to understand the infection dynamics that herald clinical malaria, blood samples of the 12 individuals who were treated on the basis of microscopic diagnosis were evaluated by the different diagnostic techniques for parasite densities at 21, 14, 7 and 0 days before and after anti-malaria treatment. Of the four tested techniques, only *Pf*HRP-2 ELISA demonstrates a cumulative parasitemia that is highest at the time patients reported to investigators with clinical malaria and high parasite densities. For this reason, and as reported recently [33], *Pf*HRP-2 levels may be useful for predicting potential clinical episodes in prospective research studies. In the Hendriksen study [33], a plasma *Pf*HRP-2 concentration of >1000 ng/mL was shown to represent “true” severe malaria and had a case fatality rate of >10%. In our study, we are proposing that a blood *Pf*HRP-2 concentration of >1813 ng/mL as an indicator of a true clinical malaria. In comparison, 26 individuals who reported sick but did not have detectable malaria parasites by microscopy showed mean *Pf*HRP-2 levels at the day of presentation (day 0) that was four times lower than those with clinical malaria. Although 16 of these individuals had submicroscopic parasitemia by *Pf*HRP-2 and/or qPCR (Figure 2), these parasite densities (less than 1 parasite/200 WBC count on a thick blood film) ( = <40 parasites/µL of blood) are unlikely to have been the cause of illness in these malaria experienced semi immune adults. Clearly, more detailed studies are required to determine from what time point before the onset of clinical disease *Pf*HRP-2 increases, and how variable this period and *Pf*HRP-2 levels are. *Pf*HRP-2 remained in circulation for 3 weeks post successful malaria cure. This is consistent with the documented long half-life of *Pf*HRP-2 [34]–[37]. As would be expected for microscopy, pLDH and qPCR which measure biomarkers that do not accumulate, parasite densities were at undetectable levels from the first samples taken (seven days) post treatment.

One of the main benefits of PCR and microscopy is that both methods can be used for speciation of *Plasmodium* in clinical samples. As shown in Table 3, and previous reports [38]–[40], *P. falciparum* was the dominant malaria parasite species in our area. In addition to underestimating prevalence, it is clear that microscopy grossly underestimates the extent of parasite species resident in the population [41], [42]. It is known that co-infections of different malaria parasite species lead to dominance of one species over the other(s) [43]. The low parasite density of the suppressed parasite species presents diagnostic challenges for the less sensitive techniques such as microscopy. Given that malaria parasites such as *P. ovale* and *P. vivax* form dormant hypnozoites [44] that are not treatable by the conventional antimalarials, these minority parasite species may disproportionally benefit from treatment-driven elimination of the dominating parasite species, analogous to competitive facilitation [45]. It is therefore very important to identify submicroscopic parasitemia of these species for radical cure to be effective. Whereas the qPCR may not be a convenient tool for patient management in malaria endemic areas where semi immune individuals carry low grade parasitemia of no clinical consequence, this technique will provide a valuable addition for monitoring submicroscopic parasitemia in the context of evaluating the newly stated goals of malaria elimination and eventually eradication [1].

## Conclusions

The study described here reports on the combined performance of four diagnostic methods of malaria for monitoring parasite prevalence in a longitudinal study set up. The findings illustrate scores of submicroscopic *Plasmodium* infections that go undetected by microscopy. As previously suggested [29]–[32], malaria microscopy is therefore inaccurate as a tool for monitoring malaria prevalence, especially when low parasite densities are common. As the world aims to move towards the stated agenda of malaria elimination and subsequent eradication, malaria detection will shift from monitoring clinical disease to screening for asymptomatic carriers. Diagnostic methods such *Pf*HRP-2 ELISA and qPCR with the analytical sensitivity documented in this and other studies [5 and 6) will be needed for accurate evaluation of the stated goals.

## Supporting Information

Checklist S1 CONSORT Checklist.
(DOC)

Protocol S1 Trial Protocol.
(DOC)
