# Supplementary material for: Comparison of PfHRP-2/pLDH ELISA, qPCR and Microscopy for the Detection of Plasmodium Events and Prediction of Sick Visits during a Malaria Vaccine Study
Source: PLoS One. 2013 Mar 15;8(3):e56828. doi: 10.1371/journal.pone.0056828 (PMC3598859; doi:10.1371/journal.pone.0056828)
Supplement: Checklist S1 — CONSORT Checklist. (DOC) [file pone.0056828.s001.doc]

**CONSORT 2010 Flow Diagram**

**Allocation**

**Analysis**

**Follow-Up**

**Enrollment**

Assessed for eligibility (n=115)

Excluded (n=81)

♦  Not meeting inclusion criteria (n=81)

♦  Declined to participate (n=2 )

♦  Other reasons (n=2, did not show up for vaccination)

Analysed (n=19)
♦ Excluded from analysis (give reasons) (n=0)

Lost to follow-up (give reasons) (n=0)

Discontinued intervention (give reasons) (n=1 withdrawn due to elapsed vaccination window)

Allocated to intervention (n=20)

♦ Received allocated intervention (n=20 )

♦ Did not receive allocated intervention (give reasons) (n=0)

Lost to follow-up (give reasons) (n=0)

Discontinued intervention (give reasons) (n=0)

Allocated to intervention (n=10)

♦ Received allocated intervention (n=10)

♦ Did not receive allocated intervention (give reasons) (n= )

Analysed (n=10)
♦ Excluded from analysis (give reasons) (n=0)

Randomized (n=30)
