# Supplementary material for: Comparison of PfHRP-2/pLDH ELISA, qPCR and Microscopy for the Detection of Plasmodium Events and Prediction of Sick Visits during a Malaria Vaccine Study
Source: PLoS One. 2013 Mar 15;8(3):e56828. doi: 10.1371/journal.pone.0056828 (PMC3598859; doi:10.1371/journal.pone.0056828)
Supplement: Protocol S1 — Trial Protocol. (DOC) [file pone.0056828.s002.doc]

**WRAIR PROTOCOL # 1417b KEMRI SSC #1337**

# protocol Title

Phase 1b controlled, double blind Study to Evaluate the Safety, Reactogenicity, and Immunogenicity of the Candidate *Plasmodium falciparum* Malaria Protein 010 (FMP010) Administered Intramuscularly with GSK Biologicals’ Adjuvant AS01B.

**IND No**. BB-IND **13638**

**IND Sponsor** Office of the Surgeon General

Department of the Army

| **Protocol Tracking Number** | WRAIR IRB Log No. 1417b  GSK ETrack Protocol No. 109487 (Malaria- 054) |
| --- | --- |
|  |  |

# Clinical Investigators

***Principal Investigator, Part B, USAMRU-K:***

Nekoye N. Otsyula US Army Medical Research Unit - Kenya

Unit 64109 (MRU)

APO AE 09831-4109

Tel (Within Kenya): 0722 239 828

Tel (From Outside Kenya): +254-722 239 828

Email: notsyula@wrp-ksm.org

***Co-Principal Investigator***

Mark E. Polhemus, LTC, MC Division of Malaria Vaccine Development

Walter reed Army Institute of Research

503 Robert Grant Ave

Silver Spring, MD 20910

Ph 301 319 9783

Cell 202 446 4214

**Associate Investigators:**

Douglas Walsh, COL, MC US Army Medical Research Unit – Kenya

Bernhards Ogutu, M.D. US Army Medical Research Unit – Kenya

Lucas Otieno, M.D. US Army Medical Research Unit – Kenya

Ben Andagalu,M.D. US Army Medical Research Unit - Kenya

Godfrey Allan Otieno US Army Medical Research Unit – Kenya

Walter Otieno, MD US Army Medical Research Unit – Kenya

George F. O. Okoth US Army Medical Research Unit – Kenya

Michele Spring, MD DMVD, WRAIR **(Protocol champion)**

Christian F. Ockenhouse, COL, MC DMVD, WRAIR

D. Gray Heppner, COL, MC DMVD, WRAIR

James F. Cummings, LTC, MC Department of Clinical Trials, WRAIR

**Laboratory Associate Investigators:**

John Waitumbi, PhD US Army Medical Research Unit – Kenya

Ann Stewart, PhD US Army Medical Research Unit – Kenya

Evelina Angov, PhD DMVD, WRAIR

Elke Bergmann-Leitner, PhD DMVD, WRAIR

Brent House DMVD, WRAIR

Urszula Krzych DMVD, WRAIR

***Research Associates:***

Carter L. Diggs, MD, PhD US Agency for International Development

Lorraine A. Soisson, PhD US Agency for International Development

**Medical Monitor (USAMRU-K)** Dr Amos Otedo

Consultant Physician/Gastroenterologist

Box 40100-4685, Kisumu Kenya

Tel: +254 722 866 052

Email: otedoamos@yahoo.com

| **USAMMDA Representative**  Denise A. McKinney, RN, CCRC, CCRA  Clinical Trial Monitor (Contractor – CRM)  Division of Regulated Activities and Compliance  U. S. Army Medical Research & Materiel Command  P.O. Box 123-40123  Kisumu, Kenya  Landline:  +254 – (0)57- 2023725  Fax:     +254 – (0)57- 2026416  Mobile:  +254 – (0)733-333-013  dmckinney@wrp-ksm.org  <https://mrmc.amedd.army.mil/usammda/index.cfm> |  |
| --- | --- |
| **GSK Clinical Development Manager** **and Medical Monitor**  Olivier Godeaux, MD  GSK Biologicals,  Rue de l'Institut, 89  1330Rixensart,Belgium Tel: 32-2-656 6910  Fax: 32-2-656 8044  email: [Olivier.godeaux@gskbio.com](mailto:Olivier.godeaux@gskbio.com)  **GSK Central Study Coodinator**  Evi de Ruymaeker  Rue de I’Institut,89  1330 Rixensart, Belgium  Tel: 32-2-656 3760  Fax : 32-2-656 8044  *Evi.deruymaeker@gskbio.com* |  |

**GSK Site Monitor**

# INVESTIGATORS’ SIGN IN

| I, the undersigned, have reviewed this protocol, including Appendices, and I agree to conduct the clinical study as described and will adhere to the Ethical and Regulatory Considerations stated. I have read and understood the contents of the Investigator’s Brochure. |
| --- |
| Nekoye N. Otsyula, MD |
| Signature |
| Date |
| I, the undersigned, have reviewed this protocol, including Appendices, and I agree to conduct the clinical study as described and will adhere to the Ethical and Regulatory Considerations stated. I have read and understood the contents of the Investigator’s Brochure. |
| Mark E. Polhemus, MD |
| Signature |
| Date |

# SYNOPSIS

**Title**

**Overall protocol**

Phase 1a/b Study to Evaluate the Safety, Reactogenicity, and Immunogenicity of the Candidate *Plasmodium falciparum* Malaria Protein 010 (FMP010) Administered Intramuscularly with GSK Biologicals’ Adjuvant AS01B.

**Part B**

Phase 1b controlled, double blind Study to Evaluate the Safety, Reactogenicity, and Immunogenicity of the Candidate *Plasmodium falciparum* Malaria Protein 010 (FMP010) Administered Intramuscularly with GSK Biologicals’ Adjuvant AS01B.

# Background:

This is the second part of the protocol listed above. The study is being conducted in two parts at two different sites. Part A (the Phase 1a portion) is being conducted at the Walter Reed Army Institute of Research in the USA. Part B (The Phase 1b portion) is being conducted at the Walter Reed Project/Kenya Medical Research Institute in Kisumu, Kenya. This covers Part B which will be conducted in Kisumu, Kenya.

The vaccination groups in Part A will be called Group 1 and Group 2. For ease of identification, the vaccination groups in Part B will be called Group 3 and Group 4.

# Part B: Indication/ Study Population

Healthy, malaria-experienced adults living in western Kenya aged 18-50 years

# Study Rationale

The approach to developing an asexual blood stage vaccine against *P. falciparum* malaria using Merozoite Surface Protein 1 (MSP1) antigen from the surface of the merozoite stage of the parasite is founded on the hypothesis that such a vaccine will elicit strong neutralizing humoral immune responses directed against epitopes located within the 42-kDa carboxy-terminal region (MSP1_42_) of the MSP1 molecule. The main purpose of such a response is to decrease the load of infectious merozoites invading erythrocytes. Indeed, the medical literature suggests that these antibody responses targeting the surface of blood stage merozoites are important components of a protective immune response, and that the MSP1 antigen, and more specifically the MSP1_42_ fragment is one of the targets of this response (1). Ideally, the immune responses generated by priming would be recalled and increased by administering booster vaccinations, as well as by natural infections. Good and Doolan (2) reported that acquired protective immunity against blood stages of malaria involved both Ab- and cell-mediated immunity. Since B-cell epitopes localized on the MSP1_19_ part of the molecule (3) and T-cell helper regions localized on the MSP1_33_ (4) comprise the MSP1_42_ vaccine candidate, this molecule may be capable of inducing both proliferative and cytokine T cell helper responses required to confer protection mediated through helper functions in the production of antibodies or by direct secretion of effector lymphokines such as interferon gamma. The vaccine should, therefore, be capable of inducing T-helper memory responses.

Although MSP1-based vaccines such as the 3D7 allele have been tested previously in humans, this is the first time that this candidate antigen (FMP010) based upon the FVO allele will be administered in humans. Therefore, a dose-escalation approach (1/5^th^ dose of 10 micrograms and a full dose of 50 micrograms) will be taken to evaluate safety and reactogenicity in healthy malaria-naïve individuals in the U.S. Safety will be assessed after the first 1/5^th^ dose has been given prior to proceeding to the full dose. Safety of the full dose in adults in the U.S. will be assessed after two vaccinations prior to proceeding to administration of the full dose in malaria-experienced healthy adults in Western Kenya. The candidate vaccine, FMP010/AS01B, consists of the FMP010 antigen formulated with GSK proprietary adjuvant AS01B, the adjuvant currently considered for use in planned pediatric Phase 3 malaria vaccine trials with the pre-erythrocytic malaria candidate vaccine, RTS,S/ASO1B.

Prior experience with MSP1-based vaccines in humans has indicated the requirement for potent adjuvants to elicit sufficiently high antibody titers against epitopes within the carboxy-terminal region of MSP1. Pre-clinical immunogenicity studies in small animals indicate that the FVO allele of the MSP1 antigen is superior to the 3D7 allelic variant in producing functional anti-parasite antibody activity as assessed in the in vitro growth inhibition assay (GIA).

A previous Phase 1/2b pediatric malaria vaccine study conducted in Kenya with the 3D7 allele of MSP1 (FMP1) in the adjuvant AS02A did not adequately demonstrate protective efficacy against clinical disease (manuscript in preparation). However, the 3D7 allele is rare at this location. Most adults in the target study population already have some level of pre-existing immune response to this antigen, therefore data from this population will be required to make an accurate assessment of the immunogenicity of the vaccine.

# Primary Objective: Part B

Assess the safety and reactogenicity of FMP010/AS01B candidate malaria vaccine in healthy, malaria experienced adults in Kenya.

# Secondary Objective: Part B

Measure and compare, by Growth Inhibition Assay (GIA), the functional humoral immune responses induced in malaria experienced adults in Kenya to that induced in healthy malaria-naïve adults from the US by FMP010/AS01B and FMP1/AS02 A.

**Tertiary Objectives: Part B**

Measure by enzyme-linked immunoabsorbent assay (ELISA), the humoral immune responses induced by FMP010/AS01B in healthy malaria experienced adults in Kenya.

**Study design**

| **Part B: RANDOMIZED, BLINDED IN WESTERN KENYA** | | | | | | | |
| --- | --- | --- | --- | --- | --- | --- | --- |
| Group | Location | Study Design | Test Article | Volunteers per group | Dose 1 | Dose 2 | Dose 3 |
| 3 | USAMRU-K, Kenya | Controlled Double Blind | Full dose (50 mcg) of FMP010/AS01B | 20 | **Part B Day 0** | **Part B Day 28** | **Part B Day 56** |
| 4 | USAMRU-K, Kenya | Controlled Double Blind | Rabipur Rabies Vaccine | 10 | **Part B Day 0** | **Part B Day 28** | **Part B Day 56** |

**Part B: Phase 1b Double-blind Controlled at USAMRU-K in Western Kenya**

Healthy, malaria-experienced adults aged 18-50 years

Two groups (20 volunteers in Group 3, 10 volunteers in Group 4; 30 volunteers total)

- Group 3 will receive full dose FMP010 antigen (approximately 50 µg) in 0.5 mL AS01B adjuvant
- Group 4 will receive the licensed rabies vaccine Rabipur® (manufactured in India by Chiron Behring Vaccines Pvt, Ltd) supplied in single dose vials containing lyophilized antigen with 1.0 mL of diluent (sterile water) for injection.

Vaccination schedule of 0, 28 ± 3 days and 56 days ± 3 days

Intramuscular route of administration in the deltoid muscle of the non-dominant arm

**Duration of the study**

Per subject: Approximately seven months from screening to study close-out

**Number of subjects**

30 subjects, 20 subjects in Group 3 (malaria vaccine) and 10 subjects in Group 4 (rabies vaccine))

**Co-primary endpoints**

- Occurrence and intensity of solicited symptoms on day of vaccination plus days 1, 2, 3 and 7 and after each vaccination
- Occurrence and intensity of unsolicited symptoms over a 30-day follow-up period (day of vaccination plus 29 subsequent days) after each vaccination
- Occurrence of serious adverse events (SAEs), defined IAW 21 CFR 312.32(a), during the study period.

**Secondary endpoints**

Functionality of antibodies elicited as measured by percent parasite growth inhibition determined by GIA against FVO and 3D7 strains of *P. falciparum* at specified time points

**Tertiary endpoints**

Titers of anti-FMP010 and anti-FMP1 antibodies as determined by ELISA at specified time points

- To whole MSP1_42_ antigen of 3D7 and FVO allele
- To subunit antigens and selected peptides of MSP1_42_ molecule

# TABLE OF CONTENTS

[protocol Title 1](#__RefHeading___Toc198006295)

[Clinical Investigators 1](#__RefHeading___Toc198006296)

[INVESTIGATORS’ SIGN IN 4](#__RefHeading___Toc198006297)

[SYNOPSIS 5](#__RefHeading___Toc198006298)

[Background: 5](#__RefHeading___Toc198006299)

[Part B: Indication/ Study Population 5](#__RefHeading___Toc198006300)

[Study Rationale 5](#__RefHeading___Toc198006301)

[Primary Objective: Part B 6](#__RefHeading___Toc198006302)

[Secondary Objective: Part B 6](#__RefHeading___Toc198006303)

[TABLE OF CONTENTS 10](#__RefHeading___Toc198006304)

[LIST OF TABLES & FIGURES 13](#__RefHeading___Toc198006305)

[LIST OF ABBREVIATIONS 14](#__RefHeading___Toc198006306)

[GLOSSARY OF TERMS 16](#__RefHeading___Toc198006307)

[1 INTRODUCTION 17](#__RefHeading___Toc198006308)

[**1.1 Medical application and status 17**](#__RefHeading___Toc198006309)

[**1.2 Life cycle of the malaria parasite 17**](#__RefHeading___Toc198006310)

[**1.3 Rationale for Antigen and Adjuvant Selection 17**](#__RefHeading___Toc198006311)

[**1.3.1 FMP010 18**](#__RefHeading___Toc198006312)

[**1.3.2 AS01B 19**](#__RefHeading___Toc198006313)

[**1.3.3 FMP1/AS02A 24**](#__RefHeading___Toc198006314)

[**1.3.4 Rationale for this study 24**](#__RefHeading___Toc198006315)

[2 STUDY OBJECTIVES AND ENDPOINTS 26](#__RefHeading___Toc198006316)

[**2.1 OBJECTIVES 26**](#__RefHeading___Toc198006317)

[Primary Objective 26](#__RefHeading___Toc198006318)

[Secondary Objective 26](#__RefHeading___Toc198006319)

[**2.2 ENDPOINTS 26**](#__RefHeading___Toc198006320)

[3 STUDY DESIGN OVERVIEW 28](#__RefHeading___Toc198006321)

[4 STUDY COHORT 29](#__RefHeading___Toc198006322)

[**4.1 Number of subjects / centers 29**](#__RefHeading___Toc198006323)

[**4.2 Inclusion criteria 29**](#__RefHeading___Toc198006324)

[**4.3 Exclusion criteria for enrollment 29**](#__RefHeading___Toc198006325)

[**4.4 Elimination criteria during the study 31**](#__RefHeading___Toc198006326)

[**4.4.1 Contraindications to vaccination 31**](#__RefHeading___Toc198006327)

[5 CONDUCT OF STUDY 33](#__RefHeading___Toc198006328)

[**5.1 Principal Investigators and Monitors 33**](#__RefHeading___Toc198006329)

[**5.2 Ethics and regulatory considerations 33**](#__RefHeading___Toc198006330)

[**Institutional Review Board/Independent Ethics Committee (IRB/IEC) 33**](#__RefHeading___Toc198006331)

[**Informed consent 33**](#__RefHeading___Toc198006332)

[**5.3 General Study Aspects 34**](#__RefHeading___Toc198006333)

[**Screening 35**](#__RefHeading___Toc198006334)

[**Study Design 36**](#__RefHeading___Toc198006335)

[**5.4 Safety considerations for the trial 37**](#__RefHeading___Toc198006336)

[**Procedure for Evaluating Vaccine Safety in Part A and Progression to Part B 37**](#__RefHeading___Toc198006337)

[**Procedure for Evaluating Vaccine Safety in Part B and Progression to the Next Dose 37**](#__RefHeading___Toc198006338)

[**Holding Rule for Progression to Next Dose in Part B 37**](#__RefHeading___Toc198006339)

[**Stopping Rules 38**](#__RefHeading___Toc198006340)

[**Risks to the Subjects 38**](#__RefHeading___Toc198006341)

[**Risks to Study personnel 39**](#__RefHeading___Toc198006342)

[**5.5 Outline of study procedures 40**](#__RefHeading___Toc198006343)

[**Screening and Vaccination phase 40**](#__RefHeading___Toc198006344)

[**Protocol Deviations 47**](#__RefHeading___Toc198006345)

[**Sample handling and analysis 48**](#__RefHeading___Toc198006346)

[**Serology plan 48**](#__RefHeading___Toc198006347)

[6 STUDY VACCINE AND ADMINISTRATION 49](#__RefHeading___Toc198006348)

[**6.1 Study vaccine 49**](#__RefHeading___Toc198006349)

[**6.1.1 Name and Description of the Investigational Product 49**](#__RefHeading___Toc198006350)

[**6.1.2 Name and Description of the Control Vaccine 49**](#__RefHeading___Toc198006351)

[**6.1.3 Adjuvants 49**](#__RefHeading___Toc198006352)

[**6.2 Dosage and administration 49**](#__RefHeading___Toc198006353)

[**6.2.1 Treatment allocation 50**](#__RefHeading___Toc198006354)

[**6.3 Storage 50**](#__RefHeading___Toc198006355)

[**6.3.1 Packaging: See Appendix E 50**](#__RefHeading___Toc198006356)

[**6.3.2 Accountability: See Appendix E 50**](#__RefHeading___Toc198006357)

[**6.3.3 Replacement of unusable vaccine doses 50**](#__RefHeading___Toc198006358)

[7 Concomitant medication/treatment 52](#__RefHeading___Toc198006359)

[8 ADVERSE EVENTS 53](#__RefHeading___Toc198006360)

[**8.1 Eliciting and documenting adverse events 53**](#__RefHeading___Toc198006361)

[**8.1.1 Adverse event definition 53**](#__RefHeading___Toc198006362)

[**8.1.2 Solicited adverse events 55**](#__RefHeading___Toc198006363)

[**8.1.3 Unsolicited adverse events 55**](#__RefHeading___Toc198006364)

[**8.2 Assessment of intensity 55**](#__RefHeading___Toc198006365)

[**8.3 Assessment of causality 59**](#__RefHeading___Toc198006366)

[**8.4 Following-up of adverse events and assessment of outcome 60**](#__RefHeading___Toc198006367)

[**8.5 Serious adverse events 61**](#__RefHeading___Toc198006368)

[**8.5.1 Definition of a serious adverse event 61**](#__RefHeading___Toc198006369)

[**8.5.2 Reporting serious adverse events 61**](#__RefHeading___Toc198006370)

[**8.5.3 Pregnancy 65**](#__RefHeading___Toc198006371)

[**8.5.4 Treatment of adverse events 66**](#__RefHeading___Toc198006372)

[9 SUBJECT COMPLETION AND DROPOUT 66](#__RefHeading___Toc198006373)

[**9.1 Definition 66**](#__RefHeading___Toc198006374)

[**9.2 Procedures for handling dropouts 66**](#__RefHeading___Toc198006375)

[**9.3 Reasons for dropout 66**](#__RefHeading___Toc198006376)

[10 DATA EVALUATION: CRITERIA FOR EVALUATION OF OBJECTIVES 67](#__RefHeading___Toc198006377)

[**10.1 Study Objectives 67**](#__RefHeading___Toc198006378)

[Primary Objective 67](#__RefHeading___Toc198006379)

[Secondary Objective 67](#__RefHeading___Toc198006380)

[**10.2 Study Endpoints 67**](#__RefHeading___Toc198006381)

[**Exploratory Endpoints 68**](#__RefHeading___Toc198006382)

[**10.3 Study data sets to be evaluated 68**](#__RefHeading___Toc198006383)

[**Estimated Sample Size and Sample Size Justification 69**](#__RefHeading___Toc198006384)

[**10.4 Final Analyses 69**](#__RefHeading___Toc198006385)

[**10.4.1 Analysis of demographics 69**](#__RefHeading___Toc198006386)

[**10.4.2 Analysis of safety 69**](#__RefHeading___Toc198006387)

[Analysis of reactogenicity 69](#__RefHeading___Toc198006388)

[Clinical Laboratory parameters 70](#__RefHeading___Toc198006389)

[**10.4.3 Analysis of immunogenicity 70**](#__RefHeading___Toc198006390)

[Anti-FMP010 antibody responses 70](#__RefHeading___Toc198006391)

[11 ADMINISTRATIVE MATTERS 71](#__RefHeading___Toc198006392)

[12 REFERENCES 72](#__RefHeading___Toc198006393)

[Appendix A: World Medical Association Declaration of Helsinki 76](#__RefHeading___Toc198006394)

[Appendix B: Administrative Matters 80](#__RefHeading___Toc198006395)

[Appendix C: Overview of the Recruitment Plan 84](#__RefHeading___Toc198006396)

[Appendix D: Vaccine Supplies, Packaging and Accountability 85](#__RefHeading___Toc198006398)

[Appendix E: Informed Consent Document 86](#__RefHeading___Toc198006399)

[***Consent for HIV test* 100**](#__RefHeading___Toc198006400)

[Appendix F: List of investigators and study contributors and their roles and responsibilities 103](#__RefHeading___Toc198006401)

**APPENDIX G : AMENDMENT 1, WAS - IS DOCUMENT… 106**

# LIST OF TABLES & FIGURES

TABLE 1: ADVERSE EVENTS FOR PHASE 1/2A RTS,S/AS01B VERSUS RTS,S/AS02A…………………………21

FIGURE1: ADEVERSE EVENTS FOR PHASE 1B RTS,S/AS01B VERSUS RTS,S/AS02A………………………….22

TABLE 2: TIME AND EVENT SCHEDULE FOR FMP010/AS01B PHASE I STUDY ………………………………..44

TABLE 3: TIME AND EVENT SCHEDULE FOR FMP010/AS01B PHASE I STUDY **………………………………..45**

TABLE 4: SOLICITED LOCAL AND GENERAL ADVERSE EVENTS ……………………………………………….53

TABLE 5: INTENSITY GRADING OF LOCAL SOLICITED ADVERSE EVENTS …………………………………...54

TABLE 6: INTENSITY GRADING OF GENERAL SOLICITED ADVERSE EVENTS……………………………….54

TABLE 7: INTENSITY GRADINGS ……………………………………………………………………………………..55

TABLE 8: INTENSITY GRADING OF LABORATORY ABNORMALITIES (PART B)……………………………...56

TABLE 9: INTENSITY GRADING OF LABORATORY ABNORMALITIES (PART B)……………………………...57

# LIST OF ABBREVIATIONS

3-D-MPL 3-deacylated Monophosphoryl Lipid A

AE Adverse Event

ALT Alanine aminotransferase

AP Alkaline phosphatase

AS01B GSK’s proprietary adjuvant system, 1B

AS02A GSK’s proprietary adjuvant system, 2A

AST Aspartate aminotransferase

BPR Batch Production Record

BUN Blood urea nitrogen

CBC Complete blood count

CFR Code of Federal Regulations

CMI Cell mediated immunity

CRF Case Report Form

CTC Clinical Trials Center

DHSP Division of Human Subjects Protection, WRAIR

ELISA Enzyme linked immunosorbent assay

ERC Ethical Review Committee

FMP010 Falciparum Malaria Protein 010 (MSP1_42_ (FVO))

FMP1 Falciparum Malaria Protein 1 (MSP1_42_ (3D7))

F/u follow-up

GCP Good Clinical Practice

GIA Growth Inhibition Assay

GMP Good Manufacturing Practice

GMT Geometric Mean Titer

GSK Bio GSK Biologicals

HBsAg Hepatitis B surface antigen

HCV Hepatitis C Virus

HIV-1 Human Immunodeficiency Virus-1

HRP-2 Histidine Rich Protein 2

HRPO Human Research Protections Office

HSRRB Human Subjects Research Review Board

IAW in accordance with

ICH International Conference on Harmonization

IFA Indirect Immunofluorescence Assay

IFNγ Interferon gamma

IM Intramuscular

IND Investigational New Drug

IRB Institutional Review Board

IU/l International units per liter

LDH Lactate dehydrogenase

mmol/l Millimoles per liter

MPL® 3-deacylated monophosphoryl lipid A

MSP1 Merozoite Surface Protein 1

MRMC Medical Research and Material Command

*P. falciparum* *Plasmodium falciparum*

PBMC Peripheral blood mononuclear cells

PCV Packed cell volume (hematocrit values)

PFS Pre-filled syringe

PI Principal Investigator

PIA Processing Inhibition Assay

pLDH Plasmodium lactate dehydrogenase

QS-21 *Quillaja saponaria* 21 (saponin derivative)

SAE Serious Adverse Event

SOP Standard Operating Procedure

USAID U.S. Agency for International Development

USAMMDA US Army Medical Material Development Activity

USAMRU-K US Army Medical Research Unit - Kenya

Vacc Vaccination

WHO World Health Organization

WRAIR Walter Reed Army Institute of Research

WRAIR IRB Walter Reed Army institute of Research Institutional Review Board

WRAMC Walter Reed Army Medical Center

# GLOSSARY OF TERMS

| **Volunteers(s)** | Term used throughout the protocol to denote the enrolled individual(s). |
| --- | --- |
| **Local Safety Monitor in Western Kenya** | An experienced physician based in-country who will monitor the clinical safety of the study and its volunteers. |
| **Study Monitor** | An individual who is responsible for assuring proper conduct of a clinical study. |
| **Safety Monitoring Committee** | A group of three independent clinicians assembled to review and evaluate safety data from the study and make necessary recommendations for the progression of the trial at specified time points |
| **Eligible** | Qualified for enrollment into the study based upon strict adherence to inclusion/exclusion criteria. |
| **Evaluable** | Meeting all eligibility criteria, complying with the procedures defined in the protocol, and, therefore, included in analysis (see Section 10 for details on criteria for evaluability). |
| **Protocol amendment** | Any change in a clinical protocol that affects the safety of subjects, the scope, design, assessments or scientific validity of the clinical investigation, e.g., dose change, duration of treatment, number of subjects, control group(s), the assessments. |

# INTRODUCTION

## Medical application and status

Resurgent malaria is a major worldwide problem. Transmission occurs throughout tropical Africa, Asia, Oceania, and Latin America. It has been reported that malaria is responsible for 300-500 million clinical cases annually, and between one and three million deaths, mostly in children (4). The loss of effective anti-malaria control programs and emerging drug resistant malaria make control by traditional means difficult. If available, an effective malaria vaccine would be an important and useful intervention. *Plasmodium falciparum*, the human malaria parasite causing most of the mortality and morbidity, is endemic to many areas of the world. Malaria can cause significant morbidity and mortality in immunologically naïve individuals such as American service personnel as well as endemic populations, and a vaccine to prevent this disease or lessen the severity of the illness would be widely beneficial.

## Life cycle of the malaria parasite

The malaria parasite has a complex life cycle. The bite of an infectious mosquito transmits the sporozoite form of the parasite to humans. These sporozoites travel through the bloodstream and ultimately invade liver cells where they multiply asexually as exoerythrocytic stage parasites. The exoerythrocytic forms of *P. falciparum* mature in five to seven days releasing thousands of tissue merozoites that invade erythrocytes and initiate the erythrocytic phase of the infection. At this time, free merozoites invade erythrocytes, undergo asexual maturation, and ultimately rupture the erythrocyte releasing new merozoites. It is this phase, characterized by cyclic destruction of erythrocytes that results in the clinical disease known as malaria. A small number of the invading merozoites do not multiply, but instead differentiate into sexual forms known as gametocytes. When ingested by a mosquito, male and female gametocytes can unite to form a zygote, which then matures to release sporozoites that subsequently migrate to the mosquito salivary glands. These sporozoites are available to infect the next person bitten by the mosquito, completing the life cycle.

## Rationale for Antigen and Adjuvant Selection

WRAIR, in collaboration with USAID and GSK, hopes to develop a malaria vaccine that targets the asexual blood stage of *P. falciparum* to lessen the morbidity resulting from blood stage malaria infection. Several antigens from the asexual blood stage of the parasite *P. falciparum* are being considered as vaccine candidates.

### FMP010

The FMP010 antigen is the C-terminal 42-kDa portion of the Merozoite Surface Protein 1 (MSP1) from the FVO strain of *Plasmodium falciparum* expressed in and purified from *Escherichia coli*. FMP010 is a single polypeptide encoding 371 amino acids, consisting of 16 non-MSP1 amino acids fused to the N-terminus of a 355 amino acid C-terminal MSP1 (representing bp 3834-4898 from the wild type MSP1 FVO sequence, Genbank Accession number X03371, encoding amino acids 1333-1688).

The major merozoite surface protein-1 (MSP1) is among the leading erythrocytic stage candidates for inclusion in a multi-stage malaria vaccine. In *P. falciparum*, this protein is between 185–200 kDa in size by gel electrophoretic analysis and is attached at its C-terminus to the parasite plasma membrane via a glycosylphosphatidylinositol anchor (5). MSP1 is present on the merozoite’s surface as a complex of fragments (6, 7) derived by proteolytic processing of the precursor (6, 8, 9,). During erythrocytic invasion by merozoites, MSP1_42_, which is the major fragment from the C-terminus, is processed secondarily, producing a 33kDa fragment (MSP1_33_) from the N-terminus and a 19kDa fragment from the C-terminus (MSP1_19_). MSP1_19_ is highly structured, folding into two EGF-like domains known as EGF-like domain 1 and EGF-like domain 2 (10). MSP1_19_ remains attached to the merozoite surface and is present on ring forms in newly invaded erythrocytes (11, 12). Sequence variation among MSP1_42_ molecules from different *P. falciparum* strains is primarily dimorphic (13).

Both MSP1_42_ and MSP1_19_ are established targets of protective immunity in animal models, and in the case of the rodent malaria model, *P. yoelii*, the protection observed is strain specific (14). Additional evidence for the importance of this region of MSP1 in immunity includes the observation that MSP1_19_-specific monoclonal antibodies (mAb) inhibit *P. falciparum* growth *in vitro* (15, 16), and passively protect mice against *P. yoelii* malaria infection (17, 18). It has also been shown that mAbs that inhibit *P. falciparum* growth *in vitro,* also inhibit secondary processing of MSP1_42_ (19) and bind to conformational epitopes that are lost when cystines are reduced. MSP1_19_-specific Ab have proven to be the major contributors to the invasion-inhibitory response among humans that are immune to malaria (20) and double domain-specific p19 as well as EGF domain-2 specific antibodies affinity purified from immune human sera prevent parasite invasion *in vitro* (21). Several studies have shown that rabbit anti-sera raised against recombinant MSP1_42_ inhibit *P. falciparum* growth *in vitro* (22, 23, 24).

Active vaccination of *Aotus nancymai* monkeys with *E. coli*- (24) and baculovirus-expressed (25, 26, 27) recombinant *P. falciparum* MSP1_42_ induces a significant homologous protective effect against infection by erythrocytic stage FVO strain *P. falciparum*. MSP1_42_ purified from transgenic mouse milk also induces protection, but this result depends on removing two N-glycosylation sites from the protein (26). Since *P. falciparum* does not N-glycosylate proteins, expression strategies that avoid glycosylation are most suited for malaria vaccines based on MSP1_42_.

Our objective was to manufacture clinical grade *E. coli*-expressed recombinant MSP1_42_ (FVO) antigen that preserved various elements of correct antigen structure, as evidenced by binding functional mAbs specific for conformation-dependent epitopes. Furthermore, we have shown that this antigen is capable of inducing functional immunity by using growth inhibition assays to evaluate vaccinated rabbit sera or by challenging vaccinated *Aotus nancymai* monkeys with the highly virulent FVO strain of *P. falciparum*, which is homologous to the vaccine (28).

### AS01B

#### Pre-clinical Evaluation

The pre-clinical evaluation of new adjuvant formulations has led to the selection of AS01B as a candidate possibly capable of prolonging persistence of the immune response. The GSK proprietary adjuvant system 1B (AS01B) is composed of the immunostimulants Stimulon® QS21 (a triterpene glycoside purified from the bark of *Quillaja saponaria*) and MPL® with liposomes. Preclinical studies in rhesus monkeys show that the RTS,S antigen formulated with the AS01B adjuvant (RTS,S/AS01B) is safe and induces superior T-cell-specific and interferon gamma (IFN-gamma) responses to RTS,S/AS02A in addition to generating good humoral responses (29). In toxicology studies in rabbits, the adjuvant was shown to be safe and well tolerated (Spring Valley Laboratories, Study No. SVT05-09). High titer anti-MSP1_42_-specific antibodies are induced to a greater degree in rabbits immunized with FMP010/AS01B than FMP010/AS02A, and these antibody responses result in greater parasite inhibitory activity in a growth inhibition functional assay (personal communication E. Angov).

#### Clinical Evaluation

The initial clinical experience with AS01B comes from a study of AS01B-adjuvanted hepatitis B vaccine (personal communication, P. Vandepapieliere). In this double blind Phase I/II immunogenicity and safety study in adults, hepatitis B surface antigen (HBsAg) was formulated with AS01B. The HBsAg used in this study is encoded by the same gene as that used to express HBsAg in the RTS,S antigen (which contains both hepatitis and malaria moieties), and the commercially available hepatitis B vaccine (Engerix-B). No SAEs deemed causally related by the investigator to vaccination and no clinically significant abnormal hematological or biochemical laboratory test results have been reported. Most solicited symptoms reported after vaccination with HBsAg/AS01B were not severe in intensity and resolved in the course of the seven-day follow-up period after vaccination. All unsolicited symptoms that were causally related to HBsAg/AS01B vaccination and of intensity Grade 3 (symptoms that prevented normal everyday activity) were local (injection site) adverse events and resolved within two days. In conclusion, HBsAg/AS01B was safe and induced an acceptable amount of reactogenicity.

In the evaluation of immunogenicity, HBsAg/AS01B induced a high and persistent humoral immune response as shown by the presence of seroprotective levels of HBs antibodies 40 weeks after the second dose of vaccine and dramatic boosting of geometric mean titers of anti-HBs antibodies to levels above 900,000 mIU/mL after Dose 3. The cell-mediated immune (CMI) responses to vaccination were characterized by a HBs-specific cytotoxic response, marked lymphoproliferation and IFN-gamma secretion but low interleukin-5 secretion. These CMI results are indicative of a Th1-like immune response and confirm the immunogenicity profile established for the AS01B adjuvant in preclinical studies with RTS,S/AS01B.

The first clinical study of the malaria vaccine RTS,S adjuvanted with AS01B (MAL-027) was initiated in healthy, malaria-naive adults, aged 18 to 50 years at WRAIR, Silver Spring, MD, USA. This study was a double-blind, randomized, controlled challenge, Phase 1/2a study involving two sequential cohorts, Cohort 1 and Cohort 2. Volunteers received three doses of either RTS,S/AS01B or RTS,S/AS02A vaccine at months 0, 1, 2, followed by a challenge with *P. falciparum*-infected mosquitoes post Dose 3. Each of the cohorts enrolled approximately 50 subjects, randomly allocated to receive RTS,S/AS01B or RTS,S/AS02A on a 1:1 ratio. The overall incidence of solicited and unsolicited AEs was similar in the RTS,S/AS01B and RTS,S/AS02A groups and is presented in Table 1 (personal communication K. Kester). Pain was the most frequently reported solicited local AE in both the RTS,S/AS01B and RTS,S/AS02A groups, occurring with a similar frequency in both vaccine groups (following 81.0% and 82.1% of doses, respectively). Grade 3 local events tended to be as frequent in subjects receiving RTS,S/AS01B as those receiving RTS,S/AS02A with the exception of pain which was observed to be more frequent in the RTS,S/AS02A group (after 9.0% of doses) than in the RTS,S/AS01B group (after 2.8% of doses). For solicited general adverse events, fatigue, malaise and headache were the most common events recorded and the incidence of all three was similar in both vaccine groups. Overall, Grade 3 events were infrequent, occurring < 4.2% doses administered. Unsolicited events considered to be related to the vaccine occurred at a similar rate in both groups (19.7% in the RTS,S/AS01B group and 15.2% in the RTS,S/AS02A group). Three SAEs were reported by three subjects and none were considered to have a causal relationship to study drug. One subject was withdrawn from the study after developing a transient urticarial reaction after receipt of RTS,S/AS01B.

Table 1: Adverse Events for Phase 1/2a RTS,S/AS01B versus RTS,S/AS02A

Frequency of solicited symptoms per dose during the seven-day follow-up period (Total Cohort, Malaria-027)

| **Incidence per dose of solicited local symptoms reported during the 7-day follow-up period**  **(Total Cohort- pooled Cohorts 1 and 2)** | | | | | | | | | | | | | | | | | | | | | | |
| --- | --- | --- | --- | --- | --- | --- | --- | --- | --- | --- | --- | --- | --- | --- | --- | --- | --- | --- | --- | --- | --- | --- |
| **Group** | |  | **RTS,S/AS02A** | | | | | | | | | | **RTS,S/AS01B** | | | | | | | | | |
|  | |  | **N** | | **n** | | **%** | | **95% CI** | | | | **N** | | **n** | | **%** | | **95% CI** | | | |
| Pain | | Any | 145 | | 119 | | 82.1 | | 74.8 | | 87.9 | | 142 | | 115 | | 81.0 | | 73.6 | | 87.1 | |
|  | | Grade 3 | 145 | | 13 | | 9.0 | | 4.9 | | 14.8 | | 142 | | 4 | | 2.8 | | 0.8 | | 7.1 | |
| Redness | | Any | 145 | | 36 | | 24.8 | | 18.0 | | 32.7 | | 142 | | 35 | | 24.6 | | 17.8 | | 32.6 | |
|  | | Grade 3 | 145 | | 4 | | 2.8 | | 0.8 | | 6.9 | | 142 | | 7 | | 4.9 | | 2.0 | | 9.9 | |
| Swelling | | Any | 145 | | 31 | | 21.4 | | 15.0 | | 29.0 | | 142 | | 24 | | 16.9 | | 11.1 | | 24.1 | |
|  | | Grade 3 | 145 | | 2 | | 1.4 | | 0.2 | | 4.9 | | 142 | | 3 | | 2.1 | | 0.4 | | 6.0 | |
| **Incidence per dose of solicited general symptoms reported during the 7-day follow-up period (Total Cohort – Pooled Cohorts 1 and 2)** | | | | | | | | | | | | | | | | | | | | | | |
| **Group** |  | | | **RTS,S/AS02A** | | | | | | | | | | **RTS,S/AS01B** | | | | | | | | |
|  |  | | | **N** | | **n** | | **%** | | **95% CI** | | | | **N** | | **n** | | **%** | | **95% CI** | | |
| Fatigue | Any | | | 145 | | 64 | | 44.1 | | 35.9 | | 52.6 | | 142 | | 51 | | 35.9 | | 28.0 | | 44.4 |
|  | Grade 3 | | | 145 | | 4 | | 2.8 | | 0.8 | | 6.9 | | 142 | | 7 | | 4.9 | | 2.0 | | 9.9 |
|  | Grade 3/related | | | 145 | | 3 | | 2.1 | | 0.4 | | 5.9 | | 142 | | 6 | | 4.2 | | 1.6 | | 9.0 |
| Headache | Any | | | 145 | | 48 | | 33.1 | | 25.5 | | 41.4 | | 142 | | 50 | | 35.2 | | 27.4 | | 43.7 |
|  | Grade 3 | | | 145 | | 1 | | 0.7 | | 0.0 | | 3.8 | | 142 | | 5 | | 3.5 | | 1.2 | | 8.0 |
|  | Grade 3/related | | | 145 | | 0 | | 0.0 | | 0.0 | | 2.5 | | 142 | | 4 | | 2.8 | | 0.8 | | 7.1 |
| Malaise | Any | | | 145 | | 41 | | 28.3 | | 21.1 | | 36.3 | | 142 | | 35 | | 24.6 | | 17.8 | | 32.6 |
|  | Grade 3 | | | 145 | | 5 | | 3.4 | | 1.1 | | 7.9 | | 142 | | 2 | | 1.4 | | 0.2 | | 5.0 |
|  | Grade 3/related | | | 145 | | 4 | | 2.8 | | 0.8 | | 6.9 | | 142 | | 2 | | 1.4 | | 0.2 | | 5.0 |
| Myalgia^1^ | Any | | | 145 | | 34 | | 23.4 | | 16.8 | | 31.2 | | 142 | | 29 | | 20.4 | | 14.1 | | 28.0 |
|  | Grade 3 | | | 145 | | 0 | | 0.0 | | 0.0 | | 2.5 | | 142 | | 2 | | 1.4 | | 0.2 | | 5.0 |
|  | Grade 3/related | | | 145 | | 0 | | 0.0 | | 0.0 | | 2.5 | | 142 | | 2 | | 1.4 | | 0.2 | | 5.0 |
| Gastrointestinal | Any | | | 145 | | 20 | | 13.8 | | 8.6 | | 20.5 | | 142 | | 24 | | 16.9 | | 11.1 | | 24.1 |
|  | Grade 3 | | | 145 | | 3 | | 2.1 | | 0.4 | | 5.9 | | 142 | | 1 | | 0.7 | | 0.0 | | 3.9 |
|  | Grade 3/related | | | 145 | | 1 | | 0.7 | | 0.0 | | 3.8 | | 142 | | 1 | | 0.7 | | 0.0 | | 3.9 |
| Arthralgia^2^ | Any | | | 145 | | 19 | | 13.1 | | 8.1 | | 19.7 | | 142 | | 24 | | 16.9 | | 11.1 | | 24.1 |
|  | Grade 3 | | | 145 | | 0 | | 0.0 | | 0.0 | | 2.5 | | 142 | | 2 | | 1.4 | | 0.2 | | 5.0 |
|  | Grade 3/related | | | 145 | | 0 | | 0.0 | | 0.0 | | 2.5 | | 142 | | 1 | | 0.7 | | 0.0 | | 3.9 |
| Fever^1^ | Any | | | 145 | | 17 | | 11.7 | | 7.0 | | 18.1 | | 142 | | 21 | | 14.8 | | 9.4 | | 21.7 |
| 1. No grade 3 events reported 2. No grade 3 related events reported   N= number of administered doses; n/%= number/percentage of doses followed by at least one type of symptom  95%CI= Exact 95% confidence interval; Lower/Upper limits | | | | | | | | | | | | | | | | | | | | | | |

A Phase 2 study comparing RTS,S/AS01B and RTS,S/AS02A to rabies vaccine in 255 adults in western Kenya has recently been completed. Both candidate malaria vaccines were safe and well tolerated. There were similar rates of general symptoms in all three groups. There were no Grade 3 solicited symptoms or general reactions related to vaccination. In recipients of both RTS,S/AS01B and RTS,S/AS02A there was a trend towards decreased local reactogenicity with subsequent vaccine doses.

Figure 1: Adverse Events for Phase 1b RTS,S/AS01B versus RTS,S/AS02A

Percentage of subjects reporting local and general adverse events within seven days of each dose (Total Cohort)

Unsolicited adverse events were reported, within 30 days of vaccination, by 96% of volunteers. Sixty five volunteers had elevated bilirubin during the course of the study (22 recipients of RTS,S/AS01B, 19 recipients of RTS,S/AS02A, 24 recipients of control). Eleven volunteers had elevations in ALT (1 recipient of RTS,S/AS01B, 7 recipients of RTS,S/AS02A, 3 recipients of control). All instances were mild and transient. Bilirubin elevations were not associated with any other liver test abnormalities.

Nineteen volunteers had mild anemia (10 recipients of RTS,S/AS01B, 3 of RTS,S/AS02A, and 6 of control). One female subject had a mild decrease in WBC count that normalized without intervention. Two subjects had mild, transient lymphopenia.

Serious adverse events resulting in hospitalization were reported by four subjects – two recipients of RTS,S/AS01B and two recipients of rabies vaccine. None of the SAEs were related to vaccination.

Both the AS01B and the AS02A vaccines were highly immunogenic for anti-CS antibodies with RTS,S/AS01B producing a significantly higher response. The improved antibody response of RTS,S/AS01B was maintained up to the end of the period of efficacy surveillance (Day 180).

Pooled data yielded an unadjusted vaccine efficacy of 30.9%. There was no difference in efficacy between the two vaccine candidates.

#### FMP010/AS01B (Preclinical)

Data from a repeat dose and acute GLP toxicology study (Study No. SVT05-09, Spring Valley Laboratories, MD) in New Zealand White rabbits indicate that the FMP010/AS01B formulation is safe and immunogenic.

Four groups of rabbits (Groups were Saline alone, AS01B alone, FMP010/Saline and FMP010/AS01B with 10 males and 10 females per group) were evaluated for clinical and pathological evidence of toxicity to FMP010 when administered in combination with AS01B or normal saline. No clinical indications of toxicity were manifest during the course of the study from any of the parameters that were evaluated (BB-IND 13638, Section 5). Thus, the FMP010 when administered alone or formulated with the adjuvant material AS01B was demonstrated to be generally safe and well tolerated.

In this study, the antibody levels in animals immunized with FMP010/AS01B were significantly higher than the antibody levels for any group including for FMP010/Saline at all time points (p<0.001). Even after four vaccinations, the FMP010/Saline group had significantly lower antibody titers than FMP010/AS01B after three vaccinations (third vaccination: 43, 000 versus 191,000 versus fourth vaccination: 39,000 versus 334,000, respectively) (p<0.001).

In a second immunogenicity study performed in rabbits, Spring Valley Laboratories Study ID SVP05-0147, eight New Zealand white rabbits per group were vaccinated at three-week intervals, four times with 50 μg doses of FMP010/AS01B and a comparator MSP1_42_ vaccine, FMP1/AS01B (*P. falciparum* MSP1_42_ 3D7 allele). Sera were collected two weeks following the third (day 56) vaccination and evaluated by ELISA against homologous and heterologous fragments of MSP1_42_. ELISA antibody titers measured against the FMP010 homologous MSP1_42_ FVO plate antigen for the FMP010/AS01B group were significantly higher then for FMP1/AS01B (ELISA titer is reported as the reciprocal dilution necessary to give an OD=1, FMP010/AS01B and FMP1/AS01B with 188,590 and 63,640 ELISA units, respectively). The FMP010/AS01B heterologous response (i.e. against the MSP1_42_ 3D7 allele antigen) was also significantly higher from that induced by FMP1/AS01B (103,571 and 78,573, respectively). Therefore, FMP010/AS01B is highly immunogenic against both homologous and heterologous strain parasites.

When sera from these rabbits were evaluated for their *in vitro* parasite growth inhibitory activities by measuring parasite lactate dehydrogenase levels, pLDH, we observed a statistically significant difference between FMP010/AS01B and FMP1/AS01B induced responses against their respective homologous parasite strains (i.e. FVO and 3D7, respectively), p = 0.06, one-way ANOVA, mean inhibition at 20% serum is 5% for FMP1 and 21% for FMP010.

### FMP1/AS02A

The FMP1 antigen has been under clinical development at WRAIR (30) for over 10 years with accumulated safety and reactogenicity data from two trials conducted at WRAIR and four trials in endemic areas in Mali and Kenya. A Phase 1 dose-escalation study of FMP1/AS02A in 15 malaria-naïve adults conducted at WRAIR in 2001 demonstrated all dose levels to be safe and well tolerated, with no grade 3 adverse reactions and no serious adverse events (31). The vaccine was immunogenic with seroconversion in all vaccinees after the second vaccination. The antibodies induced by this vaccine also demonstrated homologous GIA activity of approximately 15% in 2 of 5 volunteers receiving 50 µg of FMP1 in ASO2A, while there was no demonstrable activity against heterologous (FVO) parasites. Also seen was circumferential staining of fixed schizonts by IFA. A Phase 1/2a study conducted at WRAIR with FMP1/AS02A and RTS,S/AS02A demonstrated that FMP1/AS02A was safe and immunogenic, although it did not confer protection (personal communication J. Cummings).

Of Phase 1 studies conducted in malaria-endemic areas, the first was performed in forty adult participants in Kombewa, Kenya in 2002 comparing FMP1/AS02A to rabies vaccine (32). The most common local side effect related to vaccination with FMP1 was pain which resolved within 48 hrs. No Grade 3 systemic reactions and no vaccine-related vaccine SAE occurred in either group. In adults in Mali, a 50 μg dose of FMP1 in AS02A was safe and well-tolerated with Grade 3 swelling being the most common local side effect as compared to rabies vaccine (33). This swelling lasted less than 48 hours and often was unnoticed by the volunteers.

In Kombewa, Kenya, a Phase I dose-escalation trial of FMP1/AS02A and a rabies comparator vaccine *Imovax*® in children 12-47 months of age was completed in 2003-2004 (33). Safety data showed that the most common solicited adverse events were pain and swelling at the injection site occurring in greater frequency in children who received FMP1/AS02A. These local symptoms had the highest incidence after the first vaccination and resolved within 48-72 hours. Grade 3 local symptoms occurred in less than 10% of children in all dose cohorts. The commonest general symptoms were fever and irritability and these also resolved within 24-48 hours.

### Rationale for this study

The approach to the development of an asexual blood stage vaccine against P. falciparum malaria based upon the MSP1 antigen from the surface of the merozoite is founded on the hypothesis that such a vaccine will elicit strong neutralizing humoral immune responses directed against epitopes located within the 42-kDa carboxy-terminal region of the MSP1 molecule. The main purpose of such a response is to decrease the load of infectious merozoites invading erythrocytes. Indeed, the data available today in the literature suggest these antibody responses targeting the surface of infectious blood stage merozoites are important components of a protective immune response, and that the MSP1 antigen is one of the targets of this response. Ideally, the immune responses should be recalled upon revaccination as well as upon subsequent natural infections. The vaccine should therefore be capable of inducing appropriate subsets of T-helper – memory cells, responding to T-epitopes preferably derived from the MSP1 protein itself.

Prior experience with MSP1-based vaccines in humans has indicated the requirement for sufficiently high antibody titers against epitopes within the carboxy-terminal region of MSP1. In murine and non-human primate models, MSP1-based immunity is strain specific (28). Therefore, it appears that it may be critical to select the most immunogenic allele or a second allele of the MSP1 antigen for inclusion in a malaria vaccine. In addition, molecular typing of P. falciparum parasites from East and West Africa indicate that >90% of the dominant circulating alleles are QKNG and EKNG, and therefore a vaccine that is based on these two allotypes such as the proposed FMP010 stands a better chance of success. This is the compelling justification to proceed with Phase 1a/b clinical testing of the FVO allele of MSP1_42_ (FMP010) proposed in this study (personal communication, CF Ockenhouse).

Given the improved preclinical immunogenicity observed with FMP010, the higher prevalence of the FVO allele circulating in endemic areas, and the improved cell-mediated immune response generated by the adjuvant AS01B (without loss of humoral responses), the opportunity for FMP010/AS01B to provide needed protection against malaria necessitates further evaluation of this allele as a vaccine candidate in both a malaria-naïve and malaria-experienced population. This study is an initial dose-escalation evaluation of the safety and reactogenicity of FMP010/ AS01B at 10 µg and 50 µg of antigen in 0.5 mL of adjuvant in a staggered schedule to permit assessment of safety data at appointed intervals before proceeding to additional vaccinations.

This study will also evaluate immunogenicity of this FMP010/AS01B, and this information will be essential to decision-making policies for consideration of efficacy testing of this candidate. A recently completed Phase 1/2b pediatric vaccine trial with FMP1/AS02A did not appear to demonstrate protective efficacy against malaria in an area of Kenya with multiple MSP-1 alleles (personal communication B. Ogutu).

This Phase I clinical study will evaluate the safety, reactogenicity and immunogenicity profiles of FMP010, which is based on the FVO allele of MSP1, formulated with AS01B after intramuscular administration according to a 0-, 1-, and 2-month schedule. Humoral immune responses to MSP1, considered critical for inhibition of parasite growth, will be evaluated at specific time points during the study and compared to humoral immune responses from a previous vaccine trial evaluating FMP1/AS02A in order to evaluate the potential for progression of this vaccine candidate.

# STUDY OBJECTIVES AND ENDPOINTS

## OBJECTIVES

# Primary Objective

Assess the safety and reactogenicity of FMP010/AS01B candidate malaria vaccine in healthy malaria-naïve adults from the US and malaria experienced adults in Kenya.

# Secondary Objective

Measure and compare, by Growth Inhibition Assay (GIA), the functional humoral immune responses induced in malaria experienced adults in Kenya to that induced in healthy malaria-naïve adults from the US.

**Tertiary Objectives**

Measure and compare, by enzyme-linked immunoabsorbent assay (ELISA), the humoral immune responses induced by vaccine candidate FMP010/AS01B in malaria experienced adults in Kenya to those induced in healthy malaria-naïve adults from the US.

## ENDPOINTS

**Co-primary endpoints**

- Occurrence and intensity of solicited symptoms on day of vaccination plus days 1, 2, 3 and 7 after each vaccination
- Occurrence and intensity of unsolicited symptoms over a 30-day follow-up period (day of vaccination plus 29 subsequent days) after each vaccination
- Occurrence of serious adverse events (SAEs), defined IAW 21 CFR 312.32(a), during the study period.

**Secondary endpoints**

Functionality of antibodies elicited as measured by percent parasite growth inhibition determined by GIA against the FVO and 3D7 strains of *P.* falciparum at specified time points

**Tertiary endpoints**

Titers of anti-FMP010 and anti-FMP1 antibodies as determined by ELISA at specified time points

- To whole MSP1_42_ 3D7 and FVO antigens

● To subunit antigens and selected peptides of MSP1_42_ molecule

**Exploratory Endpoints**

These assays may provide additional information on the immunogenicity of the vaccine candidate FMP010/AS01B. The assays will be completed at USAMRU-K if, after performing endpoint assays, sufficient sample remains and study priorities permit:

- Detection of *P. falciparum* antigens by HRP-2 and pLDH ELISAs
- Titers of anti-FMP010 and FMP1 antibodies as determined by bead-based flow cytometric assays (*Luminex™*) at specified time points
- Anti-merozoite immunofluorescence (IFA) assays to assess ability of antibodies to recognize MSP1 molecule on infected erythrocytes
- Processing inhibition assay (PIA) to assess ability of antibodies to inhibit processing of MSP1_42_ molecule to MSP1_33_ and MSP1_19_
- Cytokine bead-based flow cytometric assay (*Luminex*) to assess cytokine production in responses to vaccine administration
- Assays such as ELISPOT and intracellular cytokine staining to assess cellular immune responses

# STUDY DESIGN OVERVIEW

| **Part B: CONTROLLED DOUBLE BLIND IN WESTERN KENYA** | | | | | | | |
| --- | --- | --- | --- | --- | --- | --- | --- |
| Group | Location | Study Design | Test Article | Volunteers per group | Dose 1 | Dose 2 | Dose 3 |
| 3 | USAMRU-K, Kenya | Controlled Double Blind | Full dose (50 mcg) of FMP010/AS01B | 20 | **Part B Day 0** | **Part B Day 28** | **Part B Day 56** |
| 4 | USAMRU-K, Kenya | Controlled Double Blind | Rabipur® Rabies Vaccine | 10 | **Part B Day 0** | **Part B Day 28** | **Part B Day 56** |

**Study Initiation**

Vaccination and review of the safety data for Groups 1 and 2 (Part A in the USA) will occur before vaccination can begin in Part B (Kisumu, Kenya). Safety data for Groups 1 and 2 (Part A in the USA) will be collected and submitted to a Safety Monitoring Committee (SMC) for recommendations about progression to Part B in Kisumu, Kenya. If progression to Part B is recommended the safety data will be presented to the Medical Monitor for Part B and the KEMRI Ethical Review Committee for evaluation and recommendation to initiate Part B of the study.

Duration of the study, per subject is approximately seven months (screening, enrollment, vaccination, and follow-up). All subjects will be followed for safety for a period that extends to two months post Dose 3. Any data collected after the final analysis will be reported in an annex report. Data collection will be by conventional CRF.

**Part B: Phase 1b Double-blind Controlled at USAMRU-K in Western Kenya**

- Healthy, malaria-experienced adults aged 18-50 years
- Two groups (20 volunteers in Group3, 10 volunteers in Group 4; 30 volunteers total)
  - Group3 will receive full dose FMP010 antigen (approximately 50 µg) in 0.5 mL AS01B adjuvant
  - Group 4 will receive full dose rabies vaccine (Rabipur®)
- Vaccination schedule of 0, 28 ± 3days and 56 days ± 3 days
- Intramuscular route of administration in the deltoid muscle of the non-dominant arm

# STUDY COHORT

## Number of subjects / centers

**USAMRU-K Phase**

Up to 30 healthy, malaria-exposed adults aged 18-50 years will be enrolled in Part B of the study. Part B will be performed at Muriithi-Wellde Clinical Research Center (CTC), Western Kenya. The population of Kombewa Division is primarily Luo. The majority of the population speaks both Dholuo and Kiswahili.

The sample size for both Parts was chosen to assess the first-in-human experimental vaccine, FMP010/AS01B, in small numbers of volunteers and is consistent in size with a Phase 1 study of FMP1/AS02A (3D7 allele) carried out previously at WRAIR.

## Inclusion criteria

- A male or non-lactating female 18 to 50 years of age (inclusive) at the time of screening
- Free of significant health problems as established by medical history and clinical examination before entering into the study
- Available to participate for duration of study (approximately seven months)

If the subject is female, she must be of non-childbearing potential (either surgically sterilized or one year post-menopausal) or, if of childbearing potential, she must have a negative pregnancy test at the time of vaccination, be capable of preventing pregnancy for at least one month prior to determination of eligibility (to include abstinence or contraceptives (for example intrauterine contraceptive device; oral contraceptives; Norplant® or Depo-Provera® ), and must agree to continue such precautions for two months after completion of the vaccination series.

- Written informed consent must be obtained from the subject before screening procedures.
- After the group briefing, volunteers will review the protocol, one-on-one, with a member of the study staff to ensure they understand the study and consent document. Clinical points of the study will be reviewed again during the screening evaluation.

## Exclusion criteria for enrollment

The following criteria will be checked at the time of study entry. If any apply at the time of study entry, the subject must not be included in the study:

- Prior receipt of any investigational malaria vaccine
- Prior receipt of a vaccine containing either QS-21, MPL or AS02A or AS01B
- Use of any investigational or non-registered drug or vaccine other than the study vaccine within 30 days preceding the first dose of study vaccine, or planned use during the study period
- Administration of chronic (defined as more than 14 days) immunosuppressants or other immune modifying drugs within six months of vaccination. For corticosteroids, this is defined as prednisone, or equivalent, 0.5 mg/kg/day. Inhaled and topical steroids are allowed.
- Planned administration of a vaccine not foreseen by the study protocol within 30 days of the first dose of the study vaccine.
- Any confirmed or suspected immunosuppressive or immunodeficient condition, including human immunodeficiency virus (HIV) infection
- A family history of congenital or hereditary immunodeficiency
- Chronic or active neurologic disease including seizure disorder
- History of splenectomy
- Acute or chronic, clinically significant pulmonary, cardiovascular, hepatic or renal functional abnormality, as determined by physical examination or abnormal baseline laboratory screening tests
  - ALT above normal range Table 8
  - Creatinine above normal range as defined in Table 8
  - Hemoglobin below normal range as defined in Table 9
  - Platelet count below normal range as defined in Table 9
  - Total white cell count below normal range as defined in Table 9
- Acute disease at the time of enrollment (acute disease is defined as the presence of a moderate or severe illness with or without fever). All vaccines can be administered to persons with a minor illness, such as diarrhea or mild upper respiratory infection without fever, i.e. Oral temperature < 37.5°C.
- Hepatomegaly, right upper quadrant abdominal pain or tenderness
- Administration of immunoglobulins and/or any blood products within the three months preceding the first dose of study vaccine or planned administration during the study period
- Pregnant or lactating female
- Suspected or known current alcohol abuse/drug abuse as obtained by history and physical examination
- Female who is willing or intends to become pregnant during the study
- Any history of allergic reaction or anaphylaxis to previous vaccination
- Unwilling to allow blood samples to be stored for future use
- Inability to make follow up visits
- Allergy to kanamycin, nickel, or imidazole
- Any other significant finding that in the opinion of the investigator would increase the risk of having an adverse outcome from participating in this study
- Previous allergy to Rabies Vaccine
- Allergy to chicken and chicken products

## Elimination criteria during the study

The following criteria should be checked at each visit subsequent to the first vaccination

- Use of any investigational or non-registered product (drug or vaccine) other than the study vaccine(s) during the study period.
- Administration of chronic (defined as more than 14 days) immunosuppressants or other immune modifying drugs within six months of vaccination. For corticosteroids, this is defined as prednisone, or equivalent, ≥ 0.5 mg/kg/day. Inhaled and topical steroids are allowed.
- Administration of immunoglobulins and/or any blood products during the study period
- Clinical evidence of drug abuse, intoxication during follow-up visits, or other medical condition that would increase the risk of an adverse event or possible harm to the volunteer from further participation in this study.
- Poor Reliability/Keeping Appointments
- Any other concern of the PI or other investigator that precludes volunteer involvement in the study

### Contraindications to vaccination

**Absolute contraindications to further vaccination**

The following adverse events (AEs) constitute absolute contraindications to further administration of FMP010/AS01B or rabies vaccine Rabipur®; if any of these AEs occur during the study, the subject must not receive additional doses of vaccine but may continue other study procedures at the discretion of the investigator. The subject must be followed until resolution of the event, as with any AE.

- Acute allergic reaction, significant IgE-mediated event or anaphylactic shock following the administration of vaccine investigational product;
- Pregnancy, i.e. a positive urine test.

**Indications for deferral of vaccination**

The following AEs constitute contraindications to administration of FMP010/AS01B or rabies vaccine, Rabipur, at that point in time. If any one of these AEs occurs at the time scheduled for administration of investigational product, the investigational product may be administered to the subject at a later date but within the time window specified in the protocol or withdrawn at the discretion of the investigator. The subject must be followed until resolution of the event, as with any AE.

- Acute disease at the time of administration of investigational product (acute disease is defined as the presence of a moderate or severe illness with or without fever). All vaccines can be administered to persons with a minor illness such as diarrhea or mild upper respiratory infection without fever, i.e. Oral temperature <37.5°C
- Oral temperature of ≥37.5°C

# CONDUCT OF STUDY

## Principal Investigators and Monitors

Dr. Otsyula and Dr Polhemus are the investigators for USAMRU-K Phase (Part B) of this study and are responsible for the conduct of the protocol, the actions of all other investigators, the safety of the volunteers, and all documentation and reporting. The PI is also responsible for training and maintaining all training files for all staff.

Associate investigators will be involved in screening of patients, recording of solicited and unsolicited symptoms, and initial evaluation of adverse events. All investigators who will review the subjects will have a copy of their medical license in the study file at the Regulatory Affairs Office.

Dr. Amos Otedo is the medical monitor from Kenya assigned to this study. He is a qualified physician not associated with this protocol who is able to monitor the volunteers during the conduct of the study and ensure that adequate medical care is provided for conditions that may arise during the study. He will review all SAEs (per ICH definitions) associated with the protocol and provide an unbiased written report of the event within 10 calendar days of the initial report. At a minimum, the medical monitor will comment on the outcomes of SAE and relationship of the SAE to the test article. The medical monitor will also indicate whether he/she concurs with the details of the report provided by the study investigator.

## 5.2 Ethics and regulatory considerations

The study will be conducted IAW ICH GCP, the Common Rule 32 CFR 219, applicable Kenyan guidelines; and OTSG and HSSRB regulations and policies

### Institutional Review Board/Independent Ethics Committee (IRB/IEC)

The IRBs must be constituted according to the local laws/customs of each participating country. For all trials conducted under the regulatory authority of the US FDA and under this IND application, it is required that the approving IRB/IEC be duly constituted and function IAW 21 CFR 56.

### Informed consent

- Informed consent will be obtained IAW 21 CFR 50.25 and the principles of the 1996 version of the Declaration of Helsinki.
- Information will be given in both oral and written form whenever possible and deemed appropriate by the IRB. The written consent document will embody the required and all applicable optional elements of informed consent as described in 21 CFR 50.25 and the 1996 version of the Declaration of Helsinki. In addition, the consent document will adhere to the ICH Harmonized Tripartite Guideline for GCP (E6) and will comply with DoD policy and regulations. An investigator or designate will describe the protocol to potential subjects. The Subject Information and Consent Form may be read to the subjects, but, in any event, the investigator or designate shall give the subjects ample opportunity to inquire about details of the study and ask any questions before dating and signing the Consent Form. Subject information and consent forms must be in a language fully comprehensible to the prospective subjects. All illiterate individuals will have the study, the Subject Information and Consent Form explained to them point by point by the interviewer in the presence of an impartial witness.
- Each subject’s signed informed consent form must be kept on file by the investigator for possible inspection by Regulatory Authorities and/or GSK Biologicals’ professional and Regulatory Compliance persons. The subjects should receive a copy of the signed and dated written informed consent form and any other written information provided to the subjects.

**Audits and Inspections**

Knowledge of any pending compliance inspections/visit by the FDA, OHRP, or other government agency concerning clinical investigation or research, the issuance of Inspection Reports, FDA Form 483, warning letters or actions taken by any Regulatory Agencies including legal or medical actions and any instances of serious or continuing noncompliance with the regulations or requirements will be reported immediately to the KEMRI ERC and WRAIR IRB. WRAIR IRB will report to USAMRMC ORP/HRPO as per SOP UWZ-C-636.

## 5.3 General Study Aspects

The community in which the study will take place will be informed about the nature and design of the study. Community leaders (assistant chiefs and local village elders) will be formally briefed in their own language on the nature and purpose of the study. They, in addition to the study volunteers, will have the opportunity to ask questions of the Investigator or his designees.

Adults aged 18 to 50 years will be recruited from the villages around Kisumu West District. Non-coercive means of recruitment will be used according to existing U.S. Army regulations (viz., AR 70-25 and AR 40-38). It is estimated based on previous clinical trial experience at Kisumu West District that approximately five times the number of volunteers to be enrolled will need to be screened in order to reach the target number of 30; therefore approximately 150 volunteers will be screened. Prospective subjects will view a videotaped briefing on the nature and purpose of the study and will then receive both oral and written explanations of the study. Afterwards, written informed consent will be obtained from each person who wants to participate in the study. No question relating to the potential study subjects health will be asked prior to consenting. To verify age, study staff will check national ID card or birth certificates, or letter from chief/elder of the village. So that language and illiteracy will not be impediments to informed consent, all briefings and explanations will be in the local language.

### Screening

Volunteers with a written Informed Consent, signed/thumb printed and dated will be eligible to be screened for Part B of the trial. Screening evaluation will include:

- a medical history through a one-on-one interview with a clinician.
- physical examination
- laboratory tests, which include complete blood count (CBC) using ACT 5 Diff or its equivalent.
- Serum glucose, creatinine, AST, ALT, alkaline phosphatase, total Bilirubin using the Selectra E Autoanlyzer or its equivalent.
- HIV as below described
- genotype for Sickle Cell and G6PD status using Biorad Analyzer or its equivalent.
- Alpha Thalassaemia
- Urine β-HCG will be obtained on all female volunteers and serum β-HCG for confirmation where necessary.
- Volunteers may be screened more than once if re-screening is determined to be necessary (e.g. after treatment for malaria).

Volunteers will be excluded from participation if they have any of the exclusion criteria indicated in section 4.4 above or have any other finding that would increase the risk of having an adverse outcome during the conduct of the trial. The reason for exclusion will be discussed with the volunteer.

For HIV, rapid tests will be used for screening. (Determine and Unigold) will be used to carry out the test. Western blot will be done as the confirmatory test for those with indeterminate results. . Volunteers found to be seropositive for HIV will be counseled by a member of the study staff and referred to a HIV care center for further evaluation.

All screening tests will be completed within 60 days prior to entry into the study. Information gathered during screening (medical history, physical examination, and laboratory analysis) will be recorded in the Source Documents and, for enrolled volunteers, also on the case report forms (CRFs).

A photograph will be taken of each volunteer who is screened and stored securely in a computer at the CTC. When screening and review of inclusion/exclusion criteria are complete, a picture ID card of each eligible volunteer will be created using the stored photo. The ID card will include the subject’s name, photo, subject number, study number, study name, and contact information for the CTC. This will ensure that the subject can contact an investigator and/or the CTC. Copies of these pictures will be kept in the subjects records to aid the study staff in confirmation of the volunteers’ identification for future visits. The photos of subjects who are determined ineligible to participate will be deleted from the computer.

At the screening visit each subject will be given a Screening Number and a screening record will be initiated. Volunteer Study Numbers will be issued at randomization for the first vaccination. They will be issued consecutively in the order in which the subjects arrive at the clinical trials center. This Study Number will be used throughout the study to identify every document and blood sample associated with the volunteer.

Screening records will be converted to Study Records if the volunteer is randomized at the first vaccination visit. The Study Record will contain the Volunteer’s Study Number, date of birth, demographics, screening results, and all study information.

Clinical lab tests will be performed by the WRP lab or coordinated with a local reference lab.

### Study Design

This trial will be conducted in two parts. Part A will be conducted in the US at WRAIR and Part B in at the Walter Reed Project/KEMRI in Kisumu, Kenya. A total of 56 volunteers will be enrolled with 26 enrolling in part A and 30 in part B. For Part B, 20 volunteers (minimum 16) will be enrolled to receive FMP010 at 50 µg in AS01B (Group 3) and 10 (minimum 8) volunteers enrolled to receive a comparator rabies vaccine Rabipur (Group 4). This part will be randomized and blinded.

## 5.4 Safety considerations for the trial

A Safety Monitoring Committee (SMC) will be appointed to review the safety data from Part A (USA). The SMC will consist of a three experienced clinicians not already participating on the study team recommended by the PI and approved by the study partners. The primary responsibilities of the SMC are to review and evaluate the accumulated study data for participant safety after first vaccination of Group 1, after second vaccination of Group 2, and as needed if safety concerns should arise in other times during the trial, and make recommendations to the PI concerning the continuation, modification or termination of the study. It is not the role of the SMC to comment on the study design, conduct or interpretation, but rather to act as independent experts in evaluating adverse events. The SMC may, if deemed necessary, convene a meeting with, or request further information from the PI or the Medical Monitor.

### Procedure for Evaluating Vaccine Safety in Part A and Progression to Part B

Vaccination and review of the safety data for Groups 1 and 2 (Part A in the USA) will occur before vaccination can begin in Part B (Kisumu, Kenya). Safety data for Groups 1 and 2 (Part A in the USA) will be collected and submitted to a Safety Monitoring Committee (SMC) for recommendations about progression to Part B in Kisumu, Kenya. If progression to Part B is recommended the safety data will be presented to the Medical Monitor for Part B and the KEMRI Ethical Review Committee for evaluation and recommendation to initiate Part B of the study.

### Procedure for Evaluating Vaccine Safety in Part B and Progression to the Next Dose

The following Hold Rule will be used to determine if Part B of the study can progress to the second and third vaccination dose

### Holding Rule for Progression to Next Dose in Part B

- Vaccination with the next dose can be put on hold if five subjects develop a Grade 3 adverse event beginning within two days after vaccination, and persisting at Grade 3 for ≥ 72 hours if the adverse event is malaise, myalgia, fatigue, nausea, fever, headache, or joint pain.

Activation of the Holding Rule requires a thorough review by the Local Medical Monitor in Kenya (Part B, Kisumu, Kenya) and discussion between the investigators, the Medical Monitors, the safety monitoring committee, and Sponsor. Vaccination will restart only if all parties agree to a resumption of vaccination.

### Stopping Rules

The following Stopping Rules for individual subjects will apply (i.e., the withdrawal of an individual subject from further vaccination):

Stopping Rules:

A. Local Reactions: Investigator discretion

B. Systemic adverse events: Individual subjects will be withdrawn from further vaccination if they develop a Grade 3 adverse event beginning within two days after vaccination, and persisting at Grade 3 for ≥ 72 hours if the adverse event is malaise, myalgia, fatigue, fever, headache, or joint pain.

### Risks to the Subjects

The most frequent adverse reactions observed in previous clinical trials using the AS01B adjuvant system with other recombinant protein antigens includes pain, swelling, erythema, and tenderness at the site of injection, and systemic symptoms such as low-grade fever and short-term flu-like symptoms such as fatigue, myalgia, headache, malaise. There is always the remote possibility of a serious and potentially fatal hypersensitivity reaction to the vaccine or one of its components. There have been no reports of allergic reactions in previous malaria vaccine studies at WRAIR using the AS02A adjuvant, including two recent studies in 2006 (MAL-052, MAL-045) where a total of 33 volunteers received a malaria vaccine candidate formulated in the adjuvant AS02A (personal communication J. Cummings). Although FMP010/AS01B has not been used in humans, over 360 adults have received AS01B with differing antigen formulations (personal communication O. Ofori-Anyinam), including several studies conducted at WRAIR. In MAL-027, a study of RTS,S in AS01B involving 52 vaccinated adult volunteers, one mild, transient urticarial reaction was reported. In MAL-052 and MAL-045, no urticarial reactions were seen in 38 volunteers receiving AS01B with antigens LSA-1 (Liver Stage Antigen-1) or AMA-1 (Apical Membrane Antigen-1). One volunteer in MAL-045 was discontinued from the study after the second vaccination due to a persistent non-uticarial, papular rash at both injection sites occurring 3 weeks after the second vaccination.

Because this is an experimental vaccine there may be unknown risks. By participating in this study, the subjects will undergo phlebotomy, which carry its own associated risks of vascular damage, bruising, and clot formation. Although every measure will be taken to assure the confidentiality of subjects in this study by assigning alphanumeric codes as a study participant identifier, there is the theoretical risk associated with breach of confidentiality by participating in this study.

#### Precautions to Minimize Risk

As outlined above, the volunteers will be monitored closely during their participation in this study. The study vaccine has been prepared according to Good Manufacturing Practices (GMP). The vaccines will be administered under the supervision of an investigator and nurses equipped with drugs and equipment to immediately treat anaphylaxis.

The vaccine will be tested at 1/5^th^ and full dose in healthy adults in the US, prior to any vaccinations in Kenya. Additionally, the data from the US will be reviewed by a data safety committee prior to any vaccinations in Kenya. Only the full dose (50 µg) of FMP010 will be administered in Kenya under part B of the protocol.

Phlebotomy will be done by aseptic technique by trained staff, to reduce the risk of complications.

### Risks to Study personnel

The principal risk in the clinical setting is attendant with the risk of handling of needles that may be contaminated with blood and body fluids and include risks of contracting hepatitis B and C, human immunodeficiency virus (HIV), and other human pathogens. Adherence to Standard Operating Procedures (SOP) for working with infectious agents, and using universal precautions will reduce the risk of exposure for individuals working in this setting. There are no known risks from the working environment other than those associated with the generation of biohazardous wastes attendant to vaccination of humans. All biohazardous wastes will be disposed of as stipulated by local regulations.

**Method of blinding**

The sponsor will generate a randomization list which will be made available to the study site and both the local medical and the WRAIR medical monitors prior to the onset of the study.

Data pertaining to FMP010/AS01B or control vaccines will be collected in a double blinded (observer blind) manner. ‘Double blinded (observer blind)’ means that the vaccine recipient as well as those responsible for the evaluation of study endpoint data will all be unaware which treatment, FMP010/AS01B or control vaccine, was administered to a particular subject. The vaccines in this study are of different volumes. The contents of the syringe will be masked with an opaque label to ensure blinding. The only study staff aware of the vaccine assignment for FMP010/AS01B or control vaccine will be those responsible for preparation of the vaccines; these staff will play no other role in the study which would require clinical evaluation.

**Breaking the study blind**

The code break envelopes will contain information associating a patient alphanumeric identification number to specific vaccine. If deemed necessary for reasons such as safety, the local medical monitor will unblind a specific participant without revealing the study blind to the investigator. The code will be broken in the case of medical events that the investigator/physician in charge of the participant feels cannot be treated without knowing the identity of the study vaccine. At the conclusion of the study, the PI and sub-investigators will agree upon a date and procedure for breaking the study blind and begin data analysis.

After unblinding, subjects who received FMP010/ASO1B will be given the opportunity to receive the rabies vaccine.

**Replacement of unusable vaccine doses**

If a vaccine dose needs replacement, the envelope with the corresponding treatment number will designate the replacement without unblinding the study

## 5.5 Outline of study procedures

### Screening and Vaccination phase

All subjects enrolled in the study will be issued an emergency notification card that details their participation in the study and specific contact phone numbers of the investigators. Vitals signs (temperature, pulse, blood pressure) are recorded at all scheduled study visits unless indicated otherwise.

The schedule below applies to Part B of the protocol and the Day numbers refer to volunteer day of study not study date.

**Visit 1: Day-60 to -1 – Screening/enrollment of volunteers**

- Briefing of the potential volunteer
- Written Informed Consent (Appendix E )
- Informed consent for HIV testing (Appendix F)
- Provision of medical history by volunteer
- Physical examination
- Urine β-HCG pregnancy test for all female adult volunteers
- Check of inclusion and exclusion criteria
- Blood collection:
  - **Part B (Kenya):** Approximately 7 mL whole venous blood will be collected for the determination of:
    - Complete blood count (CBC)
    - Serum glucose, creatinine, AST, ALT, alkaline phosphatase, total bilirubin
    - HIV
    - Sickle Cell genotype
    - G6PD genotype
    - Alpha Thalassaemia

**Visit 2: Day 0 – Vaccination 1**

- Check/verify inclusion/exclusion criteria and contraindications/precautions
- History and history-directed physical examination and recording of pre-vaccination data including vital signs
- Collection of urine for β-HCG pregnancy tests where applicable
- Blood collection:
  - **Part B (Kenya):** Approximately 11 mL whole venous blood will be collected:
    - 5 mL for a CBC, creatinine, AST and ALT
    - 5 mL for humoral immune studies (ELISA, GIA). The research samples will be centrifuged and the serum stored at ‑20°C (± 5°) or colder until processing.
    - 1 mL for *P. falciparum* antigens detection by HRP 2 and pLDH ELISAs

- Vaccination: IM administration of FMP010/AS01B candidate malaria vaccine(or Rabipur® vaccine for Part B) in the deltoid muscle of the non-dominant arm

NOTE: Each vaccinee will be closely observed for at least 30 minutes following vaccination. After observation, blood pressure (BP), pulse, oral temperature, and solicited and unsolicited symptoms will be reviewed. Volunteers will be instructed to contact the study team immediately should they manifest any signs or symptoms they perceive as serious.

**Visits 3-6: Days 1, 2, 3, 7 (±3 day) – Post-vaccination 1 follow-up (f/u) visits**

- Recording of solicited and unsolicited symptoms and medication by the health care provider. If Grade 3 AE noted, the PI or other physician investigator must be notified.
- History directed physical exam
- Check of elimination criteria
- Blood collection (Day 7):

**Part B (Kenya):** Approximately 6 mL whole venous blood will be collected for:

- - - Complete blood count (CBC)
    - Serum creatinine, AST,ALT
    - *P. falciparum* antigens detection by HRP 2 and pLDH ELISAs

**Visits 7: Day 14 (±3 day) – Post-vaccination 1, 14-day follow-up (f/u) visit**

- History directed physical examination
- Record any unsolicited adverse events occurring after the last vaccine dose.
- Check elimination criteria; recording of medications
- Blood collection:
  - Part B **(Kenya)**:.Approximately 6 mL of venous blood will be collected for:
    - 5 mL for humoral immune studies. The research samples will be centrifuged and the serum stored at ‑20°C (± 5°) or colder until processing
    - 1 mL for *P. falciparum* antigens detection by HRP 2 and pLDH ELISAs

**Visit 8: Day 28 (±3 days) – Vaccination 2**

- Check elimination criteria, contraindications/precautions and record medications
- Record any unsolicited adverse events occurring after the last vaccine dose
- History, directed physical examination
- Collection of urine for β-HCG pregnancy tests where applicable
- Blood collection
  - **Part B (Kenya):** Approximately 11 mL whole venous blood will be collected:
  - 5 mL for humoral immune studies. The research samples will be centrifuged and the serum stored at ‑20°C (± 5°) or colder until processing.
  - 5 mL for CBC, creatinine, ALT, AST
  - 1 mL for *P. falciparum* antigens detection by HRP 2 and pLDH ELISAs
- Vaccination: IM administration of FMP010/AS01B candidate malaria vaccine (or Rabipur® vaccine for Part B) in the deltoid muscle of the non-dominant arm.

NOTE: Each vaccinee will be closely observed for at least 30 minutes following vaccination after which blood pressure (BP), pulse, oral temperature, and solicited and unsolicited symptoms will be reviewed by the PI or his designee. Volunteers will be instructed to contact the investigator immediately should they manifest any signs or symptoms they perceive as serious.

**Visits 9-12: Days 29, 30, 31, 35 (±3 days) – Post-vaccination 2 f/u visits**

- Daily recording of solicited and unsolicited symptoms and medication by the PI or his designee
- Check of elimination criteria; recording of medications
- Blood collection (Day 35)
  - **Part B (Kenya):** Approximately 6 mL whole venous blood will be collected for:
    - Complete blood count (CBC)
    - Creatinine, AST,ALT
    - *P. falciparum* antigens detection by HRP 2 and pLDH ELISAs

**Visit 13: Day 42 (±5 days) – Post-vaccination 2, 14-day f/u visit**

- History directed physical examination
- Record any unsolicited adverse events occurring after the last vaccine dose.
- Check elimination criteria; recording of medications
- Blood collection:
  - Part B **(Kenya)**: Approximately 6 mL of venous blood will be collected:
  - 5 mL for humoral immune studies. The research samples will be centrifuged and the serum stored at ‑20°C (± 5°) or colder until processing.
  - 1 mL for *P. falciparum* antigens detection by HRP 2 and pLDH ELISAs

**Visit 14: Day 56 (±3 days) – Vaccination 3**

- Check elimination criteria, contraindications/precautions, record medications
- History, directed physical examination
- Record any unsolicited adverse events occurring after the last vaccine dose
- Collection of urine for β-HCG pregnancy tests where applicable
- Blood collection:
  - **Part B ( Kenya):** Approximately 11 mL whole venous blood will be collected for:
  - 5 mL for humoral immune studies. The research samples will be centrifuged and the serum stored at ‑20°C (± 5°) or colder until processing.
  - 5 mL for a CBC, creatinine ALT, AST.
  - 1 mL for *P. falciparum* antigens detection by HRP 2 and pLDH ELISAs
- Vaccination: IM administration of FMP010/AS01B candidate malaria vaccine (or Rabipur® vaccine for Part B) in the deltoid muscle of the non-dominant arm.

NOTE: Each vaccinee will be closely observed for at least 30 minutes following vaccination after which blood pressure (BP), pulse, oral temperature, and solicited and unsolicited symptoms will be reviewed by the PI or his designee. Volunteers will be instructed to contact the investigator immediately should they manifest any signs or symptoms they perceive as serious.

**Visits 15-18: Days 57, 58, 59, 63 (±3 days) – Post-vaccination 3 f/u visits**

- Daily recording of solicited and unsolicited symptoms and medication by the PI or his designee
- Check of elimination criteria; recording of medications
- Blood collection (Day 63):
  - **Part B (Kenya):** Approximately 6 mL whole venous blood will be collected for:
    - Complete blood count (CBC)
    - Serum creatinine, AST,ALT
    - 1 mL for *P. falciparum* antigens detection by HRP 2 and pLDH ELISAs

**Visit 19: Day 70 (± 5 days) – Post-vaccination 3, 14-day f/u visit**

- Check elimination criteria; record medications
- History directed physical examination
- Record any unsolicited adverse events occurring after the last vaccine dose.
- Blood collection:
  - Part B **(Kenya)**: Approximately 6 mL whole venous blood will be collected for:
  - 5 mL for humoral immune studies. The research samples will be centrifuged and the serum stored at ‑20°C (± 5°) or colder until processing.
  - 1 mL for *P. falciparum* antigens detection by HRP 2 and pLDH ELISAs

**Visit 20: Day 86 (±3 days) – Post-vaccination 3, 30-day f/u visit**

- Check elimination criteria, contraindications/precautions, record medications
- History, directed physical examination
- Record any unsolicited adverse events occurring after the last vaccine dose

**Visit 21: Day 112 (± 7 days) – Final Study visit**

- Check elimination criteria; record medications
- History and directed physical examination
- Record any unsolicited adverse events occurring after the last vaccine dose
- Collection of urine for pregnancy tests where applicable
- Blood collection - whole venous blood will be collected:

**Part B (Kenya):** Approximately 11 mL whole venous blood will be collected for:

- - 5 mL for a CBC, creatinine ALT, and AST.
  - 5 mL for humoral immune studies. The research samples will be centrifuged and the serum stored at ‑20°C (± 5°) or colder until processing.
  - 1 mL for *P. falciparum* antigens detection by HRP 2 and pLDH ELISAs

Table 2: Time and event Schedule for Groups 3 & 4

| **Part B (Kenya): Vaccination Phase** | **Screen** |  | | |  | | |  | | |  | | | | | | | |
| --- | --- | --- | --- | --- | --- | --- | --- | --- | --- | --- | --- | --- | --- | --- | --- | --- | --- | --- |
| Day | -60 to -1 | 0 | 1-6 | 7 | | 14 | 28 | | 29-34 | 35 | | 42 | 56 | 57-62 | 63 | 70 | 86 | 112 |
| Visit | 1 | 2 | 3-5 | 6 | | 7 | 8 | | 9-11 | 12 | | 13 | 14 | 15-17 | 18 | 19 | 20 | 21 |
| **Vaccination with FMP010 / AS01B or *Rabipur*** |  | **1** |  |  | |  | **2** | |  |  | |  | **3** |  |  |  |  |  |
| Screening | • |  |  |  | |  |  | |  |  | |  |  |  |  |  |  |  |
| Briefing | • |  |  |  | |  |  | |  |  | |  |  |  |  |  |  |  |
| Informed consent – for study and HIV (part B – Kenya) testing | • |  |  |  | |  |  | |  |  | |  |  |  |  |  |  |  |
| Comprehension Assessment | • |  |  |  | |  |  | |  |  | |  |  |  |  |  |  |  |
| Check of inclusion criteria | • | • |  |  | |  |  | |  |  | |  |  |  |  |  |  |  |
| Check of exclusion criteria | • | • |  |  | |  |  | |  |  | |  |  |  |  |  |  |  |
| Medical history | • |  |  |  | |  |  | |  |  | |  |  |  |  |  |  |  |
| Physical examination | • |  |  |  | |  |  | |  |  | |  |  |  |  |  |  |  |
| **Vaccination** |  | • |  |  | |  | • | |  |  | |  | • |  |  |  |  |  |
| Pre-vaccination assessment |  | • |  |  | |  | • | |  |  | |  | • |  |  |  |  |  |
| Post-vaccination assessment |  | • |  |  | |  | • | |  |  | |  | • |  |  |  |  |  |
| **Follow-up visit** |  |  | • | • | | • |  | | • | • | | • |  | • | • | • | • | • |
| Check elimination criteria and record medications |  |  | • | • | | • |  | | • | • | | • |  | • | • | • | • | • |
| Recording of unsolicited adverse events |  |  | • | • | | • |  | | • | • | | • |  | • | • | • | • |  |
| Recording of solicited adverse events  History and directed physical examination |  |  |  |  | | • |  | |  |  | | • |  |  |  | • | • | • |

Table 3: Time and event Schedule for Groups 3 & 4

| **Part B (Kenya): Vaccination Phase** | **Screen** |  | |  | | |  | |  |  | | | | | | | |
| --- | --- | --- | --- | --- | --- | --- | --- | --- | --- | --- | --- | --- | --- | --- | --- | --- | --- |
| Day | -75 to -1 | 0 | 1-6 | | 7 | 14 | | 28 | 29-34 | 35 | 42 | 56 | 57-62 | 63 | 70 | 86 | 112 |
| Visit | 1 | 2 | 3-5 | | 6 | 7 | | 8 | 9-11 | 12 | 13 | 14 | 15-17 | 18 | 19 | 20 | 21 |
| **Vaccination with FMP010 / AS01B or *Rabipur*** |  | **1** |  | |  |  | | **2** |  |  |  | **3** |  |  |  |  |  |
| **Laboratory analyses** |  |  |  | |  |  | |  |  |  |  |  |  |  |  |  |  |
| Screening (7 mL blood): | •  • |  |  | |  |  | |  |  |  |  |  |  |  |  |  |  |
| - HIV-1 |  |  |  | |  |  | |  |  |  |  |  |  |  |  |  |  |
| - glucose, alkaline phosphatase, total bilirubin, creatinine, AST, ALT |  |  |  | |  |  | |  |  |  |  |  |  |  |  |  |  |
| - CBC  - G6PD and Sickle Cell Genotype  - Alpha Thalasaemia |  |  |  | |  |  | |  |  |  |  |  |  |  |  |  |  |
| Pregnancy test: Urine β-HCG | • | • |  | |  |  | | • |  |  |  | • |  |  |  |  |  |
| **Biochemical analysis:** |  | • |  | | • |  | | • |  | • |  | • |  | • |  |  | • |
| (5 mL blood): safety labs CBC, creatinine, AST, ALT |  |  |  | |  |  | |  |  |  |  |  |  |  |  |  |  |
| **Antibody response:** |  | • |  | |  | • | | • |  |  | • | • |  |  | • |  | • |
| (5 mL blood): ELISA, GIA |  |  |  | |  |  | |  |  |  |  |  |  |  |  |  |  |
| **Antigen Detection:** |  |  |  | |  |  | |  |  |  |  |  |  |  |  |  |  |
| (1 mL blood): HRP-2 and pLDH |  | • |  | | • | • | | • |  | • | • | • |  | • | • |  | • |
| **Blood volume in mL per visit:** | 7 | 11 | - | | 6 | 6 | | 11 |  | 6 | 6 | 11 |  | 6 | 6 |  | 11 |
| Cumulative blood volume for study participation: | 7 | 18 |  | | 24 | 30 | | 41 |  | 47 | 53 | 64 |  | 70 | 76 |  | 87 |

• indicates a study procedure that requires documentation in the CRF

### Protocol Deviations

A protocol deviation is defined as an isolated occurrence involving a procedure that did not follow the study protocol, or study specific procedures. All staff involved in the conduct of a clinical trial shall be aware of the specific protocol requirements for completing study visits and notify the PI in the event of any breach of protocol.

Procedure:

1. Notify the PI as soon as possible if not present when the deviation is discovered.
2. Document in the volunteer’s study chart all protocol deviations directly involving or affecting the volunteer. A thorough explanation of the circumstances leading up to the deviation is to be included.
3. Report a protocol deviation(s) according to the *Guidelines for Investigators: Requirements for U.S. Army Medical Research and Materiel Command (USAMRMC) Headquarters Review and Approval of Research Involving Human Volunteers, Human Anatomical Substances and/or Human Data.*
4. The timeline for reporting protocol deviations to the Division of Human Subjects Protection (DHSP)/WRAIR Institutional Review Committee (WRAIR IRB), KEMRI ERC and Human Research Protections Office (HRPO) at USAMRMC, is determined by the categorization of the deviation: (a) significant or (b) minor.
   1. **Significant deviations** are departures from protocol that have a significant impact on the welfare or safety of a volunteer or on the integrity of the study data. Examples: administering the wrong test article to a volunteer; failure to obtain a scheduled blood draw for multiple participants. Major or Significant deviations that occur in greater than minimal risk protocol will be reported to KEMRI IRB within the first 24 hours and also promptly reported (within 48 hours) to the WRAIR IRB by phone (301 319-9940), by email (WRAIRDHSP@amedd.army.mil), or by facsimile (301-319-9961) to the Division of Human Subjects Protection, Walter Reed Army Institute of Research, 503 Robert Grant Ave., RM 1W30, Silver Spring, Maryland, 20910-7500, All reports will be submitted with a cover memo naming the protocol, KEMRI SSC number, WRAIR and HSRRB log numbers, the principal investigator, the time period covered, and any exceptional events that occurred.
   2. **Minor deviations** are routine departures that typically involve a volunteer’s failure to comply with the protocol. Examples: missing scheduled visits; failing to return diary cards. Minor deviations that occur in greater than minimal risk protocols will be reported to KEMRI IRB and DHSP/WRAIR IRB and thus HRPO in the Continuing Review Report(s).

## Sample handling and analysis

### Serology plan

Separation of serum from the venous blood will be performed at the WRP Kisumu laboratory, and serum frozen at -20° C. No personal identifying information will be indicated on any sample or vial other than the volunteer code, study number and date within the study. Serum samples will be collected in multiple NUNC tubes and will be distributed to the laboratories performing the GIA and ELISA assays. .

The time points for humoral assays are located in Tables 2 and 3; however, performing testing at time points less frequent than indicated is at the discretion of the researcher conducting such tests.

Growth inhibition assays and MSP1 ELISAs will be performed at the WRAIR and/or at the WRP-KEMRI laboratory in Kisumu, Kenya.

Laboratory assays

Screening and follow-up diagnostic laboratory testing will be performed at the WRP-KEMRI clinical laboratories in Kombewa/Kondele or local reference laboratories.

Additional laboratory assays may be performed on stored serum samples left over after protocol specified laboratory testing is completed. This may include clinical tests performed during the evaluation of a volunteer’s medical problem or additional malaria specific immunologic evaluations as they become available.

# STUDY VACCINE AND ADMINISTRATION

##

## Study vaccine

### Name and Description of the Investigational Product

***FMP010 antigen:*** The antigen FMP010 is a recombinant subunit protein produced in and purified from *E. coli* bacteria. FMP010 is a lyophilized preparation of the 42-kDa carboxy-terminal end of MSP1 comprising 355 amino acids derived from the merozoite surface protein MSP1 of the malaria parasite*, P. falciparum* plus 16 non-MSP1 amino acids fused at the N-terminus and produced in and purified from *E. coli* bacteria. The protein is expressed as a 6-His fusion protein. The antigen will be adjuvanted in 0.5 mL of AS01B.

### Name and Description of the Control Vaccine

Rabipur® vaccine is manufactured in India by Chiron Behring Vaccines Pvt, Ltd) and supplied in single dose vials containing lyophilised antigen with 1.0 mL of diluent (sterile water) for injection.

**Rationale for dosing plan:**

Rabipur® will be dosed at 0, 1, and 2 months rather than the standard regimen (Day 0, Day 7 and Day 21/28). This is an off-lable application and has been shown to lead to an acceptable antibody level in a study done by [Nicholson KG](http://www.ncbi.nlm.nih.gov/sites/entrez?Db=pubmed&Cmd=Search&Term="Nicholson KG"%5BAuthor%5D&itool=EntrezSystem2.PEntrez.Pubmed.Pubmed_ResultsPanel.Pubmed_DiscoveryPanel.Pubmed_RVAbstractPlus), [Farrow PR](http://www.ncbi.nlm.nih.gov/sites/entrez?Db=pubmed&Cmd=Search&Term="Farrow PR"%5BAuthor%5D&itool=EntrezSystem2.PEntrez.Pubmed.Pubmed_ResultsPanel.Pubmed_DiscoveryPanel.Pubmed_RVAbstractPlus), [Bijok U](http://www.ncbi.nlm.nih.gov/sites/entrez?Db=pubmed&Cmd=Search&Term="Bijok U"%5BAuthor%5D&itool=EntrezSystem2.PEntrez.Pubmed.Pubmed_ResultsPanel.Pubmed_DiscoveryPanel.Pubmed_RVAbstractPlus), [Barth R](http://www.ncbi.nlm.nih.gov/sites/entrez?Db=pubmed&Cmd=Search&Term="Barth R"%5BAuthor%5D&itool=EntrezSystem2.PEntrez.Pubmed.Pubmed_ResultsPanel.Pubmed_DiscoveryPanel.Pubmed_RVAbstractPlus) .

In the same study, it was shown that the antibody level at two years was comparable to that induced by the standard regimen.

### Adjuvants

AS01B contains 50 μg of MPL®, 50 μg QS21 (QS21 is a triterpene glycoside purified from the bark of *Quillaja saponaria*) and liposomes.

## Dosage and administration

Reconstitution of vaccines with AS01B and administration:

Group 3: 1 vial of Final Container FMP010, Lot 1157

Disinfect top of a vaccine vial with the antigen cake with alcohol swabs - let dry.
Aspirate the contents of the AS01B vial with a syringe and inject adjuvant into the vial of lyophilized antigen and remove needle from vial.
Dissolve the cake by gently shaking the vial. Wait for approximately one minute to ensure complete dissolution of vial contents before withdrawing 0.5 mL of the reconstituted vaccine solution using a fresh needle and syringe.

Group 4 (Rabipur® rabies vaccine): Rabipur® rabies vaccine is a licensed vaccine and will be prepared in accordance with the package insert.

Each vaccine dose should be administered by slow intramuscular injection in the deltoid muscle of the non-dominant arm within four hours of reconstitution. Prior to each vaccination, the non-dominant arm will be inspected for clinically evident physical findings that might interfere with post-vaccination assessment of reactogenicity. If such a finding is discovered, the dominant arm may be used at the discretion of the investigator.

The vaccines will be administered in the Muriithi Wellde Clinical Trials Centre (Part B) under the supervision of physicians skilled in the management of anaphylactic reactions.

Vaccine and adjuvant vials will be labeled with subject identifier and saved until study completion and discarded upon instructions from GSK Biologicals.

### Treatment allocation

Volunteers will be randomized to the candidate vaccine or the control arm when they present for vaccination 1.

## Storage

- All vials containing FMP010 will be stored at 2-8^o^C.
- All AS01B vials must be stored at 2-8^o^C.
- Rabies will be stored at 2-8^o^C.
- Storage temperatures will be recorded daily.
- Records must be maintained that document receipt, release for vaccination, disposal, or return, to the manufacturer of all vaccine vials.

### Packaging: See Appendix D

### Accountability: See Appendix D

### Replacement of unusable vaccine doses

Additional doses of vaccine and adjuvant will be provided to replace those that are unusable (see Appendix E for details of supplies). In case a vial or pre-filled syringe is broken or unusable, the person in charge of the vaccination will use a replacement dose. Although the sponsor need not be notified immediately in these cases, documentation of the use of the replacement dose and reason for using it must be recorded by the investigator on the vaccine administration page of the CRF and on the vaccine accountability form. Proper disposal of syringes will occur after each dose administration. Spent vaccine vials/ pre-filled syringes, labeled with the volunteers’ identification number, will be retained separately for confirmation of correct dosing if need be

# Concomitant medication/treatment

At each study visit/contact, the investigator will question the subject or their legal representative about any medication taken. Any immunosuppressants or other immune-modifying drugs or treatments, any vaccine other than the study vaccine(s) and any antipyretics administered at ANY time during the period starting 30 days prior to the first dose of study vaccine(s) and until study completion must be recorded in the CRF with trade name and/or generic name of the medication, medical indication, total daily dose, route of administration, start and end dates of treatment. Any other concomitant medication administered prophylactically in anticipation of reaction to the vaccination must also be recorded in the CRF with trade name and/or generic name of the medication, total daily dose, route of administration, start and end dates of treatment and coded as ‘Prophylactic’.

# ADVERSE EVENTS

The investigator is responsible for the detection and documentation of events meeting the criteria and definition of an adverse event (AE) or serious adverse event (SAE) as provided in this protocol. During the study, when there is a safety evaluation, the investigator or site staff will be responsible for detecting AEs and SAEs, as detailed in this section of the protocol. Each subject will be instructed to contact the investigator immediately should they manifest any signs or symptoms they perceive as serious.

## Eliciting and documenting adverse events

### Adverse event definition

An AE is any untoward medical occurrence in a study volunteer, temporally associated with the use of a test article, whether or not considered related to the test article. An AE can therefore be any unfavorable and unintended sign (including an abnormal laboratory finding), symptom, or disease (new or exacerbated) temporally associated with the use of a test article whether or not it is considered to be study related.

Examples of an AE include:

- Exacerbation of a chronic or intermittent pre-existing condition including either an increase in frequency and/or intensity of the condition.
- New conditions detected or diagnosed after investigational product administration even though it may have been present prior to the start of the study.
- Signs, symptoms, or the clinical sequelae of a suspected overdose of either investigational product or a concurrent medication (overdose per se should not be reported as an AE/SAE).
- Signs, symptoms temporally associated with vaccine administration.

AEs may include pre- or post-treatment events that occur as a result of protocol-mandated procedures (i.e., invasive procedures, modification of subject’s previous therapeutic regimen). Pre-existing conditions or signs and/or symptoms present in a subject prior to the start of the study (i.e. prior to the first study procedure) as well as any that are not recognized at study entry but are recognized during the study period, should be recorded in the medical history section of the subject’s CRF. Adverse events that occur after informed consent is obtained, but prior to vaccination, will be documented the Medical History form within the participant's CRF. Anticipated day-to-day fluctuations of pre-existing conditions, including disease under study, that do not represent a clinically significant exacerbation need not be considered adverse events. Adverse events should be documented in terms of medical diagnosis(ses) when possible. When this is not possible, the adverse event should be documented in terms of signs and symptoms observed by the investigator or reported by the participant at each study visit.

Although not considered as an adverse event, hospitalization for either elective surgery related to a pre-existing condition that did not increase in severity or frequency following initiation of the study, or for routine clinical procedures (including hospitalization for “social” reasons) that are not the result of an adverse event, must be recorded in the CRF. If the hospitalization arises from a pre-existing condition, or was planned prior to the first vaccination, it should be recorded in the Medical History form of the CRF. If the hospitalization arises from a pre-existing condition, and the hospitalization was planned after the first vaccination, it should be recorded in the adverse event page of the CRF. In both cases, it should be recorded as ‘Hospitalization,’ and the relationship to vaccination will be checked “No.”

*Surveillance period for occurrence of adverse events*

All adverse events occurring within one month (minimum 30 days) following administration of each vaccine must be recorded on the Adverse Event form in the participant's CRF, irrespective of severity or whether or not they are considered vaccination-related. See Section 8.5 for instructions for recording and reporting of serious adverse events.

*Recording adverse events*

At each visit/assessment, all adverse events either observed by the investigator or one of his clinical collaborators or reported by the participant spontaneously or in response to a direct question will be evaluated by the investigator. Adverse events not previously documented in the study will be recorded in the Adverse Event form within the participant's CRF. The nature of each event, date and time (where appropriate) of onset, outcome, intensity, and relationship to vaccination should be established. Details of any corrective treatment should be recorded on the appropriate page of the CRF.

Adverse events already documented in the CRF, i.e. at a previous assessment, and designated as ‘ongoing’ should be reviewed at subsequent visits, as necessary. If these have resolved, the documentation in the CRF should be completed.

N.B. If an adverse event changes in frequency or intensity during a study period, the highest intensity will be recorded. If the adverse event recurs with a defined interval of symptom-free time (>24 hours) in between episodes, new record of the event will be started.

Events that are not alarming and may reasonably be regarded as caused by or probably caused by the vaccine should be batched together and reported to the DHSP/WRAIR IRB and Sponsor’s Representative at the time of continuing review. See Section 8 for instructions for reporting and recording of unanticipated problems involving risk to others and/or serious adverse events.

### Solicited adverse events

Solicited symptoms are adverse events occurring within the seven days follow-up period after vaccination that are inquired about by the investigator or specified designee. Space on the source documents and CRF will be allocated for the recording of solicited symptoms. Table 4 lists the solicited local and general adverse events for the study.

Table 4: Solicited local and general adverse events

|  | **Adverse events** |
| --- | --- |
| **Local (injection site)** | Pain at the injection site |
|  | Swelling at the injection site |
|  | Erythema at the injection site |
| **General** | Fever* (Oral Temperature) |
|  | Gastrointestinal (Nausea) |
|  | Headache |
|  | Malaise |
|  | Myalgia |
|  | Fatigue |
|  | Joint pain |

### Unsolicited adverse events

Unsolicited symptoms are adverse events reported by the subjects which are different from those solicited or solicited symptoms which begin after the seven-day follow-up period for solicited symptoms. Space on the diary cards/source documents and CRF will be allocated for the recording of unsolicited symptoms. Should any systemic (general) signs/symptoms be reported, their relationship with the study vaccine will be assessed by the Investigator and transcribed into the CRF, as described in section 8.3.

## Assessment of intensity

Intensity of the solicited adverse events should be assessed as described in Tables 5-7. Grading of hematological and biochemical laboratory adverse events are in Table 8-9.

Table 5: Intensity grading of local solicited adverse events

| **Adverse event** | **Intensity grade** | **Intensity** |
| --- | --- | --- |
| Pain at injection site | 0 | Absent |
|  | 1 | Mild pain not interfering with function |
|  | 2 | Moderate pain; pain interfering with function but not interfering with activities of daily living (ADL) |
|  | 3 | Severe Pain; pain that severely interferes with ADL |
| Erythema at injection site | | Record greatest surface diameter in mm (see Table 9 ) |
| Swelling at injection site | | Record greatest surface diameter in mm (see Table 9 ) |

Intensity of the general adverse events should be assessed as described in Table 8.

Table 6: Intensity grading of general solicited adverse events

| **Adverse event** | **Intensity grade** | **Intensity** |
| --- | --- | --- |
| Fever (oral temperature ≥ 37.5°C or 99.5°F). | | Record oral temperature in °C/°F (see Table ) |
| Headache | 0 | Normal |
|  | 1 | Headache which is easily tolerated |
|  | 2 | Headache that interferes with normal activity |
|  | 3 | Headache that prevents normal activity |
| Fatigue | 0 | Normal |
|  | 1 | Fatigue which is easily tolerated |
|  | 2 | Fatigue that interferes with normal activity |
|  | 3 | Fatigue that prevents normal activity |
| Gastrointestinal | 0 | Normal |
| (*Nausea)* | 1 | GI symptoms which are easily tolerated |
|  | 2 | GI symptoms that interfere with normal activity |
|  | 3 | GI symptoms that prevent normal activity |
| Malaise | 0 | Normal |
|  | 1 | Malaise which is easily tolerated |
|  | 2 | Malaise that interferes with normal activity |
|  | 3 | Malaise that prevents normal activity |
| Myalgia | 0 | Normal |
|  | 1 | Myalgia which is easily tolerated |
|  | 2 | Myalgia that interferes with normal activity |
|  | 3 | Myalgia that prevents normal activity |
| Joint pain | 0 | Normal |
| (> 1 joint) | 1 | Joint pain which is easily tolerated |
|  | 2 | Joint pain that interferes with normal activity |
|  | 3 | Joint pain that prevents normal activity |

Table 7: Intensity grading assigned

| Erythema at injection site | 0 | 0 mm |
| --- | --- | --- |
|  | 1 | > 0 - 20 mm |
|  | 2 | > 20 - 50 mm |
|  | 3 | > 50 mm |
| Swelling at injection site | 0 | 0 mm |
|  | 1 | > 0 - 20 mm |
|  | 2 | > 20 - 50 mm |
|  | 3 | > 50 mm |
| Fever | 0 | < 37.5°C or 99.5°F |
|  | 1 | 37.5 - 38.0°C or 99.5 - 100.4 °F |
|  | 2 | > 38.0 to 39.5°C or 100.4 - 102.2°F |
|  | 3 | > 39.5°C or 102.2°F |

For all other adverse events, maximum intensity should be assigned to one of the following categories:

| 0 | = | No adverse event |
| --- | --- | --- |
| 1 | = | An adverse event that is easily tolerated by the subject, causing minimal discomfort and not interfering with everyday activities. |
| 2 | = | An adverse event that is sufficiently discomforting to interfere with normal everyday activities. |
| 3 | = | An adverse event that prevents normal, everyday activities i.e. prevents attendance at work and necessitates the administration of corrective therapy. |

Table 8: Serum chemistry reference ranges and critical values (Part B, Kenya)

| **Chemistry:** | **Gender** | **Reference Ranges** | **Critical Low** | **Critical High** |
| --- | --- | --- | --- | --- |
| Creatinine (mg/dL): | Male | 0.42 - 1.50 | N/A | >10 |
| (µmol/L): |  | 37 – 133 | N/A | >884 |
| (mg/dL): | Female | 0.17 - 1.17 | N/A | >10 |
| (µmol/L): |  | 15 – 103 | N/A | >884 |
| BUN (mg/dL): |  | 10.0 – 20 | N/A | >120 |
| (µmol/L): |  | 3.6 - 7.1 | N/A | >42.8 |
| ALT (U / L): |  | 0 – 35.0 | N/A | >75.0 |
| AST (U / L): |  | 0 – 35.0 | N/A | >80.0 |
| ALP (U / L): |  | 98 - 279 | N/A | > 800 |
| T - Bilirubin (mg/dL): |  | 0.3 - 1.0 | N/A | >15.0 |
| (µmol/L): |  | 5.0 – 17 | N/A | >257 |
| Sodium (µmol/L): |  | 136 – 145 | <120 | >160 |
| Potassium (µmol/L): |  | 3.5 - 5.0 | <2.8 | >6.5 |
| Bicarbonate (µmol/L): |  | 21 – 30 | <11.0 | >40.0 |
| Chloride (µmol/L): |  | 98 – 106 | <70.0 | >130 |
| Glucose (mg/L): |  | 75 – 115 | <50 | >400 |
| Lactate (mmol/L |  | 0.6 - 1.7 | N/A | >3.4 |
| Total Protein (g / L): |  | 55 – 80 |  |  |
|  |  |  |  |  |
| *Note: The identified values apply to both genders unless otherwise noted* | | | | |

Table 9: Hematology reference ranges and critical values (Part B, Kenya)

| **Hematology:** |  |  |  |  |
| --- | --- | --- | --- | --- |
|  | **Gender** | **Reference Ranges** | **Critical Low** | **Critical High** |
| WBC (x 10^3^ / µL): |  | 3.8 - 10.8 | <2.0 | >40.0 |
| RBC (x 10^6^ / µL): |  | 4.15 - 4.9 | N/A | N/A |
| HGB (g / dL): | Male | 13 – 18 | <8.0 | >20.0 |
|  | Female | 12.0 -16.0 | <7.0 | >20.0 |
| HCT (%): | Male | 42 – 52 | <24 % | >60% |
|  | Female | 37 – 48 | <21.0% | >50% |
| MCV (fL): |  | 86.0 - 98.0 | N/A | N/A |
| MCH (pg): |  | 28.0 - 33.0 | N/A | N/A |
| MCHC (g/dL): |  | 32.0 - 36.0 | N/A | N/A |
| RDW |  | 13.0 - 15.0 | N/A | N/A |
| Platelets (x 10^3^ / µL): |  | 130 – 400 | <81.0 | >1000.0 |
| LY ABS ( (x 10^3^ / µL): |  | 0.85 - 4.1 | N/A | N/A |
|  |  |  |  |  |
| *Note: The identified values apply to both genders unless otherwise noted* | | | | |

## Assessment of causality

Every effort should be made by the investigator to explain each adverse event and assess its causal relationship, if any, to administration of the study vaccine(s).

The degree of certainty with which an adverse event can be attributed to administration of the study vaccine(s) (or alternative causes, e.g. natural history of the underlying diseases, concomitant therapy, etc.) will be determined by how well the event can be understood in terms of one or more of the following:

- Reaction of similar nature having previously been observed with this type of vaccine and/or formulation.
- The event having often been reported in literature for similar types of vaccines.
- The event being temporally associated with vaccination or reproduced on re-vaccination.

All solicited local (injection site) reactions will be considered causally related to vaccination. Causality of all other adverse events should be assessed by the investigator using the following method:

In your opinion, did the vaccine(s) possibly contribute to the adverse event?

| NO | : | The adverse event is not causally related to administration of the study vaccine(s). There are other, more likely causes and administration of the study vaccine(s) is not suspected to have contributed to the adverse event. |
| --- | --- | --- |
| YES | : | There is a reasonable possibility that the vaccine contributed to the adverse event. |

Non-serious and serious adverse events will be evaluated as two distinct events given their different medical nature. If an event meets the criteria to be determined “serious” (see Section 8.5 for definition of serious adverse event), it will be examined by the investigator to the extent to be able to determine ALL contributing factors applicable to each serious adverse event.

Clinical laboratory parameters and other abnormal assessments qualifying as adverse events and serious adverse events

Abnormal laboratory findings (e.g., clinical chemistry, hematology, urinalysis) or other abnormal assessments (e.g. blood film) that are judged by the investigator to be clinically significant will be recorded as AEs or SAEs if they meet the definition of an AE, as defined in Section 8.1 or SAE, as defined in Section 8.5. Clinically significant abnormal laboratory findings or other abnormal assessments that are detected during the study or are present at baseline and significantly worsen following the start of the study will be reported as AEs or SAEs. However, clinically significant abnormal laboratory findings or other abnormal assessments that are associated with the disease being studied, unless judged by the investigator as more severe than expected for the subject’s condition, or that are present or detected at the start of the study and do not worsen, will not be reported as AEs or SAEs.

The investigator will exercise his or her medical and scientific judgment in deciding whether an abnormal laboratory finding or other abnormal assessment is clinically significant.

## Following-up of adverse events and assessment of outcome

Investigators should follow-up subjects with serious adverse events until the event has resolved, subsided, stabilized, disappeared, the event is otherwise explained, or the subject/patient is lost to follow-up; or, in the case of non-serious adverse events, the subject/patient completes the study. Clinically significant laboratory abnormalities, as well as any adverse event, will be followed up until they have returned to normal, or a satisfactory explanation has been provided. Reports relative to the subsequent course of an adverse event noted for any subject must be submitted to DHSP/WRAIR IRB.

Outcome should be assessed as:

1 = Recovered

2 = Recovered with sequelae

3 = Ongoing at subject study conclusion (active phase)

4 = Died

5 = Unknown

## Serious adverse events

### Definition of a serious adverse event

A serious adverse event (SAE) is any untoward medical occurrence that:

1. results in death,

b. is a life-threatening adverse drug experience,

c. requires hospitalization or prolongation of existing hospitalization,

d. results in a persistent or significant disability/incapacity, or

e. is a congenital anomaly/birth defect in the offspring of a study volunteer.

Medical or scientific judgment should be exercised in deciding whether reporting is appropriate in other situations, such as important medical events that may not be immediately life-threatening or result in death or hospitalization but may jeopardize the subject or may require medical or surgical intervention to prevent one of the other outcomes listed in the above definition. These should also be considered serious.

An unexpected or unanticipated adverse event is any adverse drug/test article experience, the specificity or severity of which is not consistent with the current investigator brochure or general investigational plan.

### Reporting serious adverse events

Serious Adverse Events will be reported in a prospective manner during the period starting from the day of administration of the first dose of study vaccine to each subject and ending with the last study. Unanticipated problems involving risk to volunteers or others, serious adverse events related to participation in the study and all volunteer deaths should be promptly reported by the investigator to the sponsor, KEMRI IRB, GSK Biologicals’ Clinical Safety Physician at Tel :+32 2 656 8850, Fax: +32 2 656 51 16 or +32 2 656 80 09; or mobile phones for 7/7 day availability: +32 477 404 713; e-mail: [rix.ct-safety-vac@gskbio.com](mailto:rix.ct-safety-vac@gskbio.com) or Head Safety Evaluation and Risk Management, Adult/Adolescent/Emerging Diseases at phone : +32472 906 600, or Head of Safety Evaluation and Risk Management, Pediatric, at mobile phone: +32 474 53 48 68(24/24 hour and 7/7 day availability) and to WRAIR IRB by phone (301 319-9940), by email (WRAIRDHSP@amedd.army.mil), or by facsimile (301-319-9961) to the Division of Human Subjects Protection, Walter Reed Army Institute of Research, 503 Robert Grant Ave., RM 1W30, Silver Spring, Maryland, 20910-7500. WRAIR IRB will report to USAMRMC ORP/HRPO as per SOP UWZ-C-636 within 24 hours (one calendar day) of his/her becoming aware of the event. Such events should also be reported in a similar method to the U.S. Army Medical Research and Materiel Command’s Human Subjects Research Review Board (HSRRB) by phone (310-619-2165) by email (hsrrb@det.amedd.army.mil) or by facsimile (301-619-7803) and USAMMDA Regulatory Affairs Office (telephone 301-619-0317, facsimile 301-619-0197 or via email usamrmcregulatoryaffairs@amedd.army.mil ). The investigator will document all available information regarding the serious adverse event in the Serious Adverse Event pages contained in the individual case report form and FAX to the GlaxoSmithKline Contact for Serious Adverse Event Reporting.

A complete written report should follow the initial notification within three working days.  In addition to the methods above, the complete report can be sent to the Director, Division of Human Subjects Protection, Walter Reed Army Institute of Research, 503 Robert Grant Ave., Silver Spring, MD 20910-7500 Room 1W30 and the U.S. Army Medical Research and Materiel Command, ATTN:  MCMR-ZB-P, 504 Scott Street, Fort Detrick, Maryland 21702-5012

The investigator should not wait to receive additional information to fully document the event before notifying GlaxoSmithKline Biologicals, or the USAMRMC Office of Research Protection (MRMCORP) and the USAMMDA Product Manager of a serious adverse event. This initial notification should give as minimum, sufficient information to permit identification of:

- The reporter
- The subject initials, identification number, date of birth, gender and ethnicity
- Study test article
- Date of administration
- Adverse events (Date of onset, signs/symptoms and severity)
- Action taken
- Concomitant medications (dose, route, duration)

In the event of a death or a serious adverse event determined by the investigator to be related to vaccination, receipt of the fax must be confirmed by a telephone call. The fax report should be followed by a full written report using either the GSK Biologicals Serious Adverse Events form or the US FDA MedWatch form, detailing relevant aspects of the adverse events in question. It should include copies of relevant hospital case records, autopsy reports, and other documents where applicable.

In the event that a serious adverse events determined by the investigator to be related to vaccination are detected following any vaccination in any of the vaccine groups, no further vaccinations will be administered until a written report has been submitted to KEMRI, the WRAIR IRB, HSRRB, USAMMDA Product Manager, and the FDA, and the investigators have conferred with the GSK, and WRAIR and/or USAMRU-K Medical Monitors.

If an instance of congenital abnormality in offspring is brought to the attention of the investigator at any time after cessation of study vaccine AND is suspected by the investigator to be related to study vaccine, it should be reported to the Study Contacts for Serious Adverse Event Reporting.

The local medical monitor is required to review all unanticipated problems involving risk to subjects or others, serious adverse events, and all subject deaths associated with the protocol and provide an unbiased written report of the event.  At a minimum the local medical monitor should comment on the outcomes of the event or problem and, in the case of a serious adverse event or death, comment on the relationship to participation in the study.  The medical monitor should also indicate whether he/she concurs with the details of the report provided by the study investigator.  Reports for events determined by either the investigator or medical monitor to be possibly or definitely related to participation and reports of events resulting in death should be promptly forwarded to KEMRI and the WRAIR IRB.

Any suspensions (to include continuing review lapses), clinical holds (voluntary or involuntary), or terminations of this research by an IRB, the institution, the Sponsor, or regulatory agencies will be promptly to the KEMRI ERC and WRAIR IRB. WRAIR IRB will report to USAMRMC ORP/HRPO as per SOP UWZ-C-636.

All information should be sent promptly to the contacts listed below:

| **Study Contact for Reporting Serious Adverse Events**  **DHSP/WRAIR IRB**  **Division of Human Subjects Protection**  Walter Reed Army Institute of Research  503 Robert Grant Ave  Silver Spring, MD 20910  Room 1W30  Tel: 301-319-9940  Fax: 301-319-9961  WRAIRDHSP@amedd.army.mil |
| --- |
| **USAMRMC Human Research Protections Office(HRPO)/HSRRB**  US Army Medical Research and Materiel Command  ATTN: MCMR-ORP-HR  504 Scott Street  Fort Detrick, MD 21702-5012  Tel: 301-619-2165/6  Fax: 301-619-7803/789  [hsrrb@amedd.army.mil](mailto:hsrrb@amedd.army.mil) |
| **USAMMDA**  Director,RegulatoryAffairs 1430VeteransDrive Ft.Detrick,MD21702-9232 Tel:301-619-0317 Fax: 301-619-0197 |
| **KEMRI Ethics Review Committee (ERC)**  Dr Monique Wasuna  Director, Centre for Clinical Research,  Kenya Medical Research Institute (KEMRI)  Nairobi, Kenya.  Tel: +254 20 272 254 Email: [erc@Kemri.org](mailto:erc@Kemri.org) |
| **Study Contact at GlaxoSmithKline for Reporting Serious Adverse Events** |
| **Primary Study contacts at GSK Biologicals** |
| GSK Biologicals Clinical Safety Physician  Tel: +32 2 656 8850  **Fax: +32 2 656 51 16 or +32 2 656 80 09**  Mobile phones for 7/7 day availability:  +32 477 404 713  (Head Safety Evaluation and Risk Management, Adult/Adolescent/Emerging  Diseases)  +32472 906 600  (Head Safety Evaluation and Risk Management, Pediatric)  Back-up mobile phone contact:  +32 474 53 48 68  24/24 hour and 7/7 day availability |
| **Back-up Study Contacts at GlaxoSmithKline for Reporting Serious Adverse Events** |
| Medical Monitor/ Clinical Development Manager  Olivier Godeaux, MD, MPH  GlaxoSmithKline Biologicals  Rue de l'Institut, 89  1330 Rixensart  Belgium  Tel. office: +32-2-656 6910  Fax: +32-2-656 8044  email: [Olivier.godeaux@gskbio.com](mailto:Olivier.godeaux@gskbio.com)  Evi de Ruymaeker  Rue de I’ Institut, 89  1330 Rixensart, Belgium  Tel : 32-2-656 3760  Fax : 32-2-656 8044  **Emailevi.de.ruymaeker@gskbio.com** |
|  |
|  |
| **Independent Medical Monitor (Part B)**  Dr Amos Evans Otedo  Consultant Physician/Gastroentorologist  Box 40100-4685, Kisumu Kenya  Tel: +254 722 866 052  Email: [otedoamos@yahoo.com](mailto:otedoamos@yahoo.com) |
|  |

### Pregnancy

Subjects who become pregnant during the study period must not receive additional doses of vaccine, but may continue other study procedures to include blood draws for immunogenicity and safety and review of symptoms/physical exams for reactogenicity data, at the discretion of the primary investigator. Subjects will be instructed to notify the investigator if it is determined after completion of the study that they became pregnant either during the study or within 30 days of the study. Although not considered an adverse event, pregnancy will be reported in the same way as a serious adverse event (see Section 8.5.2).

A pregnancy will be followed to term. Any premature terminations will be reported, and the health status of the mother and child including date of delivery and the child’s gender and weight will be reported to DHSP/ WRAIR IRB, HSRRB, USAMMDA, KEMRI and GSK Biologicals after delivery.

### Treatment of adverse events

The PI, in conjunction with the medical monitor, will ensure each volunteer receives appropriate treatment of any adverse event related to the vaccine.

# SUBJECT COMPLETION AND DROPOUT

## Definition

A volunteer who has completed the study is one who receives all three doses of the vaccine and has had the required safety and immunogenicity evaluations. A dropout is anyone who has not completed the study. Reasons for dropout are listed in Section 9.3 below. Subject compliance for test article administration will not be an issue because the vaccine will be administered by study staff. The subject will be reminded by study staff and/or investigators about follow-up visits as outlined in the study schedule. If a volunteer is not compliant with a study visit, the study staff will actively attempt to contact the volunteer and document these attempts in the volunteer file.

## Procedures for handling dropouts

Investigators will make an attempt to contact those subjects who do not return for scheduled visits or follow-up. Information gathered will be described on the Study Conclusion page of the CRF and on Medication/Adverse event forms.

## 9.3 Reasons for dropout

It will be specified on the Study Conclusion page of the CRF, which of the following possible reasons were responsible for dropout of the subject from the study:

- Serious adverse event
- Non-serious adverse event
- Protocol violation (specify)
- Consent withdrawal, not due to an adverse event
- Migrated/moved from the study area
- Lost to follow-up
- Other (specify)

# DATA EVALUATION: CRITERIA FOR EVALUATION OF OBJECTIVES

## Study Objectives

# Primary Objective

Assess the safety and reactogenicity of FMP010/AS01B candidate malaria vaccine in healthy malaria-naïve adults from the US and malaria experienced adults in Kenya.

# Secondary Objective

Measure and compare, by Growth Inhibition Assay (GIA), the functional humoral immune responses induced in malaria experienced adults in Kenya to that induced in healthy malaria-naïve adults from the US by FMP010/AS01B and FMP1/AS02 A.

**Tertiary Objectives**

Measure and compare, by enzyme-linked immunoabsorbent assay (ELISA), the humoral immune responses induced by vaccine candidate FMP010/AS01B in malaria experienced adults in Kenya to those induced in healthy malaria-naïve adults from the US.

## Study Endpoints

**Co-primary endpoints**

- Occurrence and intensity of solicited symptoms on day of vaccination plus days 1, 2, 3 and 7 and after each vaccination
- Occurrence and intensity of unsolicited symptoms over a 30-day follow-up period (day of vaccination plus 29 subsequent days) after each vaccination
- Occurrence of serious adverse events (SAEs), defined IAW 21 CFR 312.32(a), during the study period.

**Secondary endpoints**

Functionality of antibodies elicited as measured by percent parasite growth inhibition determined by GIA against FVO and 3D7 *P. falciparum* strains at specified time points

**Tertiary endpoints**

Titers of anti-FMP010 and anti-FMP1 antibodies as determined by ELISA at specified time points

- To whole MSP1_42_ antigen of 3D7 and FVO allele

● To subunit antigens and selected peptides of MSP1_42_ molecule

## Exploratory Endpoints

These assays may provide additional information on the immunogenicity of the vaccine candidate FMP010/AS01B. The assays will be completed at both WRAIR and USAMRU-K if, after performing endpoint assays, sufficient sample remains and study priorities permit.

- Detection of *P. falciparum* antigens by HRP-2 and pLDH ELISAs
- Growth Inhibition Assays (GIAs) in immunized malaria-experienced Kenyan adults
- Titers of anti-FMP010 and FMP1 antibodies as determined by bead-based flow cytometric assays (*Luminex™*) at specified time points
- Anti-merozoite immunofluorescence (IFA) assays to assess ability of antibodies to recognize MSP1 molecule on infected erythrocytes
- Processing inhibition assay (PIA) to assess ability of antibodies to inhibit processing of MSP1_42_ molecule to MSP1_33_ and MSP1_19_
- Cytokine bead-based flow cytometric assay (*Luminex*) to assess cytokine production in responses to vaccine administration
- Assays such as ELISPOT and intracellular cytokine staining to assess cellular immune responses

##

## Study data sets to be evaluated

**Total Vaccinated Cohort**

The Total Vaccinated Cohort will include all vaccinated subjects for whom data are available. Thus, the total analysis of safety will include all subjects with at least one vaccine administration documented and the total analysis of immunogenicity will include vaccinated subjects for whom data concerning immunogenicity endpoint measures are available. The Total Vaccinated Cohort analysis will be performed per treatment actually administered.

**Protocol defined or According To Protocol (ATP) cohort for analysis of safety**

The ATP cohort for analysis of safety/reactogenicity will include all subjects who meet all eligibility criteria and comply with all procedures defined in the protocol and will include subjects for whom data are available for the analysis of safety/reactogenicity.

The ATP cohort for analysis of safety will include all evaluable subjects

- who have received at least one dose of study vaccine according to their random assignment;
- who have sufficient data to perform an analysis of safety (at least one vaccine dose with safety follow-up);
- who have not received a vaccine not specified or forbidden in the protocol and for whom elimination criteria were not applied; and,
- for whom the randomization code has not been broken (as applicable).

**Protocol defined or ATP cohort for analysis of immunogenicity**

The ATP cohort for analysis of immunogenicity will include all subjects for whom differential treatment effect on immunogenicity is likely (i.e., those meeting all eligibility criteria, complying with the procedures defined in the protocol) and will include subjects for whom assay results are available for antibodies against at least one study vaccine antigen component after vaccination.

##

## Estimated Sample Size and Sample Size Justification

This study is designed to assess the initial safety and immunogenicity of a new candidate malaria vaccine. The sample size is in keeping with small numbers of volunteers traditionally included in initial Phase I studies. Comparative statistics will be performed but will have low power to detect other than very large differences between groups therefore the most likely result will be a descriptive analysis of safety.

## Final Analyses

###

### Analysis of demographics

Demographic characteristics (age, sex, and race) of each study cohort will be tabulated. The mean age (plus range and standard deviation) by sex of the enrolled subjects, as a whole, and per group, will be calculated.

### Analysis of safety

**Analysis of reactogenicity**

Reactogenicity analyses will be analyzed as follows.

- The overall percentage of subjects with at least one local adverse event (solicited and unsolicited), with at least one general adverse event (solicited and unsolicited), during the seven-day follow-up period after vaccination will be tabulated.
- The incidence, intensity and relationship of individual solicited symptoms over the 7-day follow‑up period will be calculated per group and vaccine dose.
- The number of subjects with at least one report of an unsolicited adverse event, classified by WHO Preferred terms or by the Medical Dictionary for Regulatory Activities (MedDRA) if available and reported after the Vaccination Phase, will be tabulated per group and vaccine dose. The intensity and relationship to vaccination of the unsolicited symptoms reported will also be assessed. Serious adverse events are expected to be rare, but where observed will be described.

**Clinical Laboratory parameters**

Hematological and biochemical laboratory parameters will be measured at specific time points during the study. Clinically relevant abnormal values will be tabulated.

### Analysis of immunogenicity

**Growth Inhibition Assays (GIA)**

Growth inhibition assays (GIA) will be reported in a tabular manner as the percent growth inhibition seen at a given serum dilution compared with the same dilution of pre-immune serum from the same volunteer. GIA will be performed in all volunteers pre- and post-vaccination following standard procedures. An individual serum will be considered to be GIA positive, if compared with paired pre-immune control serum, the growth inhibition value is greater than 20%.

The criteria for FMP010/AS01B progression to Phase 2b and future development are based on establishing statistically significant differences in the ability of the FMP010/AS01B and FMP1/AS02A vaccines to induce biologically relevant antibody responses against homologous parasites *in vitro*. FMP010/AS01B should induce inhibitory antibody responses above the established cutoff level, 20% inhibition (20% serum pLDH GIA assay) in a minimum number of volunteers against homologous parasites, *P. falciparum* FVO (i.e. 10 out 15, thus 67% seropositivity).

| **Criteria** | **FMP010/AS01B** | **FMP1.AS02A** |
| --- | --- | --- |
| Seropositivity | 10 out of 15, 67% | 2 out of 5, 40% |
| Mean Inhibition (%)  Homologous parasites | 30 ± 5 | 15 ± 3 |

**Anti-FMP010 antibody responses**

Anti-FMP010 antibody concentrations will be summarized by Geometric Mean Titers (GMTs), with the 95% CI, at each time point at which blood samples are taken for serology.

Peak responses (Day 70) will be compared by Student’s t test on data normalized by log transformation to ascertain presence or absence of significant dose response difference. An adjustment to the statistical method will be made if multiple comparisons are warranted

# ADMINISTRATIVE MATTERS

To comply with Good Clinical Practice important administrative obligations relating to investigator responsibilities, monitoring, archiving data, audits, confidentiality and publications must be fulfilled. See Appendix B for details.

# REFERENCES

1. Diggs, C.L., Ballou, W.R., and L.H. Miller. 1993. The major merozoite surface protein as malaria vaccine target. Parasitol. Today 9: 300-302.
2. Good, M.F., and D.L. Doolan. 1999. Immune effector mechanisms in malaria. Curr. Opin. Immunol. 11:412.
3. Hui, G.S., W.L. Gosnell, S.E. Case, D. Hashiro, C. Nikaido, A. Hashimoto, and D.C. Kaslow. 1994. Immunogenicity of the C-terminal 19-kDa fragment of the *Plasmodium falciparum* merozoite surface protein 1 (MSP1), YMSP1_19_ expressed in *S. cerevisiae*. J. Immunol 153:2544.
4. Sachs J., and P. Malaney. 2002. The economic and social burden of malaria. Nature 415; 681-685.
5. Haldar, K., M. A. Ferguson, and G. A. Cross. 1985. Acylation of a *Plasmodium falciparum* merozoite surface antigen via sn- 1,2-diacyl glycerol. J Biol Chem 260:4969-74.
6. Lyon, J. A., J. D. Haynes, C. L. Diggs, J. D. Chulay, C. G. Haidaris, and J. Pratt-Rossiter. 1987. Monoclonal antibody characterization of the 195-kilodalton major surface glycoprotein of *Plasmodium falciparum* malaria schizonts and merozoites: identification of additional processed products and a serotype-restricted repetitive epitope. J Immunol 138:895-901.
7. McBride, J. S., and H. G. Heidrich. 1987. Fragments of the polymorphic Mr 185,000 glycoprotein from the surface of isolated *Plasmodium falciparum* merozoites form an antigenic complex. Mol Biochem Parasitol 23:71-84.
8. Holder, A. A., and R. R. Freeman. 1984. Protective antigens of rodent and human bloodstage malaria. Philos Trans R Soc Lond B Biol Sci 307:171-7.
9. Holder, A. A., J. S. Sandhu, Y. Hillman, L. S. Davey, S. C. Nicholls, H. Cooper, and M. J. Lockyer. 1987. Processing of the precursor to the major merozoite surface antigens of *Plasmodium falciparum*. Parasitology 94:199-208.
10. Morgan, W. D., B. Birdsall, T. A. Frenkiel, M. G. Gradwell, P. A. Burghaus, S. E. Syed, C. Uthaipibull, A. A. Holder, and J. Feeney. 1999. Solution structure of an EGF module pair from the *Plasmodium falciparum* merozoite surface protein 1. J Mol Biol 289:113-22.
11. Blackman, M. J., and A. A. Holder. 1992. Secondary processing of the *Plasmodium falciparum* merozoite surface protein-1 (MSP1) by a calcium-dependent membrane-bound serine protease: shedding of MSP1_33_ as a noncovalently associated complex with other fragments of the MSP1. Mol Biochem Parasitol 50:307-15.
12. Blackman, M. J., I. T. Ling, S. C. Nicholls, and A. A. Holder. 1991. Proteolytic processing of the *Plasmodium falciparum* merozoite surface protein-1 produces a membrane-bound fragment containing two epidermal growth factor-like domains. Mol Biochem Parasitol 49:29-33.
13. Miller, L. H., T. Roberts, M. Shahabuddin, and T. F. McCutchan. 1993. Analysis of sequence diversity in the *Plasmodium falciparum* merozoite surface protein-1 (MSP-1). Mol Biochem Parasitol 59:1-14.
14. Renia, L., I. T. Ling, M. Marussig, F. Miltgen, A. A. Holder, and D. Mazier. 1997. Immunization with a recombinant C-terminal fragment of *Plasmodium yoelii* merozoite surface protein 1 protects mice against homologous but not heterologous *P. yoelii* sporozoite challenge. Infect Immun 65:4419-23.
15. Blackman, M. J., and A. A. Holder. 1992. Secondary processing of the *Plasmodium falciparum* merozoite surface protein-1 (MSP1) by a calcium-dependent membrane-bound serine protease: shedding of MSP1_33_ as a noncovalently associated complex with other fragments of the MSP1. Mol Biochem Parasitol 50:307-15.
16. Chappel, J. A., and A. A. Holder. 1993. Monoclonal antibodies that inhibit *Plasmodium falciparum* invasion in vitro recognise the first growth factor-like domain of merozoite surface protein-1. Mol Biochem Parasitol 60:303-11.
17. Ling, I. T., S. A. Ogun, and A. A. Holder. 1994. Immunization against malaria with a recombinant protein. Parasite Immunol 16:63-7.
18. Majarian, W. R., T. M. Daly, W. P. Weidanz, and C. A. Long. 1984. Passive immunization against murine malaria with an IgG3 monoclonal antibody. J. Immunol. 132:3131-3137.
19. Blackman, M. J., T. J. Scott-Finnigan, S. Shai, and A. A. Holder. 1994. Antibodies inhibit the protease-mediated processing of a malaria merozoite surface protein. J Exp Med 180:389-93.
20. O'Donnell, R. A., T. F. de Koning-Ward, R. A. Burt, M. Bockarie, J. C. Reeder, A. F. Cowman, and B. S. Crabb. 2001. Antibodies against merozoite surface protein (MSP)-1(19) are a major component of the invasion-inhibitory response in individuals immune to malaria. J Exp Med 193:1403-12.
21. Egan, A. F., P. Burghaus, P. Druilhe, A. A. Holder, and E. M. Riley. 1999. Human antibodies to the 19kDa C-terminal fragment of *Plasmodium falciparum* merozoite surface protein 1 inhibit parasite growth *in vitro*. Parasite Immunol 21:133-9.
22. Angov, E., B. M. Aufiero, A. M. Turgeon, M. Van Handenhove, C. F. Ockenhouse, K. E. Kester, D. S. Walsh, J. S. McBride, M. C. Dubois, J. Cohen, J. D. Haynes, K. H. Eckels, D. G. Heppner, W. R. Ballou, C. L. Diggs, and J. A. Lyon. 2003. Development and pre-clinical analysis of a *Plasmodium falciparum* Merozoite Surface Protein-1(42) malaria vaccine. Mol Biochem Parasitol 128:195-204.
23. Chang, S. P., H. L. Gibson, C. T. Lee-Ng, P. J. Barr, and G. S. Hui. 1992. A carboxyl-terminal fragment of *Plasmodium falciparum* gp195 expressed by a recombinant baculovirus induces antibodies that completely inhibit parasite growth. J Immunol 149:548-55.
24. Singh, S., M. C. Kennedy, C. A. Long, A. J. Saul, L. H. Miller, and A. W. Stowers. 2003. Biochemical and immunological characterization of bacterially expressed and refolded *Plasmodium falciparum* 42-kilodalton C-terminal merozoite surface protein 1. Infect Immun 71:6766-74.
25. Chang, S. P., S. E. Case, W. L. Gosnell, A. Hashimoto, K. J. Kramer, L. Q. Tam, C. Q. Hashiro, C. M. Nikaido, H. L. Gibson, C. T. Lee-Ng, P. J. Barr, B. T. Yokota, and G. S. Hut. 1996. A recombinant baculovirus 42-kilodalton C-terminal fragment of *Plasmodium falciparum* merozoite surface protein 1 protects *Aotus* monkeys against malaria. Infect Immun 64:253-61.
26. Stowers, A. W., L. H. Chen Lh, Y. Zhang, M. C. Kennedy, L. Zou, L. Lambert, T. J. Rice, D. C. Kaslow, A. Saul, C. A. Long, H. Meade, and L. H. Miller. 2002. A recombinant vaccine expressed in the milk of transgenic mice protects *Aotus* monkeys from a lethal challenge with *Plasmodium falciparum*. Proc Natl Acad Sci U S A 99:339-44.
27. Stowers, A. W., V. Cioce, R. L. Shimp, M. Lawson, G. Hui, O. Muratova, D. C. Kaslow, R. Robinson, C. A. Long, and L. H. Miller. 2001. Efficacy of two alternate vaccines based on *Plasmodium falciparum* merozoite surface protein 1 in an *Aotus* challenge trial. Infect Immun 69:1536-46.
28. Darko, C. A, Angov, E., Collins, W. E., Bergmann-Leitner, E.S., Girouard, A.S., Hitt, S.L., McBride, J.S., Diggs, C.L, Holder, A.A., Long, C.A., Barnwell, J.W., and J. A. Lyon. 2005. Clinical grade *Plasmodium falciparum* FVO MSP1_42_ Expressed in *Escherichia coli* protects *Aotus nancymai* against homologous erythrocytic-stage challenge. Infect Immun 73: In press
29. Stewart VA, McGrath SM, Walsh DS, Davis S, Hess AS, Ware LA, Kester KE, Cummings JF, Burge JR, Voss G, Delchambre M, Garcon N, Tang DB, Cohen JD, Heppner DG. 2006. Pre-clinical evaluation of new adjuvant formulations to improve the immunogenicity of the malaria vaccine RTS,S/AS02A. Vaccine. 24:6483-6492.
30. Angov, E., Aufiero, B.A., Van Handenhove, M., Ockenhouse, C.F., Kester, K., Walsh, D., McBride, J., Heppner, D.G., Ballou, W.R., Diggs, C., and J.A. Lyon. 2003. Process Development and Preclinical Analysis of a Recombinant P. falciparum Merozoite Surface Protein-1_42_ Malaria Vaccine. Mol Biochem. Parasitol. 128:195-204)
31. Ockenhouse CF, Angov EA, Kester KE, Diggs C, Soisson, Cummings JF, Stewart VA, Palmer DR, Mahajan B, Krzych U, Tornieporth N, Delchambre M, Vanhandehove M, Ofori-Anyinam O, Cohen J, Lyon JA, Heppner DG and MSP-1 Working Group. 2006. Phase 1 safety and immunogenicity trail of FMP1/AS02A, a *Plasmodium falciparum* MSP-1 asexual blood stage vaccine. Vaccine. 24:3009-3017.
32. Stoute JA, Gombe J, Withers MR, Siangla J, McKinney D, Onyango M, Cumings JF, Milman J, Tucker K, Soisson L, Stewart VA, Lyon JA, Angov EA, Leach A, Cohen J, Kester KE, Ockenhouse CF, Holland CA, Diggs CL, Wittes J, Heppner DG, the MSP-1 Working Group. 2006. Vaccine. 2006. 25(1):176-184.
33. Thera MA, Doumbo OK, Coulibaly D, Diallo DA, Sagara I, Dicko A, Diemert DJ, Heppner DG, Stewart VA, Angov EA, Soisson L, Leach A, Tucker K, Lyke KE, Plowe CV, for the Mali FMP1 Working Group. 2006. PLoS Clin Trials. 1(7):e34
34. Withers MR, McKinney D, Ogutu BR, Waitumbi JN, Milman JB, Apollo OJ, Allen OG, Tucker K, Soisson LA, Diggs C, Leach A, Wittes J, Dubovsky F, Stewart VA, Remich SA, Cohen J, Ballou WR, Holland CA, Lyon JA, Angov E, Stoute JA, Martin SK, Heppner DG, for the MSP-1 Malaria vaccine Working Group. 2006. PLoS Clin Trials. 2006. 1(7):e37.
35. [Nicholson KG](http://www.ncbi.nlm.nih.gov/sites/entrez?Db=pubmed&Cmd=Search&Term="Nicholson KG"%5BAuthor%5D&itool=EntrezSystem2.PEntrez.Pubmed.Pubmed_ResultsPanel.Pubmed_DiscoveryPanel.Pubmed_RVAbstractPlus), [Farrow PR](http://www.ncbi.nlm.nih.gov/sites/entrez?Db=pubmed&Cmd=Search&Term="Farrow PR"%5BAuthor%5D&itool=EntrezSystem2.PEntrez.Pubmed.Pubmed_ResultsPanel.Pubmed_DiscoveryPanel.Pubmed_RVAbstractPlus), [Bijok U](http://www.ncbi.nlm.nih.gov/sites/entrez?Db=pubmed&Cmd=Search&Term="Bijok U"%5BAuthor%5D&itool=EntrezSystem2.PEntrez.Pubmed.Pubmed_ResultsPanel.Pubmed_DiscoveryPanel.Pubmed_RVAbstractPlus), [Barth R](http://www.ncbi.nlm.nih.gov/sites/entrez?Db=pubmed&Cmd=Search&Term="Barth R"%5BAuthor%5D&itool=EntrezSystem2.PEntrez.Pubmed.Pubmed_ResultsPanel.Pubmed_DiscoveryPanel.Pubmed_RVAbstractPlus). Pre-exposure studies with purified chick embryo cell culture rabies vaccine and human diploid cell vaccine: serological and clinical responses in man. Vaccine. 1987 Sep;5(3):208-10

# Appendix A: World Medical Association Declaration of Helsinki

Recommendations guiding physicians

in biomedical research involving human subjects

Adopted by the 18th World Medical Assembly

Helsinki, Finland, June 1964

and amended by the

29th World Medical Assembly

Tokyo, Japan, October 1975

35th World Medical Assembly

Venice, Italy, October 1983

41st World Medical Assembly

Hong Kong, September 1989

and the

48th General Assembly

Somerset West, Republic of South Africa, October 1996

INTRODUCTION

It is the mission of the physician to safeguard the health of the people. His or her knowledge and conscience are dedicated to the fulfilment of this mission.

The Declaration of Geneva of the World Medical Association binds the physician with the words, "The health of my patient will be my first consideration," and the International Code of Medical Ethics declares that, "A physician shall act only in the patient's interest when providing medical care that might have the effect of weakening the physical and mental condition of the patient."

The purpose of biomedical research involving human subjects must be to improve diagnostic, therapeutic and prophylactic procedures and the understanding of the etiology and pathogenesis of disease.

In current medical practice most diagnostic, therapeutic or prophylactic procedures involve hazards. This applies especially to biomedical research.

Medical progress is based on research that ultimately must rest in part on experimentation involving human subjects.

In the field of biomedical research a fundamental distinction must be recognized between medical research in which the aim is essentially diagnostic or therapeutic for a patient, and medical research, the essential object of which is purely scientific and without implying direct diagnostic or therapeutic value to the person subjected to the research.

Special caution must be exercised in the conduct of research that may affect the environment, and the welfare of animals used for research must be respected.

Because it is essential that the results of laboratory experiments be applied to human beings to further scientific knowledge and to help suffering humanity, the World Medical Association has prepared the following recommendations as a guide to every physician in biomedical research involving human subjects. They should be kept under review in the future. It must be stressed that the standards as drafted are only a guide to physicians all over the world. Physicians are not relieved from criminal, civil and ethical responsibilities under the laws of their own countries.

I. BASIC PRINCIPLES

1. Biomedical research involving human subjects must conform to generally accepted scientific principles and should be based on adequately performed laboratory and animal experimentation and on a thorough knowledge of the scientific literature.
2. The design and performance of each experimental procedure involving human subjects should be clearly formulated in an experimental protocol that should be transmitted for consideration, comment and guidance to a specially appointed committee independent of the investigator and the sponsor provided that this independent committee is in conformity with the laws and regulations of the country in which the research experiment is performed.
3. Biomedical research involving human subjects should be conducted only by scientifically qualified persons and under the supervision of a clinically competent medical person. The responsibility for the human subject must always rest with a medically qualified person and never rest on the subject of research, even though the subject has given his or her consent.
4. Biomedical research involving human subjects cannot legitimately be carried out unless the importance of the objective is in proportion to the inherent risk to the subject.
5. Every biomedical research project involving human subjects should be preceded by careful assessment of predictable risks in comparison with foreseeable benefits to the subject or to others. Concern for the interests of the subject must always prevail over the interests of science and society.
6. The right of the research subject to safeguard his or her integrity must always be respected. Every precaution should be taken to respect the privacy of the subject and to minimize the impact of the study on the subject's physical and mental integrity and on the personality of the subject.
7. Physicians should abstain from engaging in research projects involving human subjects unless they are satisfied that the hazards involved are believed to be predictable. Physicians should cease any investigation if the hazards are found to outweigh the potential benefits.
8. In publication of the results of his or her research, the physician is obliged to preserve the accuracy of the results. Reports of experimentation not in accordance with the principles laid down in this Declaration should not be accepted for publication.
9. In any research on human beings, each potential subject must be adequately informed of the aims, methods, anticipated benefits and potential hazards of the study and the discomfort it may entail. He or she should be informed that he or she is at liberty to abstain from participation in the study and that he or she is free to withdraw his or her consent to participation at any time. The physician should then obtain the subject's freely‑given informed consent, preferably in writing.
10. When obtaining informed consent for the research project the physician should be particularly cautious if the subject is in a dependent relationship to him or her or may consent under duress. In that case the informed consent should be obtained by a physician who is not engaged in the investigation and who is completely independent of this official relationship.
11. In case of legal incompetence, informed consent should be obtained from the legal guardian in accordance with national legislation. Where physical or mental incapacity makes it impossible to obtain informed consent, or when the subject is a minor, permission from the responsible relative replaces that of the subject in accordance with national legislation.
12. Whenever the minor child is in fact able to give consent, the minor's consent must be obtained in addition to the consent of the minor's legal guardian.
13. The research protocol should always contain a statement of the ethical considerations involved and should indicate that the principles enunciated in the present Declaration are complied with.

II. MEDICAL RESEARCH COMBINED WITH PROFESSIONAL CARE
(Clinical research)

1. In the treatment of the sick person, the physician must be free to use a new diagnostic and therapeutic measure, if in his or her judgment it offers hope of saving life, re-establishing health or alleviating suffering.
2. The potential benefits, hazards and discomfort of a new method should be weighed against the advantages of the best current diagnostic and therapeutic methods.
3. In any medical study, every patient - including those of a control group, if any - should be assured of the best-proven diagnostic and therapeutic method. This does not exclude the use of inert placebo in studies where no proven diagnostic or therapeutic method exists.
4. The refusal of the patient to participate in a study must never interfere with the physician–patient relationship.
5. If the physician considers it essential not to obtain informed consent, the specific reasons for this proposal should be stated in the experimental protocol for transmission to the independent committee (I, 2).
6. The Physician can combine medical research with professional care, the objective being the acquisition of new medical knowledge, only to the extent that medical research is justified by its potential diagnostic or therapeutic value for the patient.

III. NON-THERAPEUTIC BIOMEDICAL RESEARCH INVOLVING HUMAN SUBJECTS (Non-clinical biomedical research)

1. In the purely scientific application of medical research carried out on a human being, it is the duty of the physician to remain the protector of the life and health of that person on whom biomedical research is being carried out.
2. The subjects should be volunteers ‑ either healthy persons or patients for whom the experimental design is not related to the patient's illness.
3. The investigator or the investigating team should discontinue the research if in his/her or their judgment it may, if continued, be harmful to the individual.
4. In research on man, the interest of science and society should never take precedence over considerations related to the well being of the subject.

# Appendix B: Administrative Matters

**I. Responsibilities of the Investigator IAW 21 CFR 312.60 and ICH E6, section 4**

- To ensure that he/she has sufficient time to conduct and complete the study and has adequate staff and appropriate facilities that are available for the duration of the study and to ensure that other studies do not divert essential subjects or facilities away from the study at hand.
- To submit an up-to-date curriculum vitae and other credentials (e.g. medical license number in the United States) to the sponsor and—where required—to relevant authorities.
- To acquire the normal ranges for laboratory tests performed locally and, if required by local regulations, obtain the Laboratory License or Certification.
- Additional laboratory assays may be performed on stored serum samples left over after protocol specified laboratory testing is completed. This may include clinical tests performed in the evaluation of a volunteer’s medical problem or additional malaria specific immunologic evaluations as they become available. In either case, the PI will inform the IRB of any non-protocol specified laboratory testing that may occur.
- To prepare and maintain adequate case histories designed to record observations and other data pertinent to the study.
- To conduct the study in compliance with the protocol and appendices.
- To cooperate with a representative of USAMMDA and GSK Biologicals in the monitoring process of the study and in resolution of queries about the data.

**II. Protocol Amendments and modifications**

- No changes to the study protocol will be allowed unless discussed in detail with the USAMMDA and GSK Biologicals' Clinical Development Manager and USAID and filed as an amendment/modification to this protocol.
- Any amendment/modification to the protocol will be adhered to by the participating center(s) and will apply to all subjects. WRAIR IRB approval of protocol amendments is required prior to implementation.

**All amendments/modifications will be submitted through the WRAIR Division of Human Subjects Protection for review by the WRAIR Scientific Review and Human Use Review Committee. KEMRI ERC will also review all protocol modifications. Review by the HSRRB will be required if the amendment/modification increases the risk to study participants.**

**III. Sponsor’s Termination of Study**

- USAMMDA and GSK Biologicals reserve the right to discontinue the clinical study at any time for medical or administrative reasons. When feasible, a 30-day written notification will be tendered.

**IV. Monitoring by USAMMDA/GSK BIOLOGICALS**

- Monitoring visits will be scheduled to take place before entry of the first subject, during the study at appropriate intervals and after the last subject has completed. It is anticipated that monitoring visits will occur at initiation of the study, periodically through the study and at study closeout.
- These visits are for the purpose of confirming that USAMMDA/GSK Biologicals sponsored studies are being conducted in compliance with the relevant Good Clinical Practice regulations/ guidelines, verifying adherence to the protocol and the completeness and exactness of data entered on the CRF and Vaccine Inventory Forms.
- The monitor will verify CRF entries by comparing them with the source data/documents that will be made available by the investigator for this purpose. The investigator must ensure provision of reasonable time, space and adequate qualified personnel for monitoring visits.

**V. Archiving of Data**

- The investigator/ institution will maintain all study documentation at the individual study site until at least 2 years after the last approval of a marketing application in an ICH region and until there are no pending or contemplated marketing applications in an ICH region or at least 2 years have elapsed since the formal discontinuation of the clinical development of the investigational product. These documents will be retained for a longer period however if required by the applicable regulatory requirements or by an agreement with the sponsor. It is the responsibility of the sponsor to inform the investigator/institution as to when these documents no longer need to be retained. The investigator/ institution will take measures to prevent accidental or premature destruction of these documents.
- Similarly, the sponsor-specific study documentation will be retained until at least 2 years after the last approval of a marketing application in an ICH region and until there are no pending or contemplated marketing applications in an ICH region or at least 2 years have elapsed since the formal discontinuation of clinical development of the investigational product. These documents will be retained for a longer period however if required by the applicable regulatory requirements or if needed by the sponsor. The sponsor will inform the investigator/institution in writing of the need for record retention and will notify the investigator/institution in writing when the study-related records are no longer needed.

**VI. Audits**

- For the purpose of compliance with Good Clinical Practice and Regulatory Agency Guidelines it may be necessary for USAMMDA (or other representative of MRMC), GSK,or the FDA to conduct a site audit. This may occur at any time from start to after conclusion of the study.
- When an investigator signs the protocol, he agrees to permit USAMMDA, GSK, and the FDA audits, providing direct access to source data/ documents. Furthermore, if an investigator refuses an inspection, his data will not be accepted in support of a New Drug Registration and/or Application.
- GSK Biologicals has a substantial investment in clinical studies. Having the highest quality data and studies are essential aspects of vaccine development. GSK Biologicals has a Regulatory Compliance staff that audit investigational sites. Regulatory Compliance assesses the quality of data with regard to accuracy, adequacy and consistency. In addition, Regulatory Compliance assures that GSK Biologicals sponsored studies are in accordance with the Good Clinical Practices and that relevant regulations/guidelines are being followed.
- To accomplish these functions, Regulatory Compliance selects investigational sites to audit. These audits usually take 1 to 2 days. The GSK Biologicals audits entail review of source documents supporting the adequacy and accuracy of CRFs, review of documentation required to be maintained, and checks on vaccine accountability. The GSK Biologicals audit therefore helps prepare an investigator for a possible regulatory agency inspection as well as assuring GSK Biologicals of the validity of the database across investigational sites.

The Inspector will be especially interested in the following items:

- Log of visits from the sponsor's representatives
- IRB/IEC approval
- Vaccine accountability
- Approved study protocol and amendments
- Informed consent of the subjects (written or witnessed oral consent)
- Medical records supportive of CRF data
- Reports to the IRB/IEC and the sponsor
- Record retention

GSK Biologicals will help investigators prepare for an inspection.

**VII. Confidentiality and Publication**

- WRAIR shall have the right to publish or permit the publication of any information or material relating to or arising out of the work after prior submission to GSK and USAID provided that if GSK or USAID shall so request WRAIR will delay publication for a maximum of six months to enable GSK and/or USAID to protect its rights in such information or material. Any proposed publication or presentation (e.g. manuscript, abstract or poster) for submission to a journal or scientific meeting, should be first submitted and agreed to by any other involved author(s) from WRAIR, USAMMDA, GSK and USAID prior to publication/presentation. GSK Biologicals and USAID will undertake to comment on such proposed documents within two weeks.

# Appendix C: Overview of the Recruitment Plan

**Part B**: Up to thirty (30) healthy adult volunteers aged 18 to 50 years, from the villages in the Kisumu West District and surrounding area, will be recruited for participation in the study. Prior to enrollment, an information campaign directed at the targeted population will be implemented. This will start with the administrative and senior leaders of the villages in the recruitment area around KC and expand to the barazas (town meetings) that everyone in the village can attend.

The sessions will explain the problem of malaria in this community, the current strategies for its control, as well as the limitations of these strategies. The need and the difficulties of developing a vaccine against malaria will be discussed, as well as an outline of the proposed trial, including the rationale, the background data available and the study objectives. Particular attention will be paid to study procedures including HIV testing, randomization to malaria vaccine or rabies and blood collection.

Next, Field Workers will be available in the health centers throughout Kisumu West District and surrounding areas to schedule volunteers for a briefing and coordinate transportation to the briefing. With this small a study we may be able to recruit everyone from around the CTC.

At the CTC, volunteers will be briefed on the study, as a group, in their native language. The briefing will explain the nature and purpose of the study and summarize the sections of the informed consent document. There will be a period for asking questions afterwards. Volunteers will then read the ICD and discuss the document, one-on-one with a nurse or clinician.

Afterwards, written informed consent will be obtained from each person who wants to participate in the study. So that language and illiteracy will not be impediments to informed consent, all briefings and explanations will be in the local language.

Screening may be done on a separate visit from recruiting/consenting.

# Appendix D: Vaccine Supplies, Packaging and Accountability

Under no circumstances are supplies permitted to be used for purposes other than those specified in the protocol. The sponsor will collect unused supplies on completion of the study. Used vaccine vials can be disposed on site according to local biosafety standards for disposal of biological waste material.

**1. Vaccine supplies**

**Part B:**

WRAIR will provide the USAMRU-K investigators with 60 vials of the antigen (FMP010), sufficient to administer three doses to all volunteers in the vaccine arm in Part B of the protocol. An additional three vials (5%) will be supplied for replacement in case of breakage or bad storage conditions. All vials/PFS need to be accounted for.

GSK Biologicals will provide the USAMRU-K investigators with 60 vials of the adjuvant AS01B, sufficient to administer three doses to all volunteers in the vaccine arm in Part B of the protocol. An additional three vials (5%) will be supplied for replacement in case of breakage or bad storage conditions. All vials/PFS need to be accounted for.

**2. Vaccine packaging**

The vaccine will be packed in labeled boxes. The box label will contain the following information: study number, subject number, lot number, and instructions for vaccine administration.

**3. Vaccine accountability**

The investigator or pharmacist must sign a statement that he/she has received the clinical supplies for the study. At any time the figures on supplied, used and remaining vaccine doses should match. At the end of the study, it must be possible to reconcile delivery records with those of used and returned stocks. Account must be given of any discrepancies.

After approval from the sponsor, USAID and GSK Biologicals, both used and unused vaccine vials will be destroyed at the study site using locally approved biosafety procedures and documentation unless otherwise described in the protocol. If no adequate biosafety procedures are available at the study site, the vaccine vials will be destroyed at an appropriate GSK site in accordance with GSK SOP CD-3.1 and SOP CD-7.1.

**4. Labels for sample identification**

The investigator will generate labels to identify samples taken from each subject at each time point. The label will contain the following information: study number, subject number, and sampling time point.

# Appendix E: Informed Consent Document

**PART B (KENYA)**

**PATIENT INFORMATION AND INFORMED CONSENT DOCUMENT**

You are asked to participate in a research study conducted by Dr. Nekoye from the Kenya Medical Research Institute (KEMRI) and Walter Reed Project (WRP). Walter Reed Project has its headquarters in the US. Your participation in this study is voluntary. You should read the information below and ask questions about anything that you do not understand before deciding whether or not to participate.

**WHAT IS THE NAME OF THE STUDY?**

Phase 1b controlled, double blind Study to Evaluate Safety, Reactogenicity, and Immunogenicity of the Candidate *Plasmodium falciparum* Malaria Protein 010 (FMP010) Administered Intramuscularly with GSK Biologicals’ Adjuvant AS01B in Healthy Malaria-Experienced Adults in Western Kenya.

**WHO ARE THE DOCTORS IN CHARGE OF THE STUDY?**

The study will be led by Dr Nekoye Otsyula and Dr Mark Polhemus of Kenya Medical Research Institute and Walter Reed Project, Kisumu, Kenya P.O. Box 54

Tel 0733 333 530

**AM I REQUIRED TO PARTICIPATE?**

No. Participation is completely voluntary.

1. If you choose NOT to participate, there will be no penalty to you.
2. If you choose to participate, you will be asked to sign a Consent Form.

You may withdraw from this study at any time. If you decide to withdraw from the study after it has started, we ask you to inform any staff member of the Walter Reed Project (WRP) or the doctor in charge. If you choose to withdraw from the study there will be no penalty for you or your family. If you withdraw after a vaccination you will be asked to complete the follow-up visits and blood work scheduled for that injection to ensure you do not have a reaction to the vaccine prior to withdrawing.

1. After you read this document, or have the document explained to you, take time to decide whether or not you want to take part. Please ask any questions that will help you fully understand the study.

Screening No

**WHAT IS THE PURPOSE OF THE STUDY?**

The main purpose of this study is to check if this new malaria vaccine is safe. In addition, the study will look at how your body responds to the vaccine.

**BACKGROUND**

Malaria is an illness that affects people in tropical countries around the world. *Plasmodium falciparum* is the most dangerous form of malaria and it is found around this area where you live. It can cause death in children and serious disease in adults. This parasite (*plasmodium falciparum)* no longer responds to many of the drugs that worked in the past. Presently there is no vaccine to protect against it. A new malaria vaccine has been developed and we would like to test it. We hope this vaccine will help prevent malaria.

Two thirds (2/3) of the people in the study will receive the new vaccine, and One third (1/3) will receive the rabies vaccine.

**WHAT DOES THE NEW VACCINE CONTAIN?**

**The vaccine in this study is a malaria vaccine for *Plasmodium falciparum*, named FMP010/AS01B. FMP010 is a protein designed after a part of the malaria parasite, but does not actually cause malaria infections. FMP010 will be mixed in liquid so the vaccine can be given as an injection. This liquid is called an adjuvant and is named AS01B. It is mixture of oil and water that may help stimulate your body to fight infections. This vaccine does not contain the actual malaria parasite, only a small part of it, and therefore you cannot get malaria from this vaccine.**

**The AS01B part of this vaccine has been used in other vaccines and been given to humans. No serious side effects have occurred in anyone who has been given this liquid adjuvant. The FMP010 part of the vaccine has been tested by itself in mice, guinea pigs, and rabbits without any serious side effects**

**This vaccine has been given to adults in the United States without serious side effects. This is the first time the vaccine will be given in Kenya.**

**KEMRI and the Kenya Ethical Review Committee have approved the use of this vaccine in this study.**

**WHY HAS THE RABIES VACCINE BEEN CHOSEN?**

Rabies is a very dangerous disease that can kill very quickly unless it is treated as soon as possible. . There is a vaccine for rabies, and you may receive it during this study. Even if you receive the complete course of the rabies vaccine, you will require additional doses if you come into contact with an animal which is thought to have rabies. It is important to understand that the vaccine does not cure rabies.

This means that if if you come into contact with an animal which you think has rabies, or if you are bitten or scratched by any animal during or after the trial, you must seek medical attention as quickly as possible from your nearest physician. The physician will be able to decide whether you need additional doses of rabies vaccine. If you need to see a doctor outside Kombewa Clinic because of rabies exposure you should let the study doctors at Kombewa Clinic know as soon as you can.

**WHAT ARE THE REQUIREMENTS FOR PARTICIPATION IN THIS STUDY?**

**You will be in a position to volunteer for the study if you are:**

- At least 18 years old and not older than 50
- In good general health
- Available to complete all the study visits over the next seven months
- Not breastfeeding and able to prevent pregnancy until two months after the last vaccination
- Able to understand and sign this informed consent document

**You will not be able to volunteer for a study if:**

- You have ever received an experimental malaria vaccine
- You have used any investigational or non-registered drug or vaccine other than the study vaccine within 30 days preceding the first dose of study vaccine, or if you plan to use them during the study period
- You have used drugs that alter the body’s ability to fight infection for a period of >14 days within the past 6 months. (examples include drugs used to treat cancer, steroids that can be swallowed as tablets such as prednisone, prednisolone, dexamethasone, etc)
- You have planned to have a vaccine not foreseen by the study protocol within 30 days of the first dose of the study vaccine.
- You have any confirmed or suspected disease that affects your body’s ability to fight infection including human immunodeficiency virus (HIV) infection
- You have a family history of inherited diseases that reduce the body’s response to disease
- You have longstanding or current neurologic disease including seizure disorder
- You have had your spleen removed
- Your physical examination and or laboratory shows longstanding or current, clinically significant disease of the lungs, heart, liver or kidney
- You are unwell at the time of enrollment
- You have an enlarged liver, right upper abdominal pain or tenderness
- You have received of immunoglobulins and/or have had transfusion of any blood products within the three months preceding the first dose of study vaccine or if you plan to have these during the study period
- As a woman, you are pregnant or breastfeeding.
- You are suspected or known to abuse alcohol or drugs abuse based on your history and physical examination
- As a woman, you are willing or plan to become pregnant during the study
- You have had an allergic reaction of any kind following previous immunization
- You are unable to make follow up visits
- You are unwilling to allow blood samples to be stored for future use
- You are allergic to kanamycin, imidazole (which are types of antibiotics/medicines that fight infection) or nickel (found in some types of jewelry)
- There is any other significant finding that is not already stated in the list above which in the opinion of the study doctor would increase your risk of having a bad outcome from participating in this study

**WHAT WILL BE DONE TO ME DURING THIS STUDY?**

If you volunteer to participate in this study, the following events will occur:

- You will be seen by a study clinician who will ask you about your medical history, perform physical examination and test your blood. This will be done to ensure you are in good health and eligible to participate in this study. Blood testing will include screening for HIV (the virus that causes AIDS).
- **If you are a woman, a screening pregnancy test will be conducted. If you are found to be pregnant, you will not be able to participate in the study. All women will be asked to avoid becoming pregnant until at least two (2) months after your last vaccination.**

- After the medical evaluation we will determine if you are eligible to enroll in the study.
- If you are not eligible to be in the study you will be told why. If it is because of a medical condition you will referred to a doctor that can help you.
- If you are eligible to be in the study we will notify you of when to come back for the first vaccination.
- When you come for the medical evaluation we will take a picture of you. If you are eligible to participate in this study, we will use the picture to create an identification card for you. You will use this identification card whenever you come to the Clinic, Follow-up Center, or Field Station. If you are not eligible to participate in this study, we will destroy the picture that was taken of you.
- You will be enrolled in the study once you receive the first vaccination. You could receive either the malaria vaccine or the rabies vaccine. There will be up to 30 volunteers in the study with 20 volunteers getting the malaria vaccine and 10 volunteers getting the rabies vaccine.
- The vaccine you receive will be decided by chance (like flip of the coin). Neither you nor the doctors working in the study will know which vaccine you have received until the end of the study. This is done to be sure that everyone in the study is evaluated and treated the same, without regard to the vaccine which you received.
- You will receive a total of three injections of the vaccine. Each dose of the malaria vaccine will be 0.5mls and the rabies vaccine will be 1ml. Acoloured masking tape will be used so that neither you nor the study staff will know what is being administered.
- The second dose will be given about one month after the first dose and the third dose about one month after the second dose. Each time you will receive the same vaccine you received for the first vaccination.
- The vaccine will be injected with a needle and syringe into the muscle of your upper arm.
- Prior to each injection, you will be asked about any changes in your medical condition to ensure you are still eligible to participate in the study.
- If you are a woman, you will be required to have a screening pregnancy test before each injection. A pregnancy test will also be done at any point during the study if you/ the study clinician suspect that you are pregnant.
- You must remain for observation for 30 minutes after each injection.
- Follow-up visits will be required on day one (1), day two (2), day three (3), day seven (7) and day fourteen (14) after each injection.
- During each follow-up visit the injection site will be evaluated and you will be asked about possible reactions to the vaccine.
- During some of the follow-up visits, we will draw blood from a vein in your arm to ensure there are no side effects from the vaccine and also to measure your body’s response to the vaccine.
- No more than 11 mL (about 2 teaspoonfuls) of blood will be obtained on any one day.
- You will also have follow-up visits at one month and two months after the last vaccination. These visits will include a brief medical evaluation and a blood draw to evaluate your medical condition and immune response to the vaccination.

The study will last seven months. For two months we will be screening volunteers and then there will be five months from the first vaccination to the final visit. In that period you will be required to come to the clinic a total of 20 times 11 of which there will be blood draws. A schedule of vaccinations, follow up visits, and blood draws will be provided. You should plan on two to three hours for each vaccination and one hour for each follow-up visit.

**AM I ALLOWED TO BECOME PREGNANT DURING THIS STUDY?**

Female participants are asked not to be pregnant at the beginning and during the study until at least two (2) months after the last injection of the study drug. This is because this vaccine has not been tested in pregnant women. You will be asked what method of pregnancy prevention you are using and will continue to use during the study period.

- **This vaccine has not been tested in pregnant women. To avoid becoming pregnant, you should either abstain from sexual relations or practice a method of birth control.** Acceptable methods include abstinence, and use of contraceptives including (but not limited to) intrauterine contraceptive device and oral contraceptives. **Except for surgical removal of the uterus, birth control methods such as those listed are not totally effective in preventing pregnancy. Urine pregnancy tests are not able to pick up very early pregnancies. Therefore, if the pregnancy test is negative but you still think that you might be pregnant, you should not participate in the study. The only way that you can guarantee that you are not pregnant is to not have sex with a male person.**
- **This vaccine has not been studied in males who get a female pregnant during the study, therefore we do not know if there would be any effects on that pregnancy. We would also recommend that men participating in this study use adequate birth control methods to prevent pregnancy while participating in this study.**
- **You should notify the investigator if you find out after the study that you became pregnant either during the study or within 60 days of the study.**
- **If you become pregnant while you are participating in the study, we will ask to be allowed to follow your pregnancy through its outcome. This will include information about both your health status and that of the child. The information will be reported confidentially (without names or identifying criteria) to KEMRI (Kenya Medical Research Institute),WRAIR (Walter Reed Army Institute of Research), USAMMDA (The U.S. Army Medical Materiel Development Activity), USAMRMC ORP (U.S. Army Medical Research and Materiel Command, Office of Research Protections), GlaxoSmithKline Biologicals, U.S. Agency for International Development (USAID) and the Food and Drug Administration (FDA).**
- If you become pregnant during the study we will not give you any more injections. We will continue to follow you until the baby is born. If at any time a change in your health or the baby’s health is discovered, the Principal Investigator should be notified immediately. You may be asked to complete study visit (s) for review of vaccine side effects and this may include a blood draw to check safety tests.

**WHAT PROBLEMS MIGHT OCCUR WITH PARTICIPATION IN THIS STUDY?**

The medical evaluation may uncover some important medical conditions that you may have. If any part of the medical evaluation results in you not being eligible for this study, you will be informed of the results by one of the physician investigators and you will be referred for medical care. At your request, we will inform your physician of any results we have.

The blood draw included in the medical evaluation will test for HIV (the virus that causes AIDS). You will be asked to sign a separate consent form for the HIV testing to ensure you understand the impact of this test.

You may have some side effects from the vaccine. This vaccine has been tested in humans from the United States but we still do not know everything about the vaccine. We expect that you may experience some pain and muscle swelling at the injection site.

Other reactions may occur within the first 72 hours after the vaccination. They include fever, chills, fatigue, headache, joint aches, muscle aches, nausea, and rash. Panadol or brufen may help to reduce the pain. You are however advised to visit our clinic and see the study staff if such reactions occur. You will be asked about these side effects on each follow-up visit.

As may happen with any vaccine, there is a small chance that you could have an allergic reaction due to either the malaria or rabies vaccines. Allergic reactions to some of the

medications that may be given (such as medications to treat malaria or other infections)

are also possible. An allergic reaction may be mild, such as a rash, or may be severe and

life-threatening. A life-threatening reaction to rabies vaccine is extremely rare (the

chances are about one in a million for commonly used vaccines). Qualified medical staff will always be on hand to monitor you for such reactions. In addition, medicines and

equipment needed to treat allergic reactions are available at the Clinic.

In addition to the potential risks and discomforts listed above, getting this vaccine may involve risks to you that are completely unexpected and unforeseen. This vaccine may also involve unexpected and unforeseen risks to a baby should you become pregnant during the study. For this reason, every time you come to the clinic, we will ask you about your health. Additionally, if you are unwell in between scheduled visits you are requested to contact the staff at WRP Kombewa clinic.

**WHAT SHOULD I DO IN CASE I AM ILL OR INJURED?**

If you think you have a medical problem, please report directly to the WRP Kombewa Clinic. If for some reason this is not possible, contact the staff at the WRP Kombewa Clinic by telephone: 0733-333-530.

You should seek all medical care through the WRP Kombewa Clinic. No treatments and no medications (including herbal medications) should be taken unless provided by the Clinic. The only exception to this is in the case of emergencies. If you need emergency medical attention outside the Clinic, please inform the study doctor straight away so that we may follow your condition properly. You should follow these instructions until we have told you that you are no longer eligible to participate in the study.

**HOW LONG WILL THE STUDY LAST?**

The period from when you receive the medical examination to the completion of the study is about seven months. This includes two months for screening and five months study time. During the vaccination phase you will have approximately 20 appointments at the Kombewa Clinic. Depending on the type of appointment, visits may take from 30 minutes to 4 hours. A schedule of visits is included for you at the end of this form.

**HOW MANY PEOPLE WILL BE ENROLLED?**

Up to 30 healthy, malaria-exposed adults aged 18-50 years will be enrolled.

**WHAT BENEFITS WILL I RECEIVE IF I PARTICIPATE IN THIS STUDY?**

You will receive free medical care from the day you are enrolled in the study until the end of the study or the time it is determined you are no longer eligible to continue in the study.

Free medical care will be provided through the WRP Clinic and the Kombewa District Hospital. This care will include treatment of any symptoms caused by the vaccine plus treatment for any acute illnesses like malaria and other infectious diseases. If you require medical care beyond the abilities of the WRP Clinic and Kisumu West District Hospital, we will transport you to a facility that can provide the care required and pay for your care there.

We will not provide medical care for illnesses or injuries that occurred before enrolling in this study. Dental care will not be provided.

After the study is finished, we will receive information about the treatment allocations. We will then know who received the malaria vaccine. We will give everyone who received the malaria vaccine the opportunity to also receive the rabies vaccine, free of charge.

**WILL I BEAR ANY COSTS FOR PARTICIPATING IN THE STUDY?**

1. There are no costs to you for taking part in this study. All tests examinations and medical care are provided to you free as part of the study. No other compensation (such as loss of wages or emotional distress) is offered by the sponsor.
2. You will be required to take your time, which may include time away from work, to visit us during your scheduled days or for further evaluation if you have abnormal labs during the study period.

**Other than medical care that will be provided, there is no other compensation available for your participation in this research. During scheduled visits, you will be transported to the research center and back home.**

**WHO WILL HAVE ACCESS TO MY INFORMATION?**

Information about your participation in this study, including doctor’s notes and lab results, will remain confidential. The records are secured in locked rooms and only WRP staff can have access to your study file. The information we collect may be reviewed by persons that are responsible to oversee this research that is, Representatives of the Kenya Medical Research Institute, the US Army Medical Research and Material Command (USAMRMC), the Walter Reed Army Institute of Research Institutional Review Board (WRAIR IRB) the U.S. Food and Drug Administration and Monitors and Auditors. Any report from this study will refer to you only by study identification number and not by name.

It is the policy of WRP’s higher headquarters that data sheets are to be completed on all volunteers participating in research for entry into the Volunteer Registry Data Base. The information to be entered into this confidential database includes your name, address, study name and dates. The intent of the data base is two-fold: first, to readily answer questions concerning an individual’s participation in research sponsored by USAMRMC; and second, to ensure that the USAMRMC can exercise its obligation to ensure research volunteers are adequately warned (duty to warn) of risks and to provide new information as it becomes available. The information will be stored at USAMRMC for a minimum of 75 years.

**WHAT WILL HAPPEN TO THE BLOOD THAT IS TAKEN FROM ME?**

During this study, you will be asked to provide blood samples that will be used to evaluate your health and how your body responds to this malaria vaccine. Portions of these samples may also be used in the future for purposes that are currently unknown. These samples will be stored at the Kisumu WRP. Samples may also be sent to WRP’s higher headquarters for further testing.

If your blood is to be used for research not included in this study the IRB of record will be notified and they will have to approve before your blood is used. You will not receive any notice of future uses of your samples.

A total of 87 (about 18 teaspoonfuls) will be drawn for the entire period of the study (7 months). This amount will be spread out to 11 different sample collection times. Not more than 3 teaspoonfuls will be taken at each given time.

**WHAT WILL HAPPEN IF I AM INJURED AS A RESULT OF BEING IN THIS STUDY?**

Should you be injured as a direct result of participating in this research project, you will be provided medical care, at no cost to you, for that injury. You will not receive any injury compensation, only medical care. This is not, however, a waiver or release of your legal rights. You should discuss this issue thoroughly with the principal investigator before you enroll in this study.

**CAN I BE WITHDRAWN FROM THE STUDY WITHOUT MY CONSENT?**

The principal investigator may withdraw you from participating in this study if you should:

- Develop a serious reaction to the vaccine
- Develop a health condition that would make further participation unsafe for you
- Fail to comply with the procedures outlined in this consent form.
- Do something that endangers or potentially harms a member of the study team.

**NEW FINDINGS**

The scientific results of this study will be the property of WRAIR, USAMMDA, USAID, and GSK. Once available, they will be explaned to those who are interested.

During the course of the study, you will be informed of any new findings (either good or bad) that might cause you to change your mind about continuing in the study. If new information is provided to you, your consent to continue participating in this study will be re-obtained.

**CONSENT FOR STORAGE OF SAMPLES FOR FUTURE USE**

As a participant in this Malaria Vaccine Study, you will be asked to voluntarily donate your blood samples to Kombewa Clinical Research Center. These samples will be used to ensure your safety and to test your body’s response to the malaria vaccine. It may also be used by the study investigators for uses not currently known to you. No Human Genetic (DNA) studies will be done with these samples. If any other use is considered, prior approval will be sought from KEMRI Ethical Review Committee (ERC). All safeguards ensuring privacy and confidentiality that are in place during this study period will also continue to be in place for the long-term storage of samples

**WHAT ARE MY RIGHTS AS A VOLUNTEER?**

**GlaxoSmithKline Biologicals (GSK BIO) is providing the adjuvant system (AS01B) and expert technical and monitoring assistance. This company’s main headquarters are in Europe. Therefore, they are required to use special wording in their research protocols. The European Union Data Protection Directive requires them to use this special wording. This wording is designed to ensure that you are aware of your rights. This wording is also designed to ensure that you are also clearly informed about how your personal data will be used and maintained confidentially, both during and after the research study is conducted.**

- 1. **Your personal data, including data relating to your health, will be recorded and processed for the purpose of assessing the outcome of the study. Processing will be done by WRP/GSK or may be contracted to a third party under strict confidentiality rules. Your data may also be processed for product registration and for notification to organizations monitoring the safety and effectiveness of medicines. Your data may also be processed in order to add to scientific knowledge;**
  2. **Your participation in the study will be treated as confidential. You will not be referred to by name in any report concerning the study, nor will your identity be disclosed to any person, other than in circumstances such as where a need arises to check the correctness or completeness of data, or where there is a need to provide such information to regulatory agencies responsible for registration and safety of medicines.)**
  3. **Your medical data may be sent to and processed by any affiliate of GSK in any country inside or outside the European Union, always respecting the requirements of the EU Data Protection Directive (95/46/EC) and/or the equivalent applicable law.**
  4. **You may access your personal data and have any justifiable corrections made. If you wish to do so, you should request this from the doctor conducting the study. You agree to the postponement of your access to your medical data up to the completion of the study, including analysis and reporting of data, if deemed appropriate by the doctor conducting the study in order to safeguard the aim and conduct of the study. Representatives of KEMRI, USAMMDA, USAMRMC, GSK or regulatory bodies for medicines may access your records from this study.**

**WHO CAN I CONTACT FOR INFORMATION OR ANSWERS TO QUESTIONS CONCERNING THE RIGHTS OF A RESEARCH PARTICIPANT?**

You may contact the Secretariat of the Kenya National Ethical Review Committee, C/O

Kenya Medical Research Institute, P.O. Box 54840, Nairobi, Kenya Tel. 020-2722541.

**IF YOU WOULD LIKE MORE TIME TO THINK ABOUT THE STUDY, OR**

**WISH TO DISCUSS IT WITH YOUR SPOUSE, FEEL FREE TO TAKE THE**

**CONSENT FORM HOME WITH YOU. IF YOUR SPOUSE HAS QUESTIONS**

**OR CONCERNS, WE WILL BE HAPPY TO SPEAK WITH HIM/HER.**

**IF THERE ARE ANY PARTS OF THE CONSENT FORMS THAT YOU DO NOT**

**UNDERSTAND, PLEASE ASK THE INVESTIGATOR BEFORE SIGNING.**

**IF YOU HAVE QUESTIONS OR CONCERNS AT ANY TIME AFTER SIGNING**

**THE CONSENT FORM, FEEL FREE TO SPEAK WITH THE STUDY**

**DOCTORS.**

**YOU WILL RECEIVE A COPY OF THIS CONSENT FORM TO KEEP AFTER**

**IT IS COMPLETED.**

**CONSENTING TO ACCEPT PARTICIPATION IN THE STUDY**

I have read the information provided above. I have been given an opportunity to ask questions and all of my questions have been answered to my satisfaction.

I am aware that my photo for identification in this study will be taken.

|  |
| --- |

I am aware that my blood samples will be stored for future use

|  |
| --- |

Name of Subject

Thumb Print if subject is unable to sign

_________ ____________________ __________

Signature of Subject Date

**_______________________________________________**

Witness’s Name(If the subject cannot read or write)

____________________________ _____________

Witness’s Signature Date

_______________________________________________

Name of Consenting Staff

____________________________ ______________

Signature of Consenting Staff Date

________________________________________________

Name of Investigator/Designee

Signature of Investigator/Designee Date

Screening No

**CONSENT FOR HIV TEST**

In addition to the other blood tests, we would like your permission to do a test to find out if you have HIV (human immunodeficiency virus) infection. HIV is a type of germ that can cause AIDS (acquired immunodeficiency syndrome), which is usually a serious health problem and can be deadly. Someone can look and feel perfectly healthy and still be infected. The only way to know is by doing a blood test. Over time HIV infection decreases the body’s fighting power and increases a person’s risk of catching other diseases, including diarrhea and tuberculosis. In order to help you decide whether or not you wish to be tested for HIV infection, you will be offered an opportunity for counseling. Please discuss with the counselor, before making your decision.

The reason why we would like to test if you have HIV infection or not, is that HIV infection may change the response to the vaccine we are testing, and this study is only testing the vaccine in HIV negative persons. Therefore it is important for us to have HIV results on the blood samples taken during screening.

The benefit of HIV testing is that you will receive counseling and if you agree, you will be given the results of these tests. If the test is found to be positive, another test will be done to confirm your status. If the 2^nd^ test is also found to be positive, you will be offered an opportunity for referral to a Ministry of Health facility of your choice for continued care.

All information that you provide will be considered confidential, and no mention of your name or any other identifying information will appear on the samples sent to the laboratory or in any publication in connection with this study. The blood samples will be coded so that your name will not be revealed. No persons other than the counselors and research doctors overseeing your care will have access to any information that identifies you individually. The samples will be processed in the KEMRI/Walter Reed Project Laboratory in Kisumu to ensure complete confidentiality and minimize the risk of disclosures.

If you have any questions you may contact any study team member, Walter Reed Project, P.O. Box 54, Kisumu, Kenya, at cell phone: 0733-333-530.

Screening No

## *Consent for HIV test*

The need for HIV testing has been explained to me. It has been explained to me that the test is required for participation in the study. I have had the opportunity to ask questions about it and any questions that I have asked have been answered to my satisfaction. I consent voluntarily to be tested for HIV and I have been informed that I can choose to decline the test.

_________________________________________________________

Name of Subject

________________________________________ ___________

Signature of Subject (thumbprint if unable to sign) Date

Thumb print if volunteer unable to sign

**_______________________________________________**

Witness’s Name(If the subject cannot read or write)

____________________________ _____________

Witness’s Signature Date

_______________________________________ ____________

Printed Name of Counsellor Date

________________________________________ ____________

Signature of Counselor giving consent explanation Date

________________________________________ ______________

Investigator’s name Date

__________________ ____________

Investigator’s signature Date

Screening No

**Volunteer Schedule**

| **Visit#** | **Study Day** | **Scheduled Visit  Date** | **Time Allotment**  **(approximate)** | **Activity** |
| --- | --- | --- | --- | --- |
| 1 | Screening | 75 to 1 days prior to start | 2.5to 5hours | - Briefing (Information presented about the study; discussion period afterwards fro questions and answers) - Written Informed Consent - Photograph taken and stored - Temperature, Heart Rate and blood pressure monitored - Medical History and Physical examination - Pregnancy test for all female adult volunteers - Blood draw (1.5 tea spoons) |
| **2** | **0** |  | **Vaccination # 1,**  **4 hours** | - **Temperature, Heart Rate and blood pressure monitored** - **Medical History and Physical examination** - **Exclusion/Inclusion criteria reviewed** - **Blood draw (2 teaspoons)** - **Women: Urine pregnancy test (*if your test is positive, you will not be vaccinated).*** - **Vaccination 1 followed by 30 min evaluation** |
| 3-6 | 1,2, 3 ,7 |  | 30 minutes | - See physician for interim history, including any medications taken - Blood draw on day 7 |
| 7 | 14 |  | 30 minutes | - See physician for interim history, including any medications taken - Blood draw (1.5 teaspoons) |
| **8** | **28** |  | **Vaccination # 2,**  **4 hours** | - **Temperature, Heart Rate and blood pressure monitored** - **Medical History and Physical examination** - **Exclusion/Inclusion criteria reviewed** - **Blood draw (2 teaspoons)** - **Women: Urine pregnancy test (*if your test is positive, you will not be vaccinated).*** - **Vaccination 2 followed by 30 min evaluation** |
| 9-12 | 29, 30,31, 35 |  | 30 minutes each day | - See physician for interim history, including any medications taken   Blood draw day 35 (1.5 teaspoons) |
| 13 | 42 |  | 30 minutes | - See physician for interim history, including any medications taken - Blood draw (1.5 teaspoons) |
| **14** | **56** |  | **Vaccination # 3,**  **4 hours** | - **Temperature, Heart Rate and blood pressure monitored** - **Medical History and Physical examination** - **Exclusion/Inclusion criteria reviewed** - **Blood draw (2 teaspoons)** - **Women: Urine pregnancy test (*if your test is positive, you will not be vaccinated).*** - **Vaccination 3 followed by 30 min evaluation** |
| 15-18 | 57, 58, 59,63 |  | 30 minutes each day | - See physician for interim history, including any medications taken - Blood draw day 63 (1.5 teaspoons) |
| 19 | 70 |  | 30 minutes | - See physician for interim history, including any medications taken - Blood draw (1.5 teaspoons) |
| 20 | 86 |  | 30 minutes | See physician for interim history, including any medications taken. |
| 21 | 112 |  | 30 minutes | - See physician for interim history, including any medications taken - Blood draw (1.5 teaspoons) - **Urine pregnancy test** - Study close-out visit |

# Appendix F: List of investigators and study contributors and their roles and responsibilities

| **Names** | **Study roles and responsibilities** |
| --- | --- |
| Mark Polhemus M.D.  Nekoye N. Otsyula M.D. | **Co-Principal Investigators.** Physicians responsible for the overall conduct of the study |
| Michele Spring, MD | **Protocol Champion.** Principal Investigator, Part A. Associate Investigator Part B. |
| Douglas Walsh, COL, MC  Bernhards Ogutu, M.D.  Walter Otieno, MD  Lucas Otieno, M.D  Milton Omondi, M.D | **Sub-investigators.**  Physicians whose role is to participate in participant recruitment, screening, vaccination, follow-up of study participants. |
| John Waitumbi , Ph D  Ann V. Stewart, Ph D | **Lab Associate Investigators.**  Responsible for coordination of research assays. |
| Carter Diggs, MD, Ph.D. | Responsible for funding of MSP1 antigen development (preclinical and clinical) |
| Lorraine Soisson, Ph.D. | Responsible for funding of MSP1 antigen development (preclinical and clinical) |
| Amos Otedo, MD. | Responsible for evaluating safety of vaccine in volunteers and assessing unexpected and/or serious adverse events |

**APPENDIX G- Amendment** **2**

| Item  Section Number | Version 11  25 March 2009 | Comments  (reason for change) |
| --- | --- | --- |
| Throughout Document | Version # and date added to footer | **Document control** |
| Page 34  Audits and inspections | Knowledge of any pending compliance inspections/visit by the FDA, OHRP, or other government agency concerning clinical investigation or research, the issuance of Inspection Reports, FDA Form 483, warning letters or actions taken by any Regulatory Agencies including legal or medical actions and any instances of serious or continuing noncompliance with the regulations or requirements will be reported immediately to the KEMRI ERC and WRAIR IRB. WRAIR IRB will report to USAMRMC ORP/HRPO as per SOP UWZ-C-636 |  |
| Page 47  **Significant Protocol Deviations** | Significant deviations that occur in greater than minimal risk protocol will be reported to KEMRI IRB within the first 24 hours and also promptly reported (within 48 hours) to the WRAIR IRB by phone (301 319-9940), by email (WRAIRDHSP@amedd.army.mil), or by facsimile (301-319-9961) to the Division of Human Subjects Protection, Walter Reed Army Institute of Research, 503 Robert Grant Ave., RM 1W30, Silver Spring, Maryland, 20910-7500, All reports will be submitted with a cover memo naming the protocol, KEMRI SSC number, WRAIR and HSRRB log numbers, the principal investigator, the time period covered, and any exceptional events that occurred. |  |
| **Page 61**  **SAE Reporting** | Unanticipated problems involving risk to volunteers or others, serious adverse events related to participation in the study and all volunteer deaths should be promptly reported by the investigator to the sponsor, KEMRI IRB, GSK Biologicals’ Clinical Safety Physician at Tel :+32 2 656 8850, Fax: +32 2 656 51 16 or +32 2 656 80 09; or mobile phones for 7/7 day availability: +32 477 404 713; e-mail: [rix.ct-safety-vac@gskbio.com](mailto:rix.ct-safety-vac@gskbio.com) or Head Safety Evaluation and Risk Management, Adult/Adolescent/Emerging Diseases at phone : +32472 906 600, or Head of Safety Evaluation and Risk Management, Pediatric, at mobile phone: +32 474 53 48 68(24/24 hour and 7/7 day availability) and to WRAIR IRB by phone (301 319-9940), by email (WRAIRDHSP@amedd.army.mil), or by facsimile (301-319-9961) to the Division of Human Subjects Protection, Walter Reed Army Institute of Research, 503 Robert Grant Ave., RM 1W30, Silver Spring, Maryland, 20910-7500. WRAIR IRB will report to USAMRMC ORP/HRPO as per SOP UWZ-C-636 within 24 hours (one calendar day) of his/her becoming aware of the event. | **All are primary contacts for SAE reporting.** |
| Page Page 63  Suspensions, Clinical Hold or Terminations | Any suspensions (to include continuing review lapses), clinical holds (voluntary or involuntary), or terminations of this research by an IRB, the institution, the Sponsor, or regulatory agencies will be promptly to the KEMRI ERC and WRAIR IRB. WRAIR IRB will report to USAMRMC ORP/HRPO as per SOP UWZ-C-636. |  |
| **Page 64:**  **Contact Information for Serious Adverse Event s Reporting** | GSK Biologicals Clinical Safety Physician  Tel: +32 2 656 8850  **Fax: +32 2 656 51 16 or +32 2 656 80 09**  Mobile phones for 7/7 day availability:  +32 477 404 713  (Head Safety Evaluation and Risk Management, Adult/Adolescent/Emerging  Diseases)  +32472 906 600  (Head Safety Evaluation and Risk Management, Pediatric)  Back-up mobile phone contact:  +32 474 53 48 68  24/24 hour and 7/7 day availability | **Added** |
